# LncRNA AC007255.1, an immune-related prognostic enhancer RNA in esophageal cancer

**DOI:** 10.7717/peerj.11698

**Published:** 2021-07-14

**Authors:** Qingqing Wang, Xiaoyan Yu, Ningning Yang, Lu Xu, Yunfeng Zhou

**Affiliations:** 1 Department of Radiation Oncology and Medical Oncology, Zhongnan Hospital, Wuhan University, Wuhan, China; 2 Hubei Cancer Clinical Study Center, Hubei Key Laboratory of Tumor Biological Behaviors, Zhongnan Hospital, Wuhan University, Wuhan, China; 3 Department of Ultrasound, Zhongnan Hospital of Wuhan University, Wuhan, China

**Keywords:** Esophageal cancer, AC007255.1, PRR15, Enhancer RNA, Prognosis, Immune infiltration

## Abstract

**Background:**

Growing evidence has suggested that enhancer RNAs (eRNAs), a set of long non-coding RNAs (lncRNAs) that were derived from active enhancer regions, play critical roles in regulating gene expression in human cancers. Nevertheless potential functions of eRNAs in esophageal cancer ESCA have not yet been expounded. Here, this study aimed to explore key prognostic eRNAs in ESCA.

**Methods:**

LncRNAs that were transcribed from active enhancer regions were analyzed utilizing the PreSTIGE algorithm, followed by prediction of their target genes. Based on the ESCA RNA-seq data from the TANRIC database, overall survival (OS)-related eRNAs were determined. The correlation between AC007255.1 expression and various clinical traits of ESCA was calculated. Functional enrichment analysis was presented based on its co-expressed genes. Based on the TIMER database, we analyzed correlations between AC007255.1 expression and immune infiltration levels. qRT-PCR was utilized to validate the expression of AC007255.1 and PRR15 in ESCA and normal tissues.

**Results:**

Totally, 2,695 lncRNAs were transcribed from active enhancer regions. Among them, 33 were significantly related to OS. AC007255.1 was a key eRNA. PRR15 was a target gene of AC007255.1 (correlation coefficient *r* = 0.936). Patients with high AC007255.1 expression indicated poor OS time. There were significant correlations between AC007255.1 expression and clinical characteristics like pathological TNM, grade and stage. AC007255.1 was closely related to tight junction and neutrophil activation involved in immune response. Moreover, AC007255.1 expression was related to the infiltration levels of B cell, dendritic cell and neutrophil. qRT-PCR results confirmed that AC007255.1 and PRR15 were both up-regulated in ESCA tissues, and there was a positive correlation between the two.

**Conclusion:**

Our findings identified a novel immune-related eRNA AC007255.1 in ESCA, which could be a promising prognostic factor for ESCA.

## Introduction

Esophageal cancer (ESCA) represents a common gastrointestinal malignancy globally, with a poor prognosis (Feng et al., 2019; Miller et al., 2019; Siegel, Miller & Jemal, 2019). ESCA mainly includes two histological subtypes, squamous cell carcinoma and adenocarcinoma (Rustgi & El-Serag, 2015). Surgery prolongs the survival time of patients at an early stage. Unfortunately, only about 25% of diagnosed patients are suitable for surgery due to tumor location and late diagnosis (Chen et al., 2018). Currently, for patients who cannot undergo surgery, chemotherapy such as combination of 5-fluorouracil, cisplatin and taxanes is the optimal treatment option (Batra et al., 2019). Despite the progression of targeted therapy, ESCA patients’ prognosis is still poor, and the 5-year survival rate is between 20% and 35% (Kelly, 2019). Thus, it is crucial to identify novel biomarkers to predict ESCA patients’ prognosis.

Long non-coding RNAs (lncRNAs) are transcripts over 200 nucleotides in length. LncRNAs are expressed in a tissue- or cell-specific manner (Xie et al., 2018). Abnormal expression of lncRNAs is related to the initiation and progression of ESCA by participating in various molecular mechanisms (Hu et al., 2019; Liang et al., 2018). Transcription control is a key step in gene expression regulation, enhancers are DNA elements that can bind to transcription factors (Garcia-Gonzalez et al., 2016). Recently, it has been discovered that enhancers can also transcribe non-coding RNA (ncRNA), called as eRNA (Li, Notani & Rosenfeld, 2016). eRNA is a set of ncRNA transcribed from the transcription enhancer region by RNA polymerase II, as the main cis-regulatory element in the genome (Liu, 2017). Among them, some lncRNAs have been found to be transcribed from the active enhancer region and retained in the nucleus, thereby participating in gene expression regulation (Huang et al., 2018). Thousands of eRNAs have been identified in human cells, many of which have been shown to mediate the activation of target genes (Zhang et al., 2019b). In humans, eRNAs are involved in various signaling pathways by mediating their target genes such as clinically operable genes as well as immune checkpoints, indicating a promising clinical application in eRNA-targeted therapy (Zhang et al., 2019b). Despite the key role of eRNA in gene transcription control, the potential functions of eRNA in ESCA have not yet been developed.

In this study, we determined prognostic eRNAs and their target genes in ESCA. We found that lncRNA AC007255.1 was highly expressed in ESCA tissue, which was significantly related to of ESCA patients’ prognosis. PRR15 was a potential target gene of AC007255.1. AC007255.1 expression was distinctly correlated to immune infiltration in ESCA. Thus, AC007255.1 could be an underlying prognostic marker for ESCA.

## Materials and Methods

### Download of a list of eRNAs

The lncRNAs that were transcribed from active enhancer regions from the Encyclopedia of DNA Elements database (ENCODE) was referenced from Vučićević ’s previously identified, and their target genes were predicted using the PreSTIGE algorithm (Vučićević et al., 2015). By the Ensembl BioMart, Ensembl transcript ID was converted to gene symbol.

### Differential expression analysis

Transcriptome expression data, clinical and follow-up information of 33 kinds of cancers were downloaded from The Cancer Genome Atlas (TCGA) using the UCSC Xena website (Haeussler et al., 2019). The RNA-sequence (RNA-seq) data of AC007255.1 were analyzed between cancer and normal samples between 33 kinds of cancers and corresponding normal tissues by the edgeR package in R (Robinson, McCarthy & Smyth, 2010). —Log 2fold change (FC)— ≥1 and *p* < 0.05 was considered statistically significant.

### Co-expression analysis

Based on the TANRIC co-expression data, correlation between putative eRNAs and their predicted target genes was analyzed (Li et al., 2015). The co-expressed genes of AC007255.1 were screened in ESCA by Spearman correlation analysis under the threshold of Spearman’s rank correlation coefficient *r* > 0.4 and *p* < 0.001. Furthermore, correlation between AC007255.1 and PRR15 was also analyzed in 33 kinds of cancers.

### Functional enrichment analysis

Functional enrichment analysis of 4140 target genes (Spearman’s rank correlation coefficient *r* > 0.4 and *p* < 0.001) of AC007255.1 was performed via the clusterProfiler package in R (Ashburner et al., 2000). Gene Ontology (GO) enrichment analysis was presented to explore biological process (BP), cellular component (CC) and molecular function (MF) of AC007255.1 (Ashburner et al., 2000). Furthermore, signaling pathways related to AC007255.1 were analyzed using Kyoto Encyclopedia of Genes and Genomes (KEGG) pathway enrichment analysis (Kanehisa & Goto, 2000). Adjusted *p* < 0.05 was considered significantly enriched.

### Tumor Immune Estimation Resource (TIMER)

The correlations between the expression of AC007255.1 or PRR15 and the infiltration abundance of six immune cells (B cells, CD4 + T cells, CD8 + T cells, neutrophils, macrophages and dendritic cells) were estimated by TIMER (version 2.0; https://cistrome.shinyapps.io/timer/) (Li et al., 2017; Li et al., 2020).

### Real-time fluorescent quantitative PCR (RT-qPCR)

Totally, 12 pairs of esophageal cancer tissues and adjacent normal tissues were collected from Zhongnan Hospital of Wuhan University (Wuhan, China) between 2014 and 2016. The inclusion criteria were as follows: (1) the patient did not receive radiotherapy and chemotherapy before surgery; (2) the patient was diagnosed as esophageal cancer by pathology, imaging, and clinical. This research strictly followed the ethical principles of medical research involving human subjects in the Declaration of Helsinki. Our study gained the approval of the Medical Ethics Committee of Zhongnan Hospital of Wuhan University (No. 2020171) Tissues were lysed by Trizol (15596-026; Ambion, Austin, Texas, USA). Total RNA was extracted, and RNA purity and concentration were assessed by JY02S UV analyzer (Beijing Junyi Dongfang Electrophoresis Equipment Co., Ltd., China). Then, RNA was reverse transcribed into cDNA. PCR was achieved by QuantStudio 6 RT-qPCR instrument (ABI, USA). Primers of target genes were synthesized by Beijing Qingke Biological Technology Co., Ltd. (China), as follows: homo AC007255.1: 5′-GTCTTGTTCTGCTACCCTCCA-3′(forward), 5′-GCTCCACATTCACTTTCCATA-3′(reverse); homo PRR15: 5′-TGGAAATCGCTCAC CAACAG-3′(forward), 5′-AGATCTTCAAATTGCGGCGG-3′(reverse); homo GAPDH: 5′-TCAAGAAGGTGGTGAAGCAGG-3′(forward), 5′-TCAAAGGTGGAGGAGTGGGT-3′(reverse). Reaction procedures were as follows: predenaturation: a cycle for 10 min at 95 °C; transsexual: 40 cycles for 15 s at 95 °C; annealing extension: 40 cycles for 60 s at 60 °C and 40 cycles for 15 s at 95 ° C; melt curve: a cycle for 60 s at 60 °C and a cycle for 15 s at 95 °C. The relative expression levels were normalized by the 2^−ΔΔ^CT) method.

### Statistical analysis

Kaplan–Meier overall survival (OS) analysis was performed to assess the expression of eRNAs and prognosis of 33 kinds of cancers via the Atlas of Noncoding RNAs in Cancer (TANRIC) (Li et al., 2015). Key eRNAs were determined if they were significantly related to OS (*p* < 0.05) and their target genes (*p* < 0.001) in ESCA. The difference in AC007255.1 expression was analyzed in different clinical traits including cancer status (tumor free and with tumor), age (<60 and ≥60), race (Asian, black African American and White), reflux history (no and yes), tumor central location (distal, mild and proximal), pathological N stage (N0, N1, N2 and N3), grade (G1, G2 and G3) and stage (stage I, stage II, stage III and stage IV) by non-parametric Kruskal–Wallis H test or Wilcoxon signed-rank test. Based on the median value of AC007255.1 expression, cancer samples were classified into high and low expression groups. The chi-square test was utilized to analyze whether various clinical characteristics were related to AC007255.1 expression. The differences in expression of AC007255.1 or PRR15 were assessed between 12 ESCA and 12 normal tissues by paired student’s t test. A two-sided *p*-value  <  0.05 was considered statistically significant. The correlation between AC007255.1 and PRR15 was calculated by Spearman correlation analysis.

## Results

### Identification of eRNAs associated with ESCA prognosis

Totally, 2695 lncRNAs that were transcribed from active enhancer regions were retrieved from ENCODE database by the PreSTIGE algorithm (The ENCODE Project Consortium, 2012; Vučićević et al., 2015). Moreover, their 2303 target genes were predicted, which have been identified in a previous study (Vučićević et al., 2015). Using the Ensembl BioMart, transcript ID was converted to gene ID. We found that 2695 putative eRNAs were matched to their corresponding 1288 genes. Based on ESCA-related RNA-seq expression profiles in TCGA database, Kaplan–Meier overall survival analysis results showed that 33 lncRNAs derived from enhancers were significantly related to overall survival of patients with ESCA (Table S1). Among them, the potential targets of these lncRNAs were predicted with the threshold of Spearman’s rank correlation coefficient *r* > 0.4 and *p* < 0.001 (Table 1).

**Table 1:** Eleven pairs of overall survival-related lncRNAs derived from enhancers and target genes.

eRNA symbol	Overall Survival Analysis, Log-Rank *p*-Value	Predicted target	Correlation between eRNA and Neighboring target	
			Correlation Coefficient *r*	*p*-value
AC007255.1	0.039	PRR15	0.936	<0.001
SLC44A3-AS1	0.041	SLC44A3	0.830	<0.001
FOXP4-AS1	0.049	FOXP4	0.769	<0.001
AC025871.2	0.018	FBXO16	0.697	<0.001
AL021391.1	0.027	RIBC2	0.676	<0.001
AP000696.1	0.041	SIM2	0.651	<0.001
LINC01006	0.003	RNF32	0.651	<0.001
LINC01271	0.045	CEBPB	0.526	<0.001
CCDC18-AS1	0.049	CCDC18	0.508	<0.001
SPAAR	0.041	HRCT1	0.476	<0.001
WDFY3-AS2	0.008	WDFY3	0.445	<0.001

### LncRNA AC007255.1 is a key eRNA of ESCA

**Figure 1:**
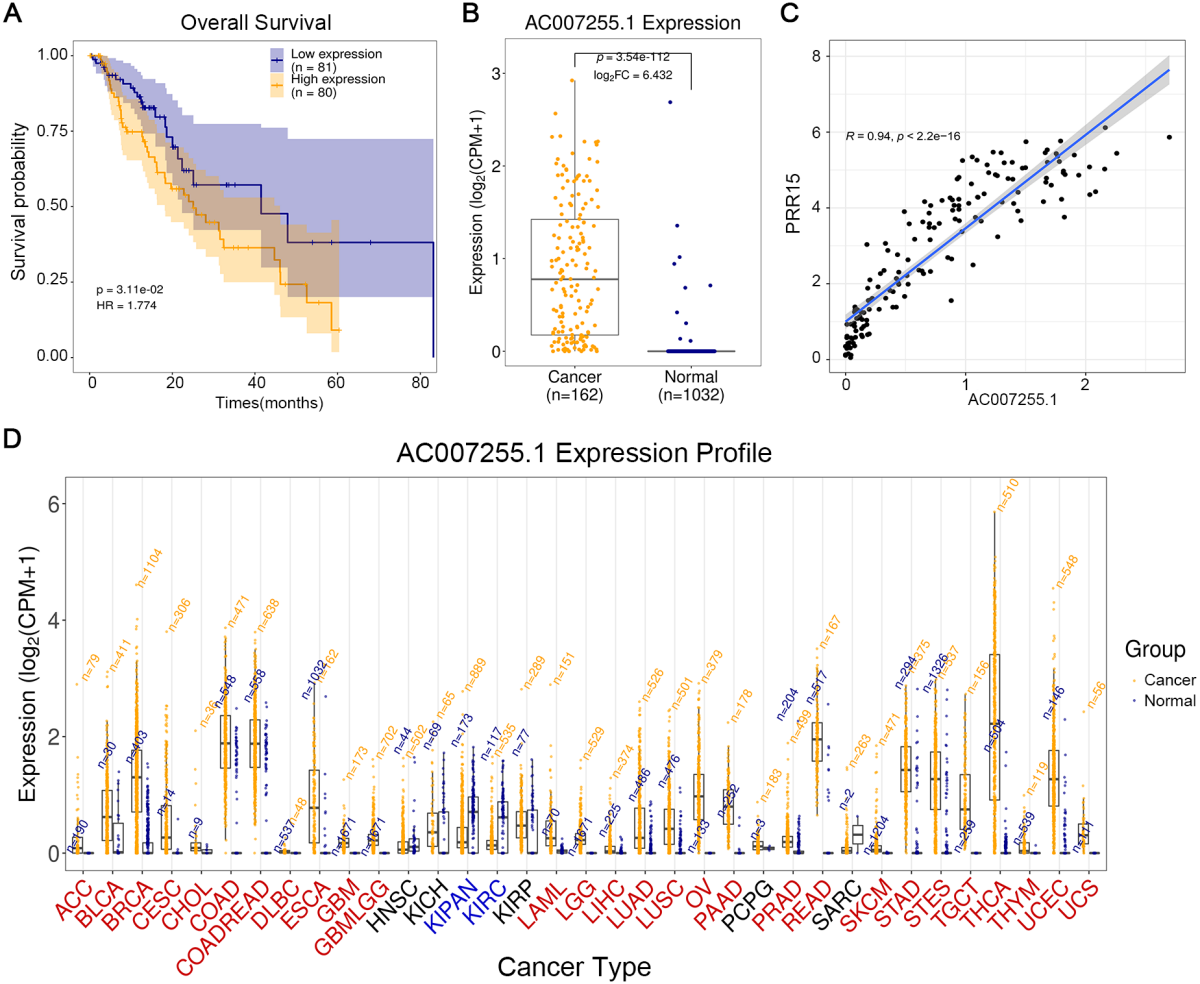
LncRNA AC007255.1 is a key eRNA of ESCA. (A) Kaplan–Meier overall survival between high AC007255.1 expression and its low expression among 161 ESCA patients. (B) Box plots depicting the expression of AC007255.1 between ESCA and normal samples. (C) Spearman correlation analysis between AC007255.1 and PRR15. (D) Expression patterns of AC007255.1 across 33 kinds of cancers.

eRNA-target genes were sorted by the correlation coefficients, we found there was the strongest correlation between AC007255.1 and PRR15. Among ESCA patients, high AC007255.1 expression indicated worse OS time than its low expression (*p* = 3.11e−02 and HR = 1.774; Fig. 1A). AC007255.1 expression was significantly up-regulated in 162 ESCA tissues in comparison to 1,032 normal tissues (*p* = 3.54e−112 and log2FC = 6.432; Fig. 1B). We further analyzed target genes that had co-expression relationships with AC007255.1 in 162 ESCA (Spearman’s rank correlation coefficient *r* > 0.4; *p* < 0.001, Table S2). Among them, we found that PRR15 was a target gene with the largest correlation coefficient (*r* = 0.94 and and *p* < 2.2e−16; Fig. 1C). The expression patterns of AC007255.1 in 33 kinds of cancers were further analyzed. As shown in Fig. 1D, AC007255.1 was significantly highly expressed in most of cancers than normal tissues. Also, we analyzed the prognostic value of AC007255.1 expression in different kinds of cancers. In Table 2, in addition to ESCA, AC007255.1 expression was significantly related to OS of mesothelioma (MESO), acute myeloid leukemia (LAML; *p* = 0.002), lung adenocarcinoma (LUAD; *p* = 0.003), uterine corpus endometrial (UCEC; *p* = 0.025) and thyroid carcinoma (THCA; *p* = 0.031). Moreover, in most kinds of cancers, AC007255.1 expression was significantly correlated to PRR15 expression. Thus, among all OS-related eRNAs, AC007255.1 was identified as a key eRNA for ESCA.

**Table 2:** Overall survival analysis and gene expression correlations for AC007255.1 and PRR15 across 33 kinds of cancers from TCGA database.

Tumor type		AC007255.1 and Overall Survival Log-Rank *p*-Value	AC007255.1 and PRR15	
Abbreviation	Detail		Correlation Coefficient	Correlation *p*-Value
MESO	Mesothelioma	0.001	0.929	<0.001
LAML	Acute Myeloid Leukemia	0.002	0.553	<0.001
LUAD	Lung adenocarcinoma	0.003	0.881	<0.001
UCEC	Uterine Corpus Endometrial Carcinoma	0.025	0.746	<0.001
THCA	Thyroid carcinoma	0.031	0.938	<0.001
ESCA	Esophageal carcinoma	0.039	0.936	<0.001
PCPG	Pheochromocytoma and Paraganglioma	0.058	0.136	0.066
STAD	Stomach adenocarcinoma	0.067	0.690	<0.001
SKCM	Skin Cutaneous Melanoma	0.069	0.321	<0.001
LUSC	Lung squamous cell carcinoma	0.084	0.891	<0.001
CHOL	Cholangiocarcinoma	0.114	0.669	<0.001
CESC	Cervical squamous cell carcinoma and endocervical adenocarcinoma	0.132	0.913	<0.001
PRAD	Prostate adenocarcinoma	0.134	0.651	<0.001
GBM	Glioblastoma multiforme	0.139	0.147	0.057
SARC	Sarcoma	0.169	0.533	<0.001
THYM	Thymoma	0.203	0.846	<0.001
UVM	Uveal Melanoma	0.206	0.061	0.592
ACC	Adrenocortical carcinoma	0.248	0.867	<0.001
BRCA	Breast invasive carcinoma	0.270	0.820	<0.001
READ	Rectum adenocarcinoma	0.289	0.625	<0.001
BLCA	Bladder Urothelial Carcinoma	0.294	0.883	<0.001
LIHC	Liver hepatocellular carcinoma	0.319	0.677	<0.001
KICH	Kidney Chromophobe	0.331	0.925	<0.001
PAAD	Pancreatic adenocarcinoma	0.347	0.768	<0.001
DLBC	Lymphoid Neoplasm Diffuse Large B-cell Lymphoma	0.419	0.380	0.008
HNSC	Head and Neck squamous cell carcinoma	0.445	0.815	<0.001
TGCT	Testicular Germ Cell Tumors	0.463	0.838	<0.001
UCS	Uterine Carcinosarcoma	0.499	0.705	<0.001
KIRC	Kidney renal clear cell carcinoma	0.539	0.421	<0.001
LGG	Brain Lower Grade Glioma	0.644	0.091	0.037
OV	Ovarian serous cystadenocarcinoma	0.670	0.866	<0.001
KIRP	Kidney renal papillary cell carcinoma	0.751	0.773	<0.001
COAD	Colon adenocarcinoma	0.758	0.680	<0.001

### AC007255.1 expression is distinctly related to various clinical traits of ESCA

**Figure 2:**
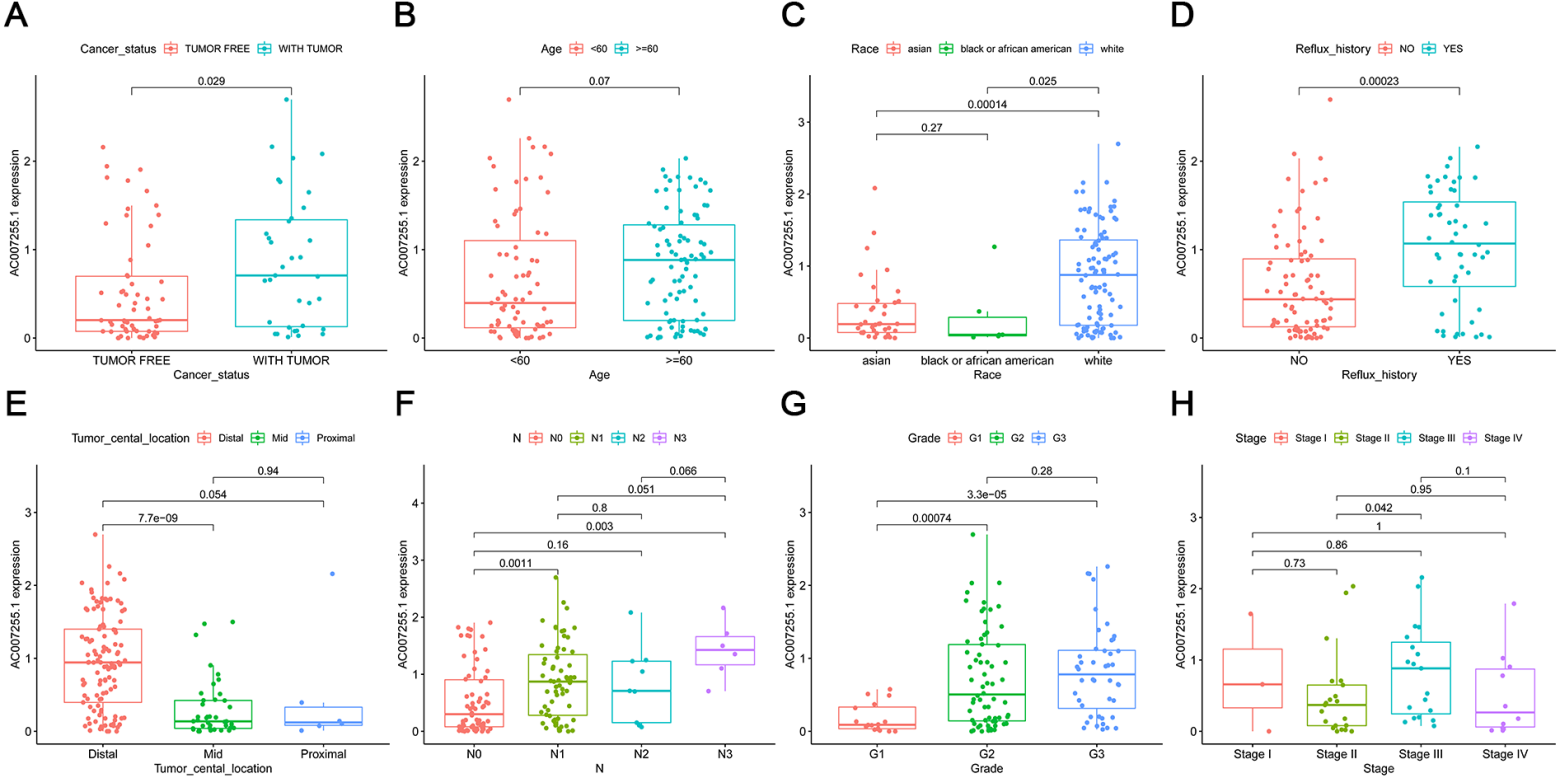
Correlation between AC007255.1 expression and clinical traits of ESCA. (A) Cancer status; (B) age; (C) race; (D) reflux history; (E) tumor central location; (F) pathological N stage; (G) grade and (H) stage.

Table S3 listed the clinical characteristics of 161 ESCA patients from TCGA database including age, stage, tumor central location, grade, TNM, cancer status, reflux history, gender and race. The correlation between AC007255.1 expression and these clinical traits was further assessed. In Fig. 2A, AC007255.1 expression was related to tumor status (*p* = 0.029), and patients with tumor had higher AC007255.1 expression than those without tumor. There was no significant difference in its expression between <60 and ≥60 years old (*p* = 0.07; Fig. 2B). Among different races, white patients had the highest AC007255.1 expression (*p* = 0.00014 or *p* = 0.025; Fig. 2C). As shown in Fig. 2D, patients with esophageal reflux had higher expression of AC007255.1 than those without reflux history (*p* = 0.00023). Furthermore, AC007255.1 expression was significantly related to tumor central location. Its higher expression was found in patients with distal ESCA than mid (*p* = 7.7e−09; Fig. 2E). In Fig. 2F, the higher the N stage, the higher the expression of AC007255.1. The grade was positively correlated with the expression of AC007255.1 (Fig. 2G). In Fig. 2H, patients with stage III had significantly higher AC007255.1 expression than those with stage II (*p* = 0.042). These findings indicated that AC007255.1 expression was related to the severity of ESCA. A total of 161 ESCA patients were divided into high and low expression groups based on the median value of AC007255.1 expression. Chi-square test results showed that AC007255.1 expression was significantly correlated to stage (*p* < 0.001), pathological T stage (*p* = 0.001), pathological N stage (*p* = 0.001), pathological M stage (*p* < 0.015) and race (*p* < 0.001) in Table 3.

**Table 3:** Correlation between AC007255.1 expression and clinicopathologic characteristics of ESCA patients.

Characteristics	High expression (80)	Low expression (81)	*χ*^2^	*p* value
Age			3.933	0.047
<60	30	44		
≥60	50	37		
Stage			21.144	<0.001
Stage I	9	8		
Stage II	21	48		
Stage III	30	19		
Stage IV	7	1		
unknown	13	5		
Pathology T stage			18.654	0.001
T1	19	8		
T2	10	27		
T3	40	37		
T4	0	4		
unknown	11	5		
Pathology N stage			25.748	<0.001
N0	18	48		
N1	40	23		
N2	6	3		
N3	6	0		
unknown	10	7		
Pathology M stage			8.354	0.015
M0	53	68		
M1	7	1		
unknown	20	12		
Gender			1.740	0.187
female	8	15		
male	72	66		
Race			30.144	<0.001
asian	6	32		
black or africanamerican	1	4		
white	58	42		
unknown	15	3		

### Biological functions of AC007255.1 and its correlation to immune cell infiltration

**Figure 3:**
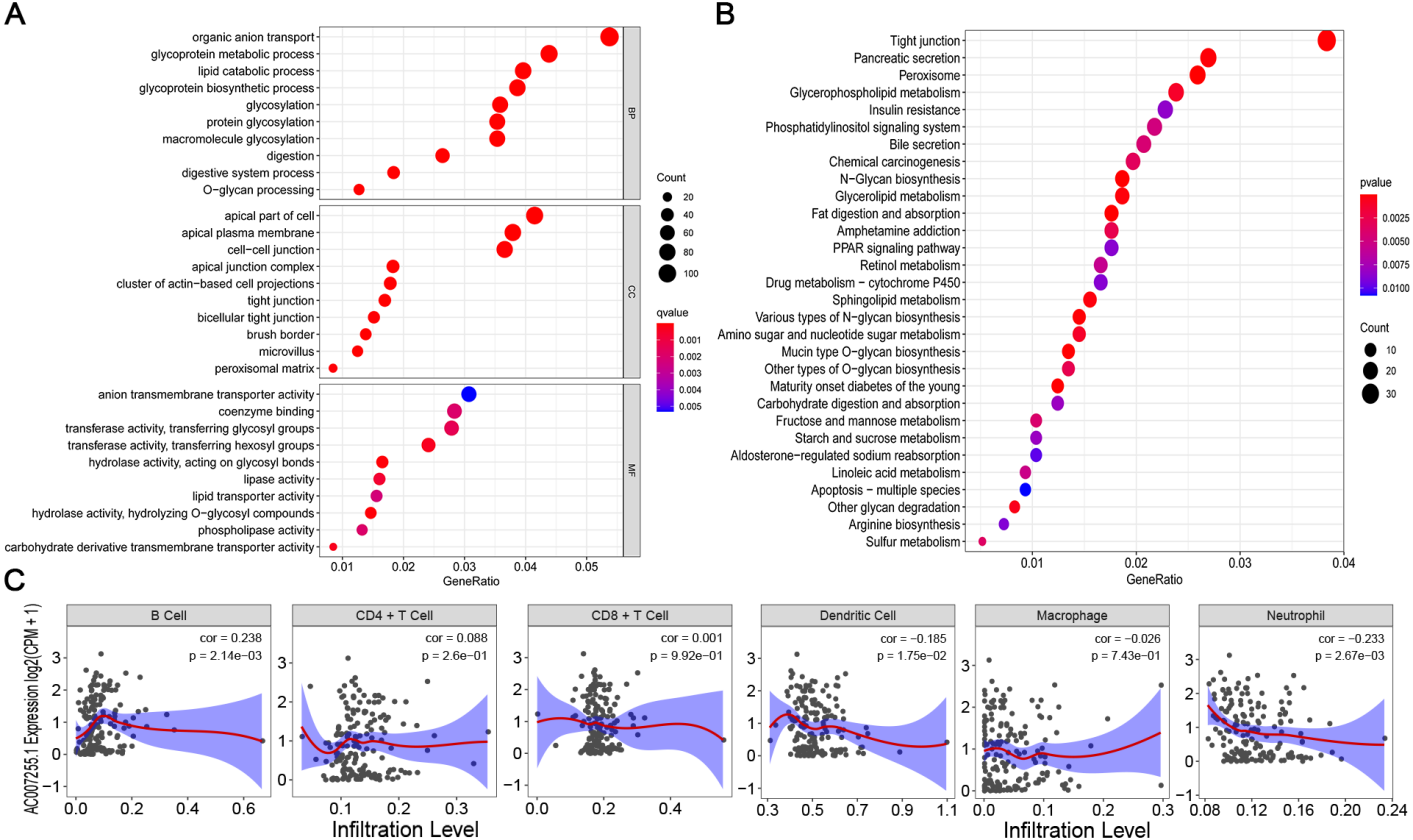
Biological functions of AC007255.1 and its correlation to immune cell infiltration. (A) The top 10 GO enrichment analysis results including biological process (BP), cellular component (CC) and molecular function (MF). (B) The top 30 KEGG pathway enrichment analysis results. The size of the bubble is proportional to the number of enriched genes. The color from red to blue indicates that the *p* = 7-value is from small to large. (C) Correlation between AC007255.1 expression and the infiltration levels of six immune cells by TIMER.

The biological functions of 4140 target genes (Spearman’s rank correlation coefficient *r* > 0.4 and *p* < 0.001, Table S2 ) of AC007255.1 were analyzed by GO and KEGG enrichment analysis. As shown in Figs. 3A, 3B, we found that the target genes were significantly enriched a variety of biological processes and signal pathways. Among them, AC007255.1 was closely connected with tight junction. In the tight junction, 37 related genes were enriched (Spearman’s rank correlation coefficient *r* > 0.4 and adjusted *p* < 0.001; Table 4). In addition, AC007255.1 was distinctly related to neutrophil activation involved in immune response. In the signaling pathway, 78 related genes were enriched (Spearman’s rank correlation coefficient *r* > 0.4 and adjusted *p* < 0.05; Table S4 ). The correlation between AC007255.1 and the infiltration levels of immune cells was analyzed by TIMER. In Fig. 3C, the results showed that AC007255.1 expression was positively correlated to B cell infiltration (cor = 0.238 and *p* = 2.14e−03). Also, its expression was negatively related to dendritic cell (cor = −0.185 and *p* = 1.75e−02) and neutrophil infiltration (cor = −0.233 and *p* = 2.67-03). However, there was no significant difference between AC007255.1 expression and CD4+T cell (cor = 0.088 and *p* = 2.6e−01), CD8+T cell (cor = 0.001 and *p* = 9.92e−01) and macrophage (cor = −0.026 and *p* = 7.43e−01) infiltration.

**Table 4:** Thirty-seven genes associated AC007255.1 that are enriched in tight junction (Spearman’s rank correlation coefficient *r* >0.4 and adjusted *p* < 0.05).

Gene Symbol	Spearman Correlation Coefficient r	Gene Symbol	Spearman Correlation Coefficient r	Gene Symbol	Spearman Correlation Coefficient r
CLDN3	0.764				
LLGL2	0.753	CLDN4	0.624	MARVELD3	0.526
CGN	0.745	CLDN7	0.623	CLDN18	0.520
TJP3	0.736	MAGI1	0.597	MARVELD2	0.520
CFTR	0.700	CLDN9	0.596	SRC	0.520
PARD6B	0.692	AMOT	0.586	MICALL2	0.514
OCLN	0.679	MYH14	0.585	CLDN15	0.503
DLG3	0.675	GATA4	0.575	MAPK8	0.490
CLDN23	0.672	ERBB2	0.569	PRKAA1	0.489
PRKAB1	0.652	VASP	0.564	CRB3	0.467
CACNA1D	0.645	ROCK2	0.562	AFDN	0.458
CLDN2	0.635	EPB41L4B	0.561	EZR	0.450
PARD6A	0.633	TJP2	0.548	RAPGEF2	0.439

### Validation of AC007255.1 and PRR15 expression in ESCA

**Figure 4:**
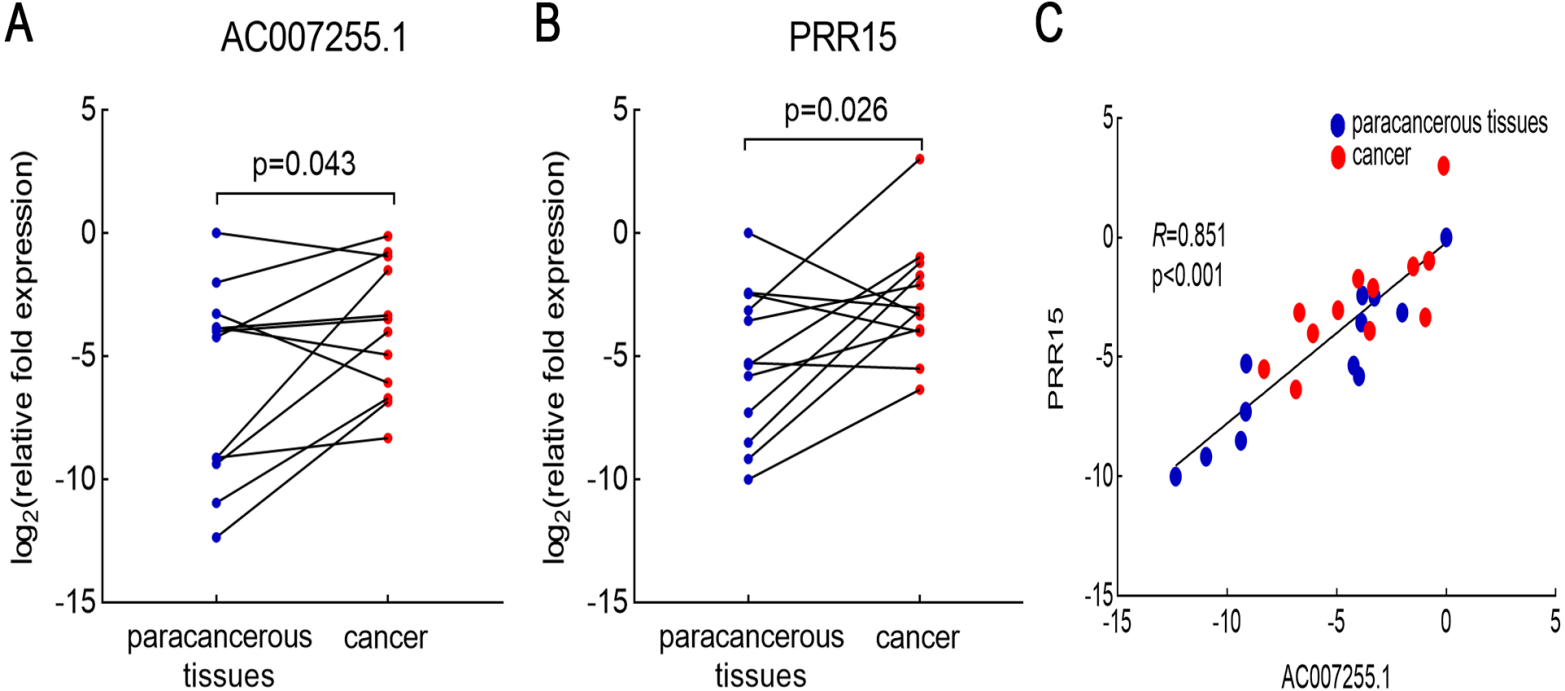
Validation of AC007255.1 and PRR15 expression in ESCA tissues by RT-qPCR. AC007255.1 (A) and PRR15 (B) expression was quantified in 12 pairs of ESCA and normal tissues. (C) Spearman correlation analysis between AC007255.1 and PRR15 expression. Red dot indicates ESCA samples and blue dot indicates normal samples.

AC007255.1 and PRR15 expression levels were validated between 12 pairs of ESCA and normal tissues by RT-qPCR. Consistent with bioinformatics analysis results, AC007255.1 was distinctly up-regulated in 12 ESCA than 12 normal tissues (*p* = 0.043; Fig. 4A). Furthermore, higher PRR15 expression was detected in 12 ESCA tissues compared to 12 normal tissues (*p* = 0.026; Fig. 4B). Spearman correlation analysis results indicated that AC007255.1 expression was in a positive correlation with PRR15 expression (*R* = 0.851; and Fig. 4C).

### Correlation between PRR15 expression and prognosis and immune cell infiltration

**Figure 5:**
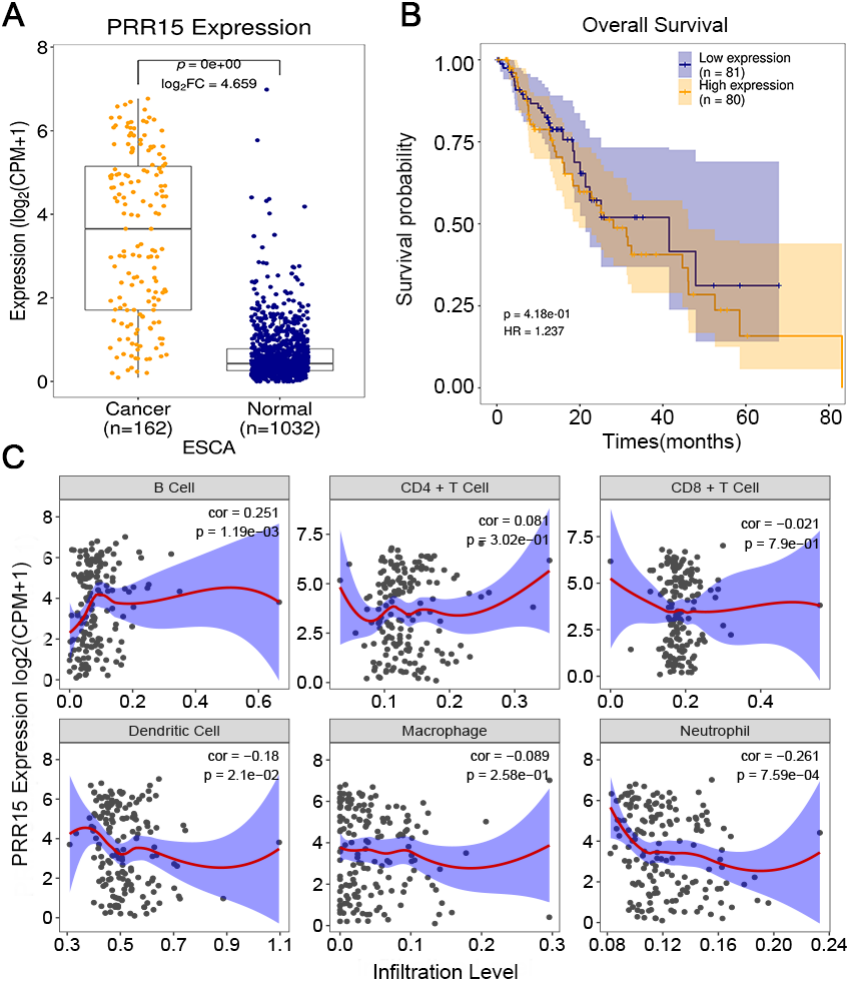
Correlation between PRR15 expression and prognosis and immune cell infiltration. (A) Box plot of the expression of PRR15 in ESCA and normal tissues. (B) Kaplan–Meier overall survival between high and low expression of PRR15 among ESCA patients. (C) Correlation between PRR15 expression and the infiltration levels of six immune cells by TIMER.

Consistently, PRR15 exhibited distinctly higher expression in 162 ESCA tissues than 1032 normal tissues (*p* < 0.0001; Fig. 5A). Furthermore, its high expression indicated poorer prognosis of patients compared to its low expression (*p* = 4.18e−01; Fig. 5B). By TIMER database, we analyzed the correlation between PRR15 expression and immune cell infiltration. Our data showed that PRR15 expression was distinctly correlated to the infiltration levels of B cell (cor = 0.251 and *p* = 1.19e−03), dendritic cell (cor = −0.18 and *p* = 2.1e−02) and neutrophil (cor =−0.261 and *p* = 7.59e−04) in Fig. 5C. However, there was no significant correlation between PRR15 expression and CD4+ T cell, CD8+ T cell and macrophage.

## Discussion

In this study, we tried to identify lncRNAs associated with ESCA as potential biomarkers for prognosis and treatment. For the first time, our results showed that AC007255.1, an eRNA, was dysregulated in ESCA tissue. In addition, there was a positive correlation between AC007255.1 and PRR15, which may be related to ESCA progression.

Recently, many studies have shown that certain lncRNAs, which are transcribed from the active enhancer region, can regulate gene expression via interaction with transcription factors. It is estimated that there are >55,000 possible enhancers in the human genome, indicating the complexity of the role of enhancers in gene regulation (Heintzman et al., 2009). In this study, we found that AC007255.1 was significantly positively correlated with its target gene PRR15, suggesting that it may have a positive regulatory effect. Herein, eRNA AC007255.1 was distinctly up-regulated in ESCA. The up-regulation was significantly correlated to several clinical traits including cancer status, reflux history, tumor central location, stage, grade, pathological TNM stage and race. Also, AC007255.1 expression had a positive correlation with the severity of ESCA. Patients with its high expression indicated a poorer prognosis than those with its low expression. Thus, AC007255.1 was a promising prognostic factor for ESCA.

AC007255.1 expression had a strong positive correlation to PRR15 expression in ESCA, indicating that PRR15 could be a target gene for eRNA AC007255.1. Both high expressions were associated with poor prognosis. It has been confirmed that eRNAs participate in cancer progression by gene expression regulation (Heintzman et al., 2009). Our data indicated that AC007255.1 might participate in ESCA progression by regulating its potential target gene PRR15. PRR15 is a member of proline rich protein family, which is almost exclusively expressed in post-mitotic cells during fetal development (Purcell et al., 2009) and in adult tissues (Meunier et al., 2011). Furthermore, it is expressed in mouse and human gastrointestinal tumor tissues (Meunier et al., 2011). Wnt pathway activation could be related to abnormal expression of PRR15 (Meunier et al., 2011). PRR15 can enhance the viability and survival of trophoblasts in the early stages of pregnancy (Gates et al., 2017). Genome-wide analysis of DNA methylation in bronchial lavage fluid has exhibited a distinct PRR15 CpG methylation for patients with non-small cell lung cancer (Um et al., 2018). Smoking cessation may reduce the DNA methylation level of PRR15 in non-malignant bronchial epithelial cells. Co-expression network analysis has indicated PRR15 is a critical gene during PHY906 and CPT11 combined treatment of colon cancer (Xing et al., 2020). These studies have demonstrated that PRR15 is expressed in a tissue-specific manner. Also, its abnormal expression could contribute to the development of cancers. Herein, our results displayed that PRR15 was highly expressed in ESCA tissues, which might be regulated by eRNA AC007255.1.

Tumor cells can play immune escape by recruiting various immune cells in the tumor microenvironment (Lin et al., 2016). Immunotherapy such as tumor vaccination and immune checkpoint suppression is a promising new approach for ESCA therapy. However, ESCA immunotherapy often generates mixed results, partly because of the lack of reliable markers that can predict therapy response (Zhang et al., 2019a). Increasing evidence has emphasized the key regulatory roles of lncRNAs in the immune system (Zhang et al., 2019a). For example, lncRNA TCL6 is related to immune infiltration of breast cancer, indicating poorer survival time (Zhang et al., 2020). In this study, AC007255.1 expression was positively correlated to B cell infiltration. B cells may facilitate tumorigenesis including ESCA via recruiting inflammatory cells as well as inducing the expression of angiogenic factors (Schwartz, Zhang & Rosenblatt, 2016). We found that AC007255.1 expression was in a negative relationship with dendritic cell infiltration. Dendritic cell vaccination is the main drug candidate for immunotherapy, which has been shown to induce an immune response to enhance infiltration of lymphocytes (VanWilligen et al., 2018). Recently, it has been observed that dendritic cell vaccination could prolong the median progression-free survival and overall survival of patients with advanced or recurrent ESCA (Ogasawara et al., 2020). Moreover, a negative correlation between neutrophil infiltration and AC007255.1 expression was detected in ESCA. The ratio of neutrophil to lymphocytes (NLR) is a prognostic marker of ESCA. Recent studies also have found that NLR is closely related to immunosuppression (Chen et al., 2019). LncRNAs is an important regulator of neutrophils for cancers. For instance, lncRNA HOTTIP induces the immune escape of ovarian cancer cells through up-regulation of PD-L1 in neutrophils (Shang et al., 2019). Moreover, we found that PRR15 expression was significantly associated with the infiltration levels of B cell, dendritic cell, and neutrophil. More experiments should be carried out to validate the interaction of PRR15 expression with B cell, dendritic cell, and neutrophil in ESCA. Our function enrichment analysis indicated that AC007255.1 was closely correlated to tight junction. Tight junction proteins play a vital role in maintaining the integrity of cells (Qin, Fang & Fang, 2017). Imbalance of the tight junction barrier can induce cancer cells to infiltrate and even metastasize (Kuo et al., 2016). Consistent with TIMER analysis results, AC007255.1 was distinctly related to neutrophil activation involved in immune response. These results indicated that AC007255.1 could participate in ESCA-related key signaling pathways.

Although we verified the expression of AC007255.1 and PRR15 in ESCA, this study still has some limitations. Firstly, the roles of AC007255.1 in ESCA should be validated *in vitro* and *in vivo* experiments. Secondly, we did not fully describe the causal relationship between AC007255.1 and PRR15. The regulatory mechanisms of AC007255.1 and PRR15 should be validated in ESCA cells. Thirdly, clinical implications of AC007255.1 should be investigated in a larger cohort of ESCA. RNA targeted drugs are now becoming a major new branch of drugs. Extensive studies on targeting disease-related RNAs are ongoing in the pharmaceutical industry. Herein, we identified a novel eRNA AC007255.1 associated with ESCA and further proved its prognostic value. AC007255.1 possessed potential as a prognostic and therapeutic target for ESCA.

## Conclusions

Taken together, our findings demonstrated that eRNA AC007255.1 was up-regulated in ESCA tissue. High AC007255.1 expression indicated a poorer prognosis for ESCA patients. Furthermore, AC007255.1 might exacerbate ESCA progression via activating the expression of PRR15. In short, our study provides a new insight into understanding the pathogenesis of ESCA.

##  Supplemental Information

10.7717/peerj.11698/supp-1 Supplemental Information 1 Enhancer lncRNAs related to overall survival of patients with ESCA

10.7717/peerj.11698/supp-2 Supplemental Information 2 Target genes of AC007255.1 in ESCA (Spearman’s rank correlation coefficient *p* and *r* > 0.4)

10.7717/peerj.11698/supp-3 Supplemental Information 3 Clinicopathological characteristic information of ESCA patients from TCGA database

10.7717/peerj.11698/supp-4 Supplemental Information 4 78 genes associated AC007255.1 that are enriched in neutrophil activation involved in immune response (Spearman’s rank correlation coefficient *p* < 0.001 and adjusted *r* > 0.4)

10.7717/peerj.11698/supp-5 Supplemental Information 5 Raw data: the original ct values of Fig. 4 (Validation of AC007255.1 and PRR15 expression in ESCA tissues by qRT-PCR.)

## Abbreviations

LncRNAs: long non-coding RNAs
eRNAs: enhancer RNAs
ESCA: esophageal cancer
OS: overall survival
ncRNA: non-coding RNA
TCGA: The Cancer Genome Atlas
RNA-seq: RNA-sequence
GO: Gene Ontology
BP: biological process
CC: cellular component
MF: molecular function
KEGG: Kyoto Encyclopedia of Genes and Genomes
TIMER: Tumor Immune Estimation Resource
RT-qPCR: Real-time fluorescent quantitative PCR
TANRIC: Noncoding RNAs in Cancer

## Additional Information and Declarations

### Data Availability

The following information was supplied regarding data availability: The raw data is available in the Supplementary Files and the following sites: 1. The datasets used from UCSC Xena: TCGA-ACC, TCGA-BLCA, TCGA-BRCA, TCGA-CESC, TCGA-CHOL, TCGA-COAD, TCGA-DLBC, TCGA-ESCA, TCGA-GBM, TCGA-HNSC, TCGA-KICH, TCGA-KIRC, TCGA-KIRP, TCGA-LAML, TCGA-LGG, TCGA-LIHC, TCGA-LUAD, TCGA-LUSC, TCGA-MESO, TCGA-OV, TCGA-PAAD, TCGA-PCPG, TCGA-PRAD, TCGA-READ, TCGA-SARC, TCGA-SKCM, TCGA-STAD, TCGA-TGCT, TCGA-THCA, TCGA-THYM, TCGA-UCEC, TCGA-UCS, TCGA-UVM.
https://xenabrowser.net/datapages/. 2. The keywords used from TGGA: primary site, esophagus, program name, TCGA, project id, TCGA-ESCA, workflow type, HTSeq-FPKM, data category, transcriptome profiling, data type, gene expression quantification.
https://portal.gdc.cancer.gov/. 3. The ENCODE datasets came from Vučićević et al., 2015
