# Supplementary material for: LncRNA AC007255.1, an immune-related prognostic enhancer RNA in esophageal cancer
Source: PeerJ. 2021 Jul 14;9:e11698. doi: 10.7717/peerj.11698 (PMC8286057; doi:10.7717/peerj.11698)
Supplement: Supplemental Information 1 [file peerj-09-11698-s001.docx]

| Table S1. Enhancer lncRNAs related to overall survival of patients with ESCA. | | | | | |
| --- | --- | --- | --- | --- | --- |
| eRNA symbol | Overall Survival Analysis, Log-Rank *p*-Value | eRNA symbol | Overall Survival Analysis, Log-Rank *p*-Value | eRNA symbol | Overall Survival Analysis, Log-Rank *p*-Value |
|  |  |  |  |  |  |
| AL390879.1 | 0.002 | AF241728.1 | 0.022 | FTX | 0.038 |
| LINC01006 | 0.003 | AC005515.1 | 0.022 | AC007255.1 | 0.039 |
| KCP | 0.007 | AC245140.1 | 0.023 | AP000696.1 | 0.041 |
| AP000943.1 | 0.008 | TMEM225B | 0.023 | SLC44A3-AS1 | 0.041 |
| WDFY3-AS2 | 0.008 | AP001781.1 | 0.025 | AC022613.1 | 0.042 |
| LINC01714 | 0.014 | AC092490.1 | 0.026 | AC108749.1 | 0.042 |
| AC012368.1 | 0.014 | AL021391.1 | 0.027 | TMEM88B | 0.045 |
| AC025871.2 | 0.018 | AP001596.1 | 0.029 | AC021028.1 | 0.045 |
| AP000688.3 | 0.020 | C1orf61 | 0.029 | LINC01271 | 0.045 |
| AC098828.2 | 0.020 | AP003469.2 | 0.034 | CCDC18-AS1 | 0.049 |
| AC098934.1 | 0.021 | JPX | 0.035 | FOXP4-AS1 | 0.049 |
