# Supplementary material for: LncRNA AC007255.1, an immune-related prognostic enhancer RNA in esophageal cancer
Source: PeerJ. 2021 Jul 14;9:e11698. doi: 10.7717/peerj.11698 (PMC8286057; doi:10.7717/peerj.11698)
Supplement: Supplemental Information 2 [file peerj-09-11698-s002.docx]

| Table S2. Target genes of AC007255.1 in ESCA (Spearman’s rank correlation coefficient r>0.4 and p<0.001). | | | |
| --- | --- | --- | --- |
| eRNA symbol | Target gene | Correlation Coefficient *r* | *p*-value |
| AC007255.1 | PRR15 | 0.936 | < 0.001 |
| AC007255.1 | AC007255.2 | 0.816 | < 0.001 |
| AC007255.1 | CHN2 | 0.803 | < 0.001 |
| AC007255.1 | FAM221A | 0.801 | < 0.001 |
| AC007255.1 | PLEKHA6 | 0.795 | < 0.001 |
| AC007255.1 | MYO5C | 0.793 | < 0.001 |
| AC007255.1 | ICA1 | 0.790 | < 0.001 |
| AC007255.1 | AC006042.1 | 0.788 | < 0.001 |
| AC007255.1 | ARFGEF3 | 0.787 | < 0.001 |
| AC007255.1 | TMEM62 | 0.787 | < 0.001 |
| AC007255.1 | HID1 | 0.781 | < 0.001 |
| AC007255.1 | PLS1 | 0.779 | < 0.001 |
| AC007255.1 | AC008870.6 | 0.778 | < 0.001 |
| AC007255.1 | C9orf152 | 0.777 | < 0.001 |
| AC007255.1 | LINC01342 | 0.777 | < 0.001 |
| AC007255.1 | BCL2L15 | 0.776 | < 0.001 |
| AC007255.1 | AC130456.7 | 0.775 | < 0.001 |
| AC007255.1 | PRR15L | 0.773 | < 0.001 |
| AC007255.1 | AC008870.1 | 0.771 | < 0.001 |
| AC007255.1 | TRIM31-AS1 | 0.770 | < 0.001 |
| AC007255.1 | AC127526.2 | 0.768 | < 0.001 |
| AC007255.1 | RHPN2 | 0.767 | < 0.001 |
| AC007255.1 | AL117382.1 | 0.767 | < 0.001 |
| AC007255.1 | GPR160 | 0.765 | < 0.001 |
| AC007255.1 | CLDN3 | 0.764 | < 0.001 |
| AC007255.1 | LONRF3 | 0.764 | < 0.001 |
| AC007255.1 | AKR7L | 0.762 | < 0.001 |
| AC007255.1 | EPCAM | 0.762 | < 0.001 |
| AC007255.1 | AC130456.2 | 0.762 | < 0.001 |
| AC007255.1 | CRACR2B | 0.761 | < 0.001 |
| AC007255.1 | AL592114.3 | 0.761 | < 0.001 |
| AC007255.1 | AKR7A3 | 0.760 | < 0.001 |
| AC007255.1 | TMC5 | 0.760 | < 0.001 |
| AC007255.1 | PLUT | 0.759 | < 0.001 |
| AC007255.1 | TMEM63A | 0.758 | < 0.001 |
| AC007255.1 | TOX3 | 0.758 | < 0.001 |
| AC007255.1 | ARHGAP12 | 0.758 | < 0.001 |
| AC007255.1 | AC007490.1 | 0.757 | < 0.001 |
| AC007255.1 | PIP5K1B | 0.755 | < 0.001 |
| AC007255.1 | TRIM31 | 0.753 | < 0.001 |
| AC007255.1 | LLGL2 | 0.753 | < 0.001 |
| AC007255.1 | DNMBP-AS1 | 0.753 | < 0.001 |
| AC007255.1 | MIR559 | 0.752 | < 0.001 |
| AC007255.1 | AC130456.3 | 0.751 | < 0.001 |
| AC007255.1 | LINC01814 | 0.749 | < 0.001 |
| AC007255.1 | ARHGEF38 | 0.749 | < 0.001 |
| AC007255.1 | MLPH | 0.748 | < 0.001 |
| AC007255.1 | SYTL2 | 0.747 | < 0.001 |
| AC007255.1 | CGN | 0.745 | < 0.001 |
| AC007255.1 | TMEM125 | 0.743 | < 0.001 |
| AC007255.1 | USH1C | 0.743 | < 0.001 |
| AC007255.1 | LINC02747 | 0.742 | < 0.001 |
| AC007255.1 | PLA2G10 | 0.742 | < 0.001 |
| AC007255.1 | HNF4A-AS1 | 0.741 | < 0.001 |
| AC007255.1 | AC004982.2 | 0.741 | < 0.001 |
| AC007255.1 | MYO15B | 0.741 | < 0.001 |
| AC007255.1 | FA2H | 0.741 | < 0.001 |
| AC007255.1 | PPARG | 0.740 | < 0.001 |
| AC007255.1 | LINC02038 | 0.739 | < 0.001 |
| AC007255.1 | FOXA3 | 0.738 | < 0.001 |
| AC007255.1 | LRRC31 | 0.738 | < 0.001 |
| AC007255.1 | P4HTM | 0.738 | < 0.001 |
| AC007255.1 | ACBD5 | 0.738 | < 0.001 |
| AC007255.1 | AC130456.4 | 0.737 | < 0.001 |
| AC007255.1 | RSPH1 | 0.737 | < 0.001 |
| AC007255.1 | CHDH | 0.737 | < 0.001 |
| AC007255.1 | MIR194-2HG | 0.737 | < 0.001 |
| AC007255.1 | B3GNT3 | 0.736 | < 0.001 |
| AC007255.1 | AC012317.1 | 0.736 | < 0.001 |
| AC007255.1 | TJP3 | 0.736 | < 0.001 |
| AC007255.1 | MYO1A | 0.736 | < 0.001 |
| AC007255.1 | TSPAN3 | 0.735 | < 0.001 |
| AC007255.1 | PDZD3 | 0.735 | < 0.001 |
| AC007255.1 | PLCH1 | 0.735 | < 0.001 |
| AC007255.1 | HNF1A | 0.735 | < 0.001 |
| AC007255.1 | HNF1A-AS1 | 0.734 | < 0.001 |
| AC007255.1 | LINC01978 | 0.733 | < 0.001 |
| AC007255.1 | APOBEC1 | 0.733 | < 0.001 |
| AC007255.1 | AL117382.2 | 0.733 | < 0.001 |
| AC007255.1 | IYD | 0.733 | < 0.001 |
| AC007255.1 | STAMBPL1 | 0.732 | < 0.001 |
| AC007255.1 | SLC37A1 | 0.732 | < 0.001 |
| AC007255.1 | GFPT1 | 0.732 | < 0.001 |
| AC007255.1 | TSPAN12 | 0.731 | < 0.001 |
| AC007255.1 | AC004830.2 | 0.731 | < 0.001 |
| AC007255.1 | MYRF-AS1 | 0.730 | < 0.001 |
| AC007255.1 | CDHR5 | 0.730 | < 0.001 |
| AC007255.1 | R3HDML | 0.730 | < 0.001 |
| AC007255.1 | PPP1R14D | 0.730 | < 0.001 |
| AC007255.1 | HGD | 0.729 | < 0.001 |
| AC007255.1 | AL606489.1 | 0.729 | < 0.001 |
| AC007255.1 | MMP15 | 0.728 | < 0.001 |
| AC007255.1 | MACC1 | 0.728 | < 0.001 |
| AC007255.1 | ERBB3 | 0.728 | < 0.001 |
| AC007255.1 | DTX4 | 0.728 | < 0.001 |
| AC007255.1 | CRYZL2P | 0.728 | < 0.001 |
| AC007255.1 | CLMN | 0.728 | < 0.001 |
| AC007255.1 | MYRF | 0.727 | < 0.001 |
| AC007255.1 | MNX1 | 0.727 | < 0.001 |
| AC007255.1 | GALM | 0.726 | < 0.001 |
| AC007255.1 | SLC26A8 | 0.726 | < 0.001 |
| AC007255.1 | SPIRE2 | 0.725 | < 0.001 |
| AC007255.1 | DDAH1 | 0.725 | < 0.001 |
| AC007255.1 | PRXL2B | 0.725 | < 0.001 |
| AC007255.1 | AGR2 | 0.725 | < 0.001 |
| AC007255.1 | RAB17 | 0.725 | < 0.001 |
| AC007255.1 | TMPRSS2 | 0.725 | < 0.001 |
| AC007255.1 | LINC00543 | 0.724 | < 0.001 |
| AC007255.1 | AC016735.1 | 0.724 | < 0.001 |
| AC007255.1 | NCMAP | 0.724 | < 0.001 |
| AC007255.1 | AVL9 | 0.723 | < 0.001 |
| AC007255.1 | MISP | 0.723 | < 0.001 |
| AC007255.1 | UNC5CL | 0.723 | < 0.001 |
| AC007255.1 | FZD5 | 0.722 | < 0.001 |
| AC007255.1 | GCNT3 | 0.722 | < 0.001 |
| AC007255.1 | HOTTIP | 0.722 | < 0.001 |
| AC007255.1 | AL117382.3 | 0.721 | < 0.001 |
| AC007255.1 | ENPP7P11 | 0.721 | < 0.001 |
| AC007255.1 | OSBPL7 | 0.721 | < 0.001 |
| AC007255.1 | SLC41A2 | 0.721 | < 0.001 |
| AC007255.1 | SLC44A3 | 0.721 | < 0.001 |
| AC007255.1 | RNF103 | 0.720 | < 0.001 |
| AC007255.1 | HNF4G | 0.719 | < 0.001 |
| AC007255.1 | GOLT1A | 0.719 | < 0.001 |
| AC007255.1 | PRR26 | 0.719 | < 0.001 |
| AC007255.1 | PDXDC1 | 0.719 | < 0.001 |
| AC007255.1 | RALGPS1 | 0.718 | < 0.001 |
| AC007255.1 | AC106876.1 | 0.718 | < 0.001 |
| AC007255.1 | GPR35 | 0.717 | < 0.001 |
| AC007255.1 | SLC44A4 | 0.717 | < 0.001 |
| AC007255.1 | AC097639.1 | 0.717 | < 0.001 |
| AC007255.1 | KRT8 | 0.717 | < 0.001 |
| AC007255.1 | BCL2L14 | 0.717 | < 0.001 |
| AC007255.1 | AC007009.1 | 0.715 | < 0.001 |
| AC007255.1 | TSPAN1 | 0.715 | < 0.001 |
| AC007255.1 | HNF4A | 0.714 | < 0.001 |
| AC007255.1 | CAPN8 | 0.714 | < 0.001 |
| AC007255.1 | LINC01979 | 0.714 | < 0.001 |
| AC007255.1 | AC020656.2 | 0.713 | < 0.001 |
| AC007255.1 | B3GNT7 | 0.713 | < 0.001 |
| AC007255.1 | FAM83E | 0.713 | < 0.001 |
| AC007255.1 | FMO5 | 0.712 | < 0.001 |
| AC007255.1 | ADCY6 | 0.712 | < 0.001 |
| AC007255.1 | SELENBP1 | 0.712 | < 0.001 |
| AC007255.1 | TMEM82 | 0.712 | < 0.001 |
| AC007255.1 | SH3BGRL2 | 0.711 | < 0.001 |
| AC007255.1 | AL034550.1 | 0.711 | < 0.001 |
| AC007255.1 | CALML4 | 0.711 | < 0.001 |
| AC007255.1 | LINC002481 | 0.710 | < 0.001 |
| AC007255.1 | LRRIQ4 | 0.710 | < 0.001 |
| AC007255.1 | LRRC75A | 0.710 | < 0.001 |
| AC007255.1 | AC004066.2 | 0.710 | < 0.001 |
| AC007255.1 | CHN2-AS1 | 0.710 | < 0.001 |
| AC007255.1 | LINC01594 | 0.709 | < 0.001 |
| AC007255.1 | AC005920.1 | 0.709 | < 0.001 |
| AC007255.1 | TSPAN8 | 0.709 | < 0.001 |
| AC007255.1 | COBL | 0.709 | < 0.001 |
| AC007255.1 | C2orf15 | 0.709 | < 0.001 |
| AC007255.1 | ARHGAP26 | 0.709 | < 0.001 |
| AC007255.1 | GLOD5 | 0.709 | < 0.001 |
| AC007255.1 | SIGIRR | 0.709 | < 0.001 |
| AC007255.1 | AL391832.2 | 0.708 | < 0.001 |
| AC007255.1 | CMTM8 | 0.708 | < 0.001 |
| AC007255.1 | MAP4K3-DT | 0.708 | < 0.001 |
| AC007255.1 | SLC25A25-AS1 | 0.708 | < 0.001 |
| AC007255.1 | RNF186 | 0.708 | < 0.001 |
| AC007255.1 | AC004982.1 | 0.708 | < 0.001 |
| AC007255.1 | CDX2 | 0.707 | < 0.001 |
| AC007255.1 | ACP6 | 0.707 | < 0.001 |
| AC007255.1 | PLEKHS1 | 0.707 | < 0.001 |
| AC007255.1 | BCAR3-AS1 | 0.707 | < 0.001 |
| AC007255.1 | PGPEP1 | 0.707 | < 0.001 |
| AC007255.1 | TMEM139 | 0.707 | < 0.001 |
| AC007255.1 | ARHGEF38-IT1 | 0.707 | < 0.001 |
| AC007255.1 | ANXA4 | 0.707 | < 0.001 |
| AC007255.1 | SMIM31 | 0.706 | < 0.001 |
| AC007255.1 | LINC02313 | 0.706 | < 0.001 |
| AC007255.1 | AC091588.3 | 0.706 | < 0.001 |
| AC007255.1 | AC091492.1 | 0.706 | < 0.001 |
| AC007255.1 | LINC01843 | 0.705 | < 0.001 |
| AC007255.1 | RASEF | 0.705 | < 0.001 |
| AC007255.1 | BCAS1 | 0.705 | < 0.001 |
| AC007255.1 | GCC2 | 0.705 | < 0.001 |
| AC007255.1 | GOLM1 | 0.705 | < 0.001 |
| AC007255.1 | TBC1D30 | 0.704 | < 0.001 |
| AC007255.1 | TSPAN15 | 0.704 | < 0.001 |
| AC007255.1 | C4orf19 | 0.704 | < 0.001 |
| AC007255.1 | HNF1B | 0.703 | < 0.001 |
| AC007255.1 | AC005037.1 | 0.703 | < 0.001 |
| AC007255.1 | AGR3 | 0.703 | < 0.001 |
| AC007255.1 | GSDMB | 0.703 | < 0.001 |
| AC007255.1 | BNIP5 | 0.702 | < 0.001 |
| AC007255.1 | KCNK5 | 0.702 | < 0.001 |
| AC007255.1 | PDX1 | 0.701 | < 0.001 |
| AC007255.1 | AC129507.3 | 0.701 | < 0.001 |
| AC007255.1 | CHKA | 0.701 | < 0.001 |
| AC007255.1 | PLEKHB1 | 0.701 | < 0.001 |
| AC007255.1 | FUCA1 | 0.700 | < 0.001 |
| AC007255.1 | CFTR | 0.700 | < 0.001 |
| AC007255.1 | AC099521.3 | 0.700 | < 0.001 |
| AC007255.1 | DNAJC22 | 0.700 | < 0.001 |
| AC007255.1 | KCNE3 | 0.699 | < 0.001 |
| AC007255.1 | RUNDC1 | 0.699 | < 0.001 |
| AC007255.1 | MGAT4A | 0.699 | < 0.001 |
| AC007255.1 | C4BPB | 0.699 | < 0.001 |
| AC007255.1 | RAB19 | 0.698 | < 0.001 |
| AC007255.1 | AC254629.1 | 0.698 | < 0.001 |
| AC007255.1 | AC004923.1 | 0.698 | < 0.001 |
| AC007255.1 | SMPDL3B | 0.698 | < 0.001 |
| AC007255.1 | AC090181.3 | 0.697 | < 0.001 |
| AC007255.1 | PRR13 | 0.697 | < 0.001 |
| AC007255.1 | ZNF620 | 0.697 | < 0.001 |
| AC007255.1 | ASL | 0.697 | < 0.001 |
| AC007255.1 | ANKRD40CL | 0.697 | < 0.001 |
| AC007255.1 | ANKS4B | 0.697 | < 0.001 |
| AC007255.1 | RAB11FIP4 | 0.696 | < 0.001 |
| AC007255.1 | TSPAN13 | 0.696 | < 0.001 |
| AC007255.1 | SPATA13 | 0.696 | < 0.001 |
| AC007255.1 | C1GALT1 | 0.696 | < 0.001 |
| AC007255.1 | SLC44A3-AS1 | 0.695 | < 0.001 |
| AC007255.1 | ANKMY2 | 0.694 | < 0.001 |
| AC007255.1 | PIWIL4 | 0.694 | < 0.001 |
| AC007255.1 | GCNT1 | 0.694 | < 0.001 |
| AC007255.1 | ADAP1 | 0.694 | < 0.001 |
| AC007255.1 | AL022322.1 | 0.694 | < 0.001 |
| AC007255.1 | SLC17A4 | 0.694 | < 0.001 |
| AC007255.1 | TM9SF3 | 0.694 | < 0.001 |
| AC007255.1 | RIPK3 | 0.694 | < 0.001 |
| AC007255.1 | PLEKHH1 | 0.694 | < 0.001 |
| AC007255.1 | AC012485.2 | 0.694 | < 0.001 |
| AC007255.1 | ENTPD8 | 0.693 | < 0.001 |
| AC007255.1 | PNKD | 0.693 | < 0.001 |
| AC007255.1 | MUC13 | 0.693 | < 0.001 |
| AC007255.1 | CYP3A5 | 0.693 | < 0.001 |
| AC007255.1 | SSTR1 | 0.693 | < 0.001 |
| AC007255.1 | AL450263.1 | 0.693 | < 0.001 |
| AC007255.1 | METTL7B | 0.693 | < 0.001 |
| AC007255.1 | C2orf72 | 0.692 | < 0.001 |
| AC007255.1 | OMP | 0.692 | < 0.001 |
| AC007255.1 | TTLL6 | 0.692 | < 0.001 |
| AC007255.1 | EPS8L3 | 0.692 | < 0.001 |
| AC007255.1 | RPS6KL1 | 0.692 | < 0.001 |
| AC007255.1 | SMIM24 | 0.692 | < 0.001 |
| AC007255.1 | SMIM22 | 0.692 | < 0.001 |
| AC007255.1 | EPHA10 | 0.692 | < 0.001 |
| AC007255.1 | HHLA2 | 0.692 | < 0.001 |
| AC007255.1 | PARD6B | 0.692 | < 0.001 |
| AC007255.1 | CYSTM1 | 0.692 | < 0.001 |
| AC007255.1 | TM4SF5 | 0.692 | < 0.001 |
| AC007255.1 | NOSTRIN | 0.691 | < 0.001 |
| AC007255.1 | MOGAT3 | 0.691 | < 0.001 |
| AC007255.1 | KRT18P15 | 0.691 | < 0.001 |
| AC007255.1 | GORASP1 | 0.691 | < 0.001 |
| AC007255.1 | RN7SL541P | 0.690 | < 0.001 |
| AC007255.1 | SLC39A5 | 0.690 | < 0.001 |
| AC007255.1 | CFAP65 | 0.690 | < 0.001 |
| AC007255.1 | AC116552.1 | 0.689 | < 0.001 |
| AC007255.1 | AC129507.2 | 0.689 | < 0.001 |
| AC007255.1 | AC092868.2 | 0.688 | < 0.001 |
| AC007255.1 | ELF3 | 0.688 | < 0.001 |
| AC007255.1 | CACFD1 | 0.688 | < 0.001 |
| AC007255.1 | SPACA4 | 0.688 | < 0.001 |
| AC007255.1 | FAM47E | 0.688 | < 0.001 |
| AC007255.1 | SULT1C2 | 0.686 | < 0.001 |
| AC007255.1 | ADCY6-DT | 0.686 | < 0.001 |
| AC007255.1 | ACSM3 | 0.686 | < 0.001 |
| AC007255.1 | CEMIP2 | 0.686 | < 0.001 |
| AC007255.1 | VILL | 0.686 | < 0.001 |
| AC007255.1 | RBM47 | 0.686 | < 0.001 |
| AC007255.1 | EVX1 | 0.685 | < 0.001 |
| AC007255.1 | C1orf115 | 0.685 | < 0.001 |
| AC007255.1 | AL121895.1 | 0.685 | < 0.001 |
| AC007255.1 | CA13 | 0.685 | < 0.001 |
| AC007255.1 | TTC38 | 0.685 | < 0.001 |
| AC007255.1 | SMIM6 | 0.685 | < 0.001 |
| AC007255.1 | SLC3A1 | 0.684 | < 0.001 |
| AC007255.1 | GAL3ST1 | 0.684 | < 0.001 |
| AC007255.1 | KAZALD1 | 0.684 | < 0.001 |
| AC007255.1 | AC008147.1 | 0.683 | < 0.001 |
| AC007255.1 | CAPN5 | 0.683 | < 0.001 |
| AC007255.1 | AC009065.3 | 0.683 | < 0.001 |
| AC007255.1 | VIL1 | 0.683 | < 0.001 |
| AC007255.1 | LINC00628 | 0.683 | < 0.001 |
| AC007255.1 | AC097493.2 | 0.683 | < 0.001 |
| AC007255.1 | FER1L4 | 0.683 | < 0.001 |
| AC007255.1 | ZSCAN16 | 0.683 | < 0.001 |
| AC007255.1 | DEPTOR | 0.683 | < 0.001 |
| AC007255.1 | AC114488.1 | 0.683 | < 0.001 |
| AC007255.1 | SMAD6 | 0.682 | < 0.001 |
| AC007255.1 | SLC6A20 | 0.682 | < 0.001 |
| AC007255.1 | EXOC6 | 0.682 | < 0.001 |
| AC007255.1 | PHACTR2-AS1 | 0.682 | < 0.001 |
| AC007255.1 | AL591895.1 | 0.682 | < 0.001 |
| AC007255.1 | MUC3A | 0.682 | < 0.001 |
| AC007255.1 | FAM81A | 0.681 | < 0.001 |
| AC007255.1 | AL359075.1 | 0.681 | < 0.001 |
| AC007255.1 | AC008040.2 | 0.681 | < 0.001 |
| AC007255.1 | NOL4L | 0.681 | < 0.001 |
| AC007255.1 | FMN1 | 0.681 | < 0.001 |
| AC007255.1 | BTNL8 | 0.681 | < 0.001 |
| AC007255.1 | LINC01091 | 0.681 | < 0.001 |
| AC007255.1 | SPDEF | 0.680 | < 0.001 |
| AC007255.1 | SGK2 | 0.680 | < 0.001 |
| AC007255.1 | SPATA6L | 0.680 | < 0.001 |
| AC007255.1 | LGALS4 | 0.680 | < 0.001 |
| AC007255.1 | AC090181.2 | 0.680 | < 0.001 |
| AC007255.1 | AC008521.1 | 0.680 | < 0.001 |
| AC007255.1 | IL17RB | 0.680 | < 0.001 |
| AC007255.1 | TRIM10 | 0.680 | < 0.001 |
| AC007255.1 | PPP1R1B | 0.680 | < 0.001 |
| AC007255.1 | ABCC6P1 | 0.680 | < 0.001 |
| AC007255.1 | MGAM2 | 0.679 | < 0.001 |
| AC007255.1 | SEMA4G | 0.679 | < 0.001 |
| AC007255.1 | POC1B | 0.679 | < 0.001 |
| AC007255.1 | ALDOB | 0.679 | < 0.001 |
| AC007255.1 | OCLN | 0.679 | < 0.001 |
| AC007255.1 | MS4A8 | 0.679 | < 0.001 |
| AC007255.1 | NANOGP4 | 0.679 | < 0.001 |
| AC007255.1 | AC127521.1 | 0.679 | < 0.001 |
| AC007255.1 | CCNI2 | 0.679 | < 0.001 |
| AC007255.1 | ACY3 | 0.679 | < 0.001 |
| AC007255.1 | AC073957.3 | 0.678 | < 0.001 |
| AC007255.1 | TRBV29OR9-2 | 0.678 | < 0.001 |
| AC007255.1 | KRTAP5-AS1 | 0.677 | < 0.001 |
| AC007255.1 | AC009065.6 | 0.677 | < 0.001 |
| AC007255.1 | GPRC5A | 0.677 | < 0.001 |
| AC007255.1 | SFMBT1 | 0.677 | < 0.001 |
| AC007255.1 | SLC29A2 | 0.677 | < 0.001 |
| AC007255.1 | KCNJ3 | 0.676 | < 0.001 |
| AC007255.1 | GPD1 | 0.676 | < 0.001 |
| AC007255.1 | ENPP4 | 0.676 | < 0.001 |
| AC007255.1 | AC079775.1 | 0.676 | < 0.001 |
| AC007255.1 | MIR3646 | 0.676 | < 0.001 |
| AC007255.1 | PTPRH | 0.675 | < 0.001 |
| AC007255.1 | AL078459.1 | 0.675 | < 0.001 |
| AC007255.1 | PAFAH2 | 0.675 | < 0.001 |
| AC007255.1 | HDHD3 | 0.675 | < 0.001 |
| AC007255.1 | CLDN23 | 0.675 | < 0.001 |
| AC007255.1 | MFSD9 | 0.675 | < 0.001 |
| AC007255.1 | REPS2 | 0.675 | < 0.001 |
| AC007255.1 | ABHD11 | 0.675 | < 0.001 |
| AC007255.1 | RAD17P1 | 0.675 | < 0.001 |
| AC007255.1 | TMPRSS3 | 0.674 | < 0.001 |
| AC007255.1 | NEK3 | 0.674 | < 0.001 |
| AC007255.1 | SYBU | 0.674 | < 0.001 |
| AC007255.1 | TRIM15 | 0.674 | < 0.001 |
| AC007255.1 | DDC | 0.674 | < 0.001 |
| AC007255.1 | ALPI | 0.674 | < 0.001 |
| AC007255.1 | ALG1L5P | 0.674 | < 0.001 |
| AC007255.1 | RHOU | 0.673 | < 0.001 |
| AC007255.1 | SOWAHA | 0.673 | < 0.001 |
| AC007255.1 | MIR548I2 | 0.673 | < 0.001 |
| AC007255.1 | STPG4 | 0.673 | < 0.001 |
| AC007255.1 | AC091588.1 | 0.673 | < 0.001 |
| AC007255.1 | BTNL3 | 0.673 | < 0.001 |
| AC007255.1 | DFFB | 0.673 | < 0.001 |
| AC007255.1 | CYP2B6 | 0.673 | < 0.001 |
| AC007255.1 | PTP4A2 | 0.673 | < 0.001 |
| AC007255.1 | DLG3 | 0.672 | < 0.001 |
| AC007255.1 | SAMD13 | 0.672 | < 0.001 |
| AC007255.1 | HSD17B11 | 0.672 | < 0.001 |
| AC007255.1 | AC124947.2 | 0.672 | < 0.001 |
| AC007255.1 | AC011773.3 | 0.672 | < 0.001 |
| AC007255.1 | AC007114.2 | 0.671 | < 0.001 |
| AC007255.1 | AC091043.1 | 0.671 | < 0.001 |
| AC007255.1 | PKP2 | 0.670 | < 0.001 |
| AC007255.1 | C1orf210 | 0.670 | < 0.001 |
| AC007255.1 | TMEM144 | 0.670 | < 0.001 |
| AC007255.1 | AGMAT | 0.670 | < 0.001 |
| AC007255.1 | MYO7B | 0.669 | < 0.001 |
| AC007255.1 | TMEM53 | 0.669 | < 0.001 |
| AC007255.1 | ABHD11-AS1 | 0.669 | < 0.001 |
| AC007255.1 | AC098476.1 | 0.668 | < 0.001 |
| AC007255.1 | AL133410.1 | 0.668 | < 0.001 |
| AC007255.1 | PRAP1 | 0.668 | < 0.001 |
| AC007255.1 | KRT18 | 0.668 | < 0.001 |
| AC007255.1 | AC021218.1 | 0.668 | < 0.001 |
| AC007255.1 | GALNT4 | 0.668 | < 0.001 |
| AC007255.1 | TNFRSF14-AS1 | 0.668 | < 0.001 |
| AC007255.1 | AC025580.2 | 0.668 | < 0.001 |
| AC007255.1 | FUT4 | 0.667 | < 0.001 |
| AC007255.1 | LPIN2 | 0.667 | < 0.001 |
| AC007255.1 | GATA6 | 0.667 | < 0.001 |
| AC007255.1 | GDPD1 | 0.667 | < 0.001 |
| AC007255.1 | SULT1C2P1 | 0.667 | < 0.001 |
| AC007255.1 | GIPC2 | 0.667 | < 0.001 |
| AC007255.1 | AC244100.3 | 0.666 | < 0.001 |
| AC007255.1 | AC005062.1 | 0.666 | < 0.001 |
| AC007255.1 | LINC00501 | 0.666 | < 0.001 |
| AC007255.1 | TRAK1 | 0.666 | < 0.001 |
| AC007255.1 | AL035701.1 | 0.666 | < 0.001 |
| AC007255.1 | FBP1 | 0.665 | < 0.001 |
| AC007255.1 | AC025154.2 | 0.665 | < 0.001 |
| AC007255.1 | SUCLG2 | 0.665 | < 0.001 |
| AC007255.1 | TRIM40 | 0.665 | < 0.001 |
| AC007255.1 | RALGAPA2 | 0.665 | < 0.001 |
| AC007255.1 | EML4 | 0.664 | < 0.001 |
| AC007255.1 | ZNF69 | 0.664 | < 0.001 |
| AC007255.1 | AC004080.2 | 0.664 | < 0.001 |
| AC007255.1 | RAB20 | 0.664 | < 0.001 |
| AC007255.1 | AC104667.2 | 0.664 | < 0.001 |
| AC007255.1 | CA1 | 0.664 | < 0.001 |
| AC007255.1 | LRRC66 | 0.664 | < 0.001 |
| AC007255.1 | AL162727.2 | 0.664 | < 0.001 |
| AC007255.1 | AC124067.4 | 0.664 | < 0.001 |
| AC007255.1 | SHROOM3 | 0.664 | < 0.001 |
| AC007255.1 | GDA | 0.663 | < 0.001 |
| AC007255.1 | KRTAP5-1 | 0.663 | < 0.001 |
| AC007255.1 | ADGRG7 | 0.663 | < 0.001 |
| AC007255.1 | FAM102B | 0.663 | < 0.001 |
| AC007255.1 | TDRKH-AS1 | 0.663 | < 0.001 |
| AC007255.1 | CLRN3 | 0.663 | < 0.001 |
| AC007255.1 | AP004608.1 | 0.663 | < 0.001 |
| AC007255.1 | RNFT1P2 | 0.662 | < 0.001 |
| AC007255.1 | TESC | 0.662 | < 0.001 |
| AC007255.1 | SLC12A2 | 0.662 | < 0.001 |
| AC007255.1 | ARHGAP18 | 0.662 | < 0.001 |
| AC007255.1 | LINC01558 | 0.662 | < 0.001 |
| AC007255.1 | AC004923.4 | 0.662 | < 0.001 |
| AC007255.1 | FRMD5 | 0.662 | < 0.001 |
| AC007255.1 | LBX2-AS1 | 0.662 | < 0.001 |
| AC007255.1 | BX255925.1 | 0.662 | < 0.001 |
| AC007255.1 | ZNF888 | 0.662 | < 0.001 |
| AC007255.1 | SEL1L3 | 0.661 | < 0.001 |
| AC007255.1 | DCDC2 | 0.661 | < 0.001 |
| AC007255.1 | KRT8P3 | 0.660 | < 0.001 |
| AC007255.1 | CDR2 | 0.660 | < 0.001 |
| AC007255.1 | GJB1 | 0.660 | < 0.001 |
| AC007255.1 | NAT1 | 0.659 | < 0.001 |
| AC007255.1 | AL596223.2 | 0.659 | < 0.001 |
| AC007255.1 | XK | 0.659 | < 0.001 |
| AC007255.1 | SMPD3 | 0.659 | < 0.001 |
| AC007255.1 | KIF9 | 0.659 | < 0.001 |
| AC007255.1 | SLC10A5 | 0.659 | < 0.001 |
| AC007255.1 | ZNF321P | 0.659 | < 0.001 |
| AC007255.1 | NAT2 | 0.659 | < 0.001 |
| AC007255.1 | HSH2D | 0.659 | < 0.001 |
| AC007255.1 | MYO6 | 0.659 | < 0.001 |
| AC007255.1 | RNF183 | 0.658 | < 0.001 |
| AC007255.1 | MUC17 | 0.658 | < 0.001 |
| AC007255.1 | GALNT12 | 0.657 | < 0.001 |
| AC007255.1 | ACSL5 | 0.657 | < 0.001 |
| AC007255.1 | ATP7B | 0.657 | < 0.001 |
| AC007255.1 | SPATA3-AS1 | 0.657 | < 0.001 |
| AC007255.1 | CAPN10 | 0.657 | < 0.001 |
| AC007255.1 | TAAR3P | 0.656 | < 0.001 |
| AC007255.1 | AL133215.1 | 0.656 | < 0.001 |
| AC007255.1 | AL157786.1 | 0.656 | < 0.001 |
| AC007255.1 | TMEM238L | 0.656 | < 0.001 |
| AC007255.1 | AC020663.2 | 0.656 | < 0.001 |
| AC007255.1 | PHETA1 | 0.656 | < 0.001 |
| AC007255.1 | GLB1 | 0.656 | < 0.001 |
| AC007255.1 | PTPRJ | 0.655 | < 0.001 |
| AC007255.1 | ARRB1 | 0.655 | < 0.001 |
| AC007255.1 | AP000866.2 | 0.655 | < 0.001 |
| AC007255.1 | NR5A2 | 0.654 | < 0.001 |
| AC007255.1 | AP001625.2 | 0.654 | < 0.001 |
| AC007255.1 | DOK4 | 0.654 | < 0.001 |
| AC007255.1 | AP006621.1 | 0.654 | < 0.001 |
| AC007255.1 | CNNM3 | 0.654 | < 0.001 |
| AC007255.1 | AC093110.1 | 0.654 | < 0.001 |
| AC007255.1 | ENPP7P7 | 0.653 | < 0.001 |
| AC007255.1 | AC105935.1 | 0.653 | < 0.001 |
| AC007255.1 | KCNQ1 | 0.653 | < 0.001 |
| AC007255.1 | AC130456.1 | 0.653 | < 0.001 |
| AC007255.1 | UGT2B7 | 0.653 | < 0.001 |
| AC007255.1 | AC073283.1 | 0.653 | < 0.001 |
| AC007255.1 | FOXD2 | 0.653 | < 0.001 |
| AC007255.1 | AC005550.2 | 0.653 | < 0.001 |
| AC007255.1 | RNU4-38P | 0.653 | < 0.001 |
| AC007255.1 | RXFP4 | 0.653 | < 0.001 |
| AC007255.1 | ENPP7P14 | 0.652 | < 0.001 |
| AC007255.1 | PRKAB1 | 0.652 | < 0.001 |
| AC007255.1 | SEC23IP | 0.652 | < 0.001 |
| AC007255.1 | NECTIN2 | 0.652 | < 0.001 |
| AC007255.1 | BCO1 | 0.652 | < 0.001 |
| AC007255.1 | ALDH3B1 | 0.652 | < 0.001 |
| AC007255.1 | GLYCTK | 0.651 | < 0.001 |
| AC007255.1 | PTPN18 | 0.651 | < 0.001 |
| AC007255.1 | TSEN2 | 0.651 | < 0.001 |
| AC007255.1 | CAPN9 | 0.651 | < 0.001 |
| AC007255.1 | AL021331.1 | 0.651 | < 0.001 |
| AC007255.1 | AC004009.2 | 0.651 | < 0.001 |
| AC007255.1 | RPL6P7 | 0.651 | < 0.001 |
| AC007255.1 | ANG | 0.651 | < 0.001 |
| AC007255.1 | NEK5 | 0.651 | < 0.001 |
| AC007255.1 | CHPT1 | 0.651 | < 0.001 |
| AC007255.1 | SLC45A3 | 0.651 | < 0.001 |
| AC007255.1 | MYB | 0.650 | < 0.001 |
| AC007255.1 | TBC1D12 | 0.650 | < 0.001 |
| AC007255.1 | DNAH6 | 0.650 | < 0.001 |
| AC007255.1 | ASRGL1 | 0.649 | < 0.001 |
| AC007255.1 | ORMDL3 | 0.649 | < 0.001 |
| AC007255.1 | AC009022.1 | 0.649 | < 0.001 |
| AC007255.1 | EPS8 | 0.649 | < 0.001 |
| AC007255.1 | KISS1 | 0.649 | < 0.001 |
| AC007255.1 | DGKD | 0.648 | < 0.001 |
| AC007255.1 | TCF7L2 | 0.648 | < 0.001 |
| AC007255.1 | TMEM51-AS1 | 0.648 | < 0.001 |
| AC007255.1 | AC093840.1 | 0.648 | < 0.001 |
| AC007255.1 | FNBP1L | 0.648 | < 0.001 |
| AC007255.1 | ZNF619 | 0.648 | < 0.001 |
| AC007255.1 | AC011676.5 | 0.648 | < 0.001 |
| AC007255.1 | IQCE | 0.647 | < 0.001 |
| AC007255.1 | MIR3131 | 0.647 | < 0.001 |
| AC007255.1 | MUC1 | 0.647 | < 0.001 |
| AC007255.1 | TRIM80P | 0.647 | < 0.001 |
| AC007255.1 | AL139246.3 | 0.647 | < 0.001 |
| AC007255.1 | TNNC1 | 0.647 | < 0.001 |
| AC007255.1 | HMGCS2 | 0.646 | < 0.001 |
| AC007255.1 | IRF8 | 0.646 | < 0.001 |
| AC007255.1 | AC091563.1 | 0.646 | < 0.001 |
| AC007255.1 | PROM1 | 0.646 | < 0.001 |
| AC007255.1 | C1GALT1C1 | 0.646 | < 0.001 |
| AC007255.1 | GPA33 | 0.646 | < 0.001 |
| AC007255.1 | SLC35D2 | 0.646 | < 0.001 |
| AC007255.1 | FAAH | 0.645 | < 0.001 |
| AC007255.1 | AC025580.1 | 0.645 | < 0.001 |
| AC007255.1 | KRT8P37 | 0.645 | < 0.001 |
| AC007255.1 | TMEM45B | 0.645 | < 0.001 |
| AC007255.1 | KIF3B | 0.645 | < 0.001 |
| AC007255.1 | CACNA1D | 0.645 | < 0.001 |
| AC007255.1 | KRT8P10 | 0.644 | < 0.001 |
| AC007255.1 | MNX1-AS2 | 0.644 | < 0.001 |
| AC007255.1 | LIPH | 0.644 | < 0.001 |
| AC007255.1 | CASC4 | 0.644 | < 0.001 |
| AC007255.1 | SLC17A5 | 0.644 | < 0.001 |
| AC007255.1 | AC004009.1 | 0.643 | < 0.001 |
| AC007255.1 | NR1I2 | 0.643 | < 0.001 |
| AC007255.1 | DEGS2 | 0.643 | < 0.001 |
| AC007255.1 | AC127526.1 | 0.643 | < 0.001 |
| AC007255.1 | TFF1 | 0.643 | < 0.001 |
| AC007255.1 | BMS1P1 | 0.643 | < 0.001 |
| AC007255.1 | FAHD2P1 | 0.643 | < 0.001 |
| AC007255.1 | AP000866.5 | 0.643 | < 0.001 |
| AC007255.1 | TNFSF15 | 0.643 | < 0.001 |
| AC007255.1 | VSIG2 | 0.643 | < 0.001 |
| AC007255.1 | CIDEC | 0.643 | < 0.001 |
| AC007255.1 | SVIP | 0.643 | < 0.001 |
| AC007255.1 | ELF3-AS1 | 0.643 | < 0.001 |
| AC007255.1 | AL390719.2 | 0.643 | < 0.001 |
| AC007255.1 | CLEC19A | 0.642 | < 0.001 |
| AC007255.1 | RNASE4 | 0.642 | < 0.001 |
| AC007255.1 | KRT20 | 0.642 | < 0.001 |
| AC007255.1 | PPP1R36 | 0.642 | < 0.001 |
| AC007255.1 | RETREG1 | 0.642 | < 0.001 |
| AC007255.1 | SIAE | 0.642 | < 0.001 |
| AC007255.1 | CD2AP | 0.642 | < 0.001 |
| AC007255.1 | RNASET2 | 0.642 | < 0.001 |
| AC007255.1 | KDELR2 | 0.641 | < 0.001 |
| AC007255.1 | ZNF239 | 0.641 | < 0.001 |
| AC007255.1 | ENC1 | 0.641 | < 0.001 |
| AC007255.1 | PIK3C2B | 0.641 | < 0.001 |
| AC007255.1 | SLC46A3 | 0.641 | < 0.001 |
| AC007255.1 | AP003478.1 | 0.641 | < 0.001 |
| AC007255.1 | SLC1A1 | 0.641 | < 0.001 |
| AC007255.1 | AL135999.3 | 0.641 | < 0.001 |
| AC007255.1 | HABP2 | 0.641 | < 0.001 |
| AC007255.1 | CXCL16 | 0.641 | < 0.001 |
| AC007255.1 | LINC00857 | 0.641 | < 0.001 |
| AC007255.1 | AC097468.1 | 0.641 | < 0.001 |
| AC007255.1 | MARCHF3 | 0.640 | < 0.001 |
| AC007255.1 | CRACD | 0.640 | < 0.001 |
| AC007255.1 | TGOLN2 | 0.640 | < 0.001 |
| AC007255.1 | ZC3H12B | 0.640 | < 0.001 |
| AC007255.1 | PF4 | 0.640 | < 0.001 |
| AC007255.1 | SLC35A3 | 0.640 | < 0.001 |
| AC007255.1 | MST1R | 0.640 | < 0.001 |
| AC007255.1 | LACTB2 | 0.640 | < 0.001 |
| AC007255.1 | FUT8 | 0.639 | < 0.001 |
| AC007255.1 | TNFSF13 | 0.639 | < 0.001 |
| AC007255.1 | AL590004.3 | 0.639 | < 0.001 |
| AC007255.1 | LCN12 | 0.639 | < 0.001 |
| AC007255.1 | FABP2 | 0.639 | < 0.001 |
| AC007255.1 | SLC26A1 | 0.639 | < 0.001 |
| AC007255.1 | PRR13P5 | 0.639 | < 0.001 |
| AC007255.1 | HOXB-AS3 | 0.639 | < 0.001 |
| AC007255.1 | AC008870.4 | 0.638 | < 0.001 |
| AC007255.1 | DPY19L1P1 | 0.638 | < 0.001 |
| AC007255.1 | KRT18P28 | 0.638 | < 0.001 |
| AC007255.1 | IGHV3-79 | 0.638 | < 0.001 |
| AC007255.1 | AL445686.1 | 0.638 | < 0.001 |
| AC007255.1 | AC022613.1 | 0.638 | < 0.001 |
| AC007255.1 | LASP1 | 0.638 | < 0.001 |
| AC007255.1 | DENND5B | 0.638 | < 0.001 |
| AC007255.1 | AQP11 | 0.638 | < 0.001 |
| AC007255.1 | ZNF774 | 0.637 | < 0.001 |
| AC007255.1 | AL365436.3 | 0.637 | < 0.001 |
| AC007255.1 | APAF1 | 0.637 | < 0.001 |
| AC007255.1 | AL731571.1 | 0.637 | < 0.001 |
| AC007255.1 | CARMIL1 | 0.637 | < 0.001 |
| AC007255.1 | CREB3L1 | 0.637 | < 0.001 |
| AC007255.1 | PLAAT3 | 0.637 | < 0.001 |
| AC007255.1 | SPTBN1 | 0.637 | < 0.001 |
| AC007255.1 | PRSS33 | 0.637 | < 0.001 |
| AC007255.1 | AMN | 0.636 | < 0.001 |
| AC007255.1 | DLG3-AS1 | 0.636 | < 0.001 |
| AC007255.1 | HKDC1 | 0.636 | < 0.001 |
| AC007255.1 | CDH17 | 0.636 | < 0.001 |
| AC007255.1 | AKAP1 | 0.636 | < 0.001 |
| AC007255.1 | AC104472.1 | 0.636 | < 0.001 |
| AC007255.1 | AC004233.2 | 0.635 | < 0.001 |
| AC007255.1 | LTK | 0.635 | < 0.001 |
| AC007255.1 | AC098869.2 | 0.635 | < 0.001 |
| AC007255.1 | ETNK1 | 0.635 | < 0.001 |
| AC007255.1 | CLDN2 | 0.635 | < 0.001 |
| AC007255.1 | LFNG | 0.635 | < 0.001 |
| AC007255.1 | AC020906.1 | 0.635 | < 0.001 |
| AC007255.1 | TM4SF20 | 0.635 | < 0.001 |
| AC007255.1 | MIR1293 | 0.635 | < 0.001 |
| AC007255.1 | BACE2 | 0.635 | < 0.001 |
| AC007255.1 | KIF12 | 0.635 | < 0.001 |
| AC007255.1 | ACSS2 | 0.635 | < 0.001 |
| AC007255.1 | AC027449.1 | 0.634 | < 0.001 |
| AC007255.1 | HPDL | 0.634 | < 0.001 |
| AC007255.1 | TMF1 | 0.634 | < 0.001 |
| AC007255.1 | EEPD1 | 0.634 | < 0.001 |
| AC007255.1 | RNU6-1281P | 0.634 | < 0.001 |
| AC007255.1 | SERPINA4 | 0.634 | < 0.001 |
| AC007255.1 | A1CF | 0.633 | < 0.001 |
| AC007255.1 | SETD6P1 | 0.633 | < 0.001 |
| AC007255.1 | FUT8-AS1 | 0.633 | < 0.001 |
| AC007255.1 | PIGR | 0.633 | < 0.001 |
| AC007255.1 | AQP7 | 0.633 | < 0.001 |
| AC007255.1 | SLC5A2 | 0.633 | < 0.001 |
| AC007255.1 | ADGRE5 | 0.633 | < 0.001 |
| AC007255.1 | PARD6A | 0.633 | < 0.001 |
| AC007255.1 | AC012467.1 | 0.632 | < 0.001 |
| AC007255.1 | TM4SF4 | 0.632 | < 0.001 |
| AC007255.1 | GPRC5C | 0.632 | < 0.001 |
| AC007255.1 | HOXB6 | 0.632 | < 0.001 |
| AC007255.1 | PDZD8 | 0.632 | < 0.001 |
| AC007255.1 | MGAT3-AS1 | 0.632 | < 0.001 |
| AC007255.1 | B3GALT5 | 0.632 | < 0.001 |
| AC007255.1 | CMPK1 | 0.632 | < 0.001 |
| AC007255.1 | PLCE1 | 0.631 | < 0.001 |
| AC007255.1 | ZDHHC6 | 0.631 | < 0.001 |
| AC007255.1 | KRT8P33 | 0.631 | < 0.001 |
| AC007255.1 | AL691482.4 | 0.631 | < 0.001 |
| AC007255.1 | LACTB2-AS1 | 0.631 | < 0.001 |
| AC007255.1 | S100P | 0.631 | < 0.001 |
| AC007255.1 | GATA6-AS1 | 0.631 | < 0.001 |
| AC007255.1 | EFNA2 | 0.631 | < 0.001 |
| AC007255.1 | VSIG10 | 0.631 | < 0.001 |
| AC007255.1 | ABCG8 | 0.631 | < 0.001 |
| AC007255.1 | HNMT | 0.631 | < 0.001 |
| AC007255.1 | FGFR4 | 0.630 | < 0.001 |
| AC007255.1 | HOXA13 | 0.630 | < 0.001 |
| AC007255.1 | GDF15 | 0.630 | < 0.001 |
| AC007255.1 | HOXB5 | 0.630 | < 0.001 |
| AC007255.1 | KRT7-AS | 0.629 | < 0.001 |
| AC007255.1 | LINC02688 | 0.629 | < 0.001 |
| AC007255.1 | AC026462.3 | 0.629 | < 0.001 |
| AC007255.1 | AL445523.1 | 0.629 | < 0.001 |
| AC007255.1 | MLXIPL | 0.629 | < 0.001 |
| AC007255.1 | NEU4 | 0.629 | < 0.001 |
| AC007255.1 | KRTAP5-5 | 0.629 | < 0.001 |
| AC007255.1 | OXNAD1 | 0.629 | < 0.001 |
| AC007255.1 | AL391832.1 | 0.628 | < 0.001 |
| AC007255.1 | MLEC | 0.628 | < 0.001 |
| AC007255.1 | CD164 | 0.628 | < 0.001 |
| AC007255.1 | NR0B2 | 0.628 | < 0.001 |
| AC007255.1 | MMEL1 | 0.628 | < 0.001 |
| AC007255.1 | C11orf53 | 0.627 | < 0.001 |
| AC007255.1 | MTMR11 | 0.627 | < 0.001 |
| AC007255.1 | HID1-AS1 | 0.627 | < 0.001 |
| AC007255.1 | AL606489.2 | 0.627 | < 0.001 |
| AC007255.1 | 2-Mar | 0.627 | < 0.001 |
| AC007255.1 | SERPINB6 | 0.627 | < 0.001 |
| AC007255.1 | AC093323.2 | 0.626 | < 0.001 |
| AC007255.1 | AC093720.1 | 0.626 | < 0.001 |
| AC007255.1 | VDAC1P8 | 0.626 | < 0.001 |
| AC007255.1 | SULT1A2 | 0.626 | < 0.001 |
| AC007255.1 | PDE4C | 0.626 | < 0.001 |
| AC007255.1 | TMC4 | 0.626 | < 0.001 |
| AC007255.1 | MCU | 0.626 | < 0.001 |
| AC007255.1 | MIR378G | 0.626 | < 0.001 |
| AC007255.1 | ST6GALNAC1 | 0.626 | < 0.001 |
| AC007255.1 | EDEM3 | 0.626 | < 0.001 |
| AC007255.1 | ATP8A1 | 0.625 | < 0.001 |
| AC007255.1 | NCR3LG1 | 0.625 | < 0.001 |
| AC007255.1 | RFK | 0.625 | < 0.001 |
| AC007255.1 | TFF3 | 0.625 | < 0.001 |
| AC007255.1 | AL121839.2 | 0.625 | < 0.001 |
| AC007255.1 | ACBD4 | 0.625 | < 0.001 |
| AC007255.1 | MROCKI | 0.625 | < 0.001 |
| AC007255.1 | DMBT1 | 0.625 | < 0.001 |
| AC007255.1 | AP005717.1 | 0.625 | < 0.001 |
| AC007255.1 | AC093732.1 | 0.625 | < 0.001 |
| AC007255.1 | AL354794.2 | 0.624 | < 0.001 |
| AC007255.1 | PTPRR | 0.624 | < 0.001 |
| AC007255.1 | CLDN4 | 0.624 | < 0.001 |
| AC007255.1 | LINC02435 | 0.624 | < 0.001 |
| AC007255.1 | AP001107.9 | 0.624 | < 0.001 |
| AC007255.1 | IGHVII-78-1 | 0.623 | < 0.001 |
| AC007255.1 | CLIC5 | 0.623 | < 0.001 |
| AC007255.1 | AC124798.1 | 0.623 | < 0.001 |
| AC007255.1 | LINC01915 | 0.623 | < 0.001 |
| AC007255.1 | CA3 | 0.623 | < 0.001 |
| AC007255.1 | CLDN7 | 0.623 | < 0.001 |
| AC007255.1 | CDHR2 | 0.623 | < 0.001 |
| AC007255.1 | NGLY1 | 0.623 | < 0.001 |
| AC007255.1 | ANXA13 | 0.623 | < 0.001 |
| AC007255.1 | MSH5-SAPCD1 | 0.623 | < 0.001 |
| AC007255.1 | TC2N | 0.622 | < 0.001 |
| AC007255.1 | SPINK4 | 0.622 | < 0.001 |
| AC007255.1 | GATM | 0.622 | < 0.001 |
| AC007255.1 | FCSK | 0.622 | < 0.001 |
| AC007255.1 | MIR3189 | 0.622 | < 0.001 |
| AC007255.1 | GALNT7 | 0.622 | < 0.001 |
| AC007255.1 | ASS1 | 0.622 | < 0.001 |
| AC007255.1 | CCL15-CCL14 | 0.622 | < 0.001 |
| AC007255.1 | SNORC | 0.622 | < 0.001 |
| AC007255.1 | LINC02754 | 0.621 | < 0.001 |
| AC007255.1 | RGL3 | 0.621 | < 0.001 |
| AC007255.1 | GUCY2C | 0.621 | < 0.001 |
| AC007255.1 | AL031320.2 | 0.621 | < 0.001 |
| AC007255.1 | BEND7 | 0.621 | < 0.001 |
| AC007255.1 | AOC1 | 0.621 | < 0.001 |
| AC007255.1 | SLC25A20 | 0.621 | < 0.001 |
| AC007255.1 | RRBP1 | 0.621 | < 0.001 |
| AC007255.1 | HES6 | 0.621 | < 0.001 |
| AC007255.1 | PGAP6 | 0.621 | < 0.001 |
| AC007255.1 | CASP10 | 0.621 | < 0.001 |
| AC007255.1 | SNRPCP5 | 0.621 | < 0.001 |
| AC007255.1 | GMDS | 0.620 | < 0.001 |
| AC007255.1 | KIF13B | 0.620 | < 0.001 |
| AC007255.1 | EPCAM-DT | 0.620 | < 0.001 |
| AC007255.1 | HSD17B2 | 0.620 | < 0.001 |
| AC007255.1 | ACRBP | 0.620 | < 0.001 |
| AC007255.1 | SLC40A1 | 0.620 | < 0.001 |
| AC007255.1 | TCAIM | 0.620 | < 0.001 |
| AC007255.1 | CHMP2B | 0.620 | < 0.001 |
| AC007255.1 | RWDD2A | 0.619 | < 0.001 |
| AC007255.1 | AL117187.1 | 0.619 | < 0.001 |
| AC007255.1 | AL133373.2 | 0.619 | < 0.001 |
| AC007255.1 | MSH5 | 0.619 | < 0.001 |
| AC007255.1 | C6orf99 | 0.619 | < 0.001 |
| AC007255.1 | ONECUT2 | 0.619 | < 0.001 |
| AC007255.1 | PKDCC | 0.619 | < 0.001 |
| AC007255.1 | ZG16B | 0.619 | < 0.001 |
| AC007255.1 | PRSS30P | 0.619 | < 0.001 |
| AC007255.1 | SYNPR | 0.618 | < 0.001 |
| AC007255.1 | AP000943.3 | 0.618 | < 0.001 |
| AC007255.1 | KRT8P17 | 0.618 | < 0.001 |
| AC007255.1 | PDE8A | 0.618 | < 0.001 |
| AC007255.1 | KRT18P11 | 0.618 | < 0.001 |
| AC007255.1 | KCNJ11 | 0.617 | < 0.001 |
| AC007255.1 | KRT8P45 | 0.617 | < 0.001 |
| AC007255.1 | CRYZL2P-SEC16B | 0.617 | < 0.001 |
| AC007255.1 | KBTBD12 | 0.617 | < 0.001 |
| AC007255.1 | INSL3 | 0.617 | < 0.001 |
| AC007255.1 | ADD3-AS1 | 0.617 | < 0.001 |
| AC007255.1 | FAM174B | 0.617 | < 0.001 |
| AC007255.1 | AP000919.1 | 0.617 | < 0.001 |
| AC007255.1 | FOXP4 | 0.617 | < 0.001 |
| AC007255.1 | AC007950.1 | 0.617 | < 0.001 |
| AC007255.1 | KRT8P32 | 0.617 | < 0.001 |
| AC007255.1 | LINC02489 | 0.616 | < 0.001 |
| AC007255.1 | GCA | 0.616 | < 0.001 |
| AC007255.1 | CMC1 | 0.616 | < 0.001 |
| AC007255.1 | MKLN1-AS | 0.615 | < 0.001 |
| AC007255.1 | SULT1C2P2 | 0.615 | < 0.001 |
| AC007255.1 | CRYL1 | 0.615 | < 0.001 |
| AC007255.1 | AC026471.3 | 0.615 | < 0.001 |
| AC007255.1 | CDC14B | 0.615 | < 0.001 |
| AC007255.1 | MTND4P23 | 0.615 | < 0.001 |
| AC007255.1 | GDPGP1 | 0.615 | < 0.001 |
| AC007255.1 | ARL14 | 0.615 | < 0.001 |
| AC007255.1 | IHH | 0.615 | < 0.001 |
| AC007255.1 | AC005089.1 | 0.615 | < 0.001 |
| AC007255.1 | SPINK1 | 0.614 | < 0.001 |
| AC007255.1 | HOXA11-AS | 0.614 | < 0.001 |
| AC007255.1 | TTLL2 | 0.614 | < 0.001 |
| AC007255.1 | AC009133.1 | 0.614 | < 0.001 |
| AC007255.1 | AC007637.1 | 0.614 | < 0.001 |
| AC007255.1 | MECOM | 0.614 | < 0.001 |
| AC007255.1 | KRT8P30 | 0.614 | < 0.001 |
| AC007255.1 | FAM118B | 0.614 | < 0.001 |
| AC007255.1 | SEMG1 | 0.614 | < 0.001 |
| AC007255.1 | OARD1 | 0.613 | < 0.001 |
| AC007255.1 | ARSL | 0.613 | < 0.001 |
| AC007255.1 | MIR608 | 0.613 | < 0.001 |
| AC007255.1 | KRT18P10 | 0.613 | < 0.001 |
| AC007255.1 | SULT1C3 | 0.613 | < 0.001 |
| AC007255.1 | AP4B1 | 0.613 | < 0.001 |
| AC007255.1 | LINC01389 | 0.613 | < 0.001 |
| AC007255.1 | TRPM5 | 0.613 | < 0.001 |
| AC007255.1 | AL355076.3 | 0.612 | < 0.001 |
| AC007255.1 | PRSS3 | 0.612 | < 0.001 |
| AC007255.1 | TSPO2 | 0.612 | < 0.001 |
| AC007255.1 | ABCC3 | 0.612 | < 0.001 |
| AC007255.1 | MKRN2OS | 0.612 | < 0.001 |
| AC007255.1 | CTBP2 | 0.612 | < 0.001 |
| AC007255.1 | TP53I11 | 0.612 | < 0.001 |
| AC007255.1 | ATP8B1 | 0.612 | < 0.001 |
| AC007255.1 | CYB5RL | 0.612 | < 0.001 |
| AC007255.1 | AC009407.1 | 0.612 | < 0.001 |
| AC007255.1 | CABLES1 | 0.612 | < 0.001 |
| AC007255.1 | HNRNPA1P17 | 0.612 | < 0.001 |
| AC007255.1 | DISP1 | 0.611 | < 0.001 |
| AC007255.1 | AL590627.1 | 0.611 | < 0.001 |
| AC007255.1 | STK39 | 0.611 | < 0.001 |
| AC007255.1 | ABCG5 | 0.611 | < 0.001 |
| AC007255.1 | MISP3 | 0.611 | < 0.001 |
| AC007255.1 | ASPHD1 | 0.611 | < 0.001 |
| AC007255.1 | CREB3L3 | 0.610 | < 0.001 |
| AC007255.1 | PTP4A2P1 | 0.610 | < 0.001 |
| AC007255.1 | AC094019.2 | 0.610 | < 0.001 |
| AC007255.1 | TPTE2P5 | 0.610 | < 0.001 |
| AC007255.1 | AC097493.1 | 0.610 | < 0.001 |
| AC007255.1 | CCRL2 | 0.610 | < 0.001 |
| AC007255.1 | ARHGEF2-AS1 | 0.610 | < 0.001 |
| AC007255.1 | TNFRSF11A | 0.610 | < 0.001 |
| AC007255.1 | GHDC | 0.609 | < 0.001 |
| AC007255.1 | TMED4 | 0.609 | < 0.001 |
| AC007255.1 | DOK1 | 0.609 | < 0.001 |
| AC007255.1 | PMEL | 0.609 | < 0.001 |
| AC007255.1 | PLS1-AS1 | 0.609 | < 0.001 |
| AC007255.1 | LZTS3 | 0.609 | < 0.001 |
| AC007255.1 | RASSF7 | 0.608 | < 0.001 |
| AC007255.1 | LGR4 | 0.608 | < 0.001 |
| AC007255.1 | TIGD4 | 0.608 | < 0.001 |
| AC007255.1 | AC000061.1 | 0.608 | < 0.001 |
| AC007255.1 | SPNS3 | 0.608 | < 0.001 |
| AC007255.1 | TEX45 | 0.608 | < 0.001 |
| AC007255.1 | C2CD4B | 0.608 | < 0.001 |
| AC007255.1 | AC096921.1 | 0.608 | < 0.001 |
| AC007255.1 | AC005083.1 | 0.608 | < 0.001 |
| AC007255.1 | CTNNA1P1 | 0.607 | < 0.001 |
| AC007255.1 | EPS15-AS1 | 0.607 | < 0.001 |
| AC007255.1 | RNF157 | 0.607 | < 0.001 |
| AC007255.1 | ZMYND12 | 0.607 | < 0.001 |
| AC007255.1 | CENPV | 0.607 | < 0.001 |
| AC007255.1 | AC108729.2 | 0.607 | < 0.001 |
| AC007255.1 | TAC3 | 0.607 | < 0.001 |
| AC007255.1 | ME3 | 0.607 | < 0.001 |
| AC007255.1 | UBXN10 | 0.607 | < 0.001 |
| AC007255.1 | CFB | 0.606 | < 0.001 |
| AC007255.1 | RASSF3 | 0.606 | < 0.001 |
| AC007255.1 | HMGN2P46 | 0.606 | < 0.001 |
| AC007255.1 | DNAJC1 | 0.606 | < 0.001 |
| AC007255.1 | ZNF33A | 0.606 | < 0.001 |
| AC007255.1 | PCDH1 | 0.606 | < 0.001 |
| AC007255.1 | SHH | 0.606 | < 0.001 |
| AC007255.1 | AC004233.1 | 0.606 | < 0.001 |
| AC007255.1 | CYB561D2 | 0.606 | < 0.001 |
| AC007255.1 | CCR5AS | 0.606 | < 0.001 |
| AC007255.1 | FN3K | 0.606 | < 0.001 |
| AC007255.1 | AL359382.1 | 0.605 | < 0.001 |
| AC007255.1 | SMLR1 | 0.605 | < 0.001 |
| AC007255.1 | SPATA2 | 0.605 | < 0.001 |
| AC007255.1 | NHLRC3 | 0.605 | < 0.001 |
| AC007255.1 | CTSE | 0.605 | < 0.001 |
| AC007255.1 | SSUH2 | 0.605 | < 0.001 |
| AC007255.1 | CYP2T3P | 0.605 | < 0.001 |
| AC007255.1 | POC1B-AS1 | 0.604 | < 0.001 |
| AC007255.1 | AC140479.2 | 0.604 | < 0.001 |
| AC007255.1 | SLC39A7 | 0.604 | < 0.001 |
| AC007255.1 | AC092436.3 | 0.604 | < 0.001 |
| AC007255.1 | UGT2B15 | 0.604 | < 0.001 |
| AC007255.1 | MGST3 | 0.604 | < 0.001 |
| AC007255.1 | DBNDD2 | 0.603 | < 0.001 |
| AC007255.1 | AL139246.2 | 0.603 | < 0.001 |
| AC007255.1 | NECTIN3 | 0.603 | < 0.001 |
| AC007255.1 | KALRN | 0.603 | < 0.001 |
| AC007255.1 | TEX49 | 0.603 | < 0.001 |
| AC007255.1 | AC108112.1 | 0.603 | < 0.001 |
| AC007255.1 | CLDN12 | 0.603 | < 0.001 |
| AC007255.1 | FAM166C | 0.603 | < 0.001 |
| AC007255.1 | GOLPH3L | 0.603 | < 0.001 |
| AC007255.1 | KLHDC7A | 0.603 | < 0.001 |
| AC007255.1 | AQP5 | 0.603 | < 0.001 |
| AC007255.1 | RN7SL351P | 0.603 | < 0.001 |
| AC007255.1 | MGAT3 | 0.603 | < 0.001 |
| AC007255.1 | AC011379.1 | 0.603 | < 0.001 |
| AC007255.1 | THRA | 0.602 | < 0.001 |
| AC007255.1 | TMEM50B | 0.602 | < 0.001 |
| AC007255.1 | FOXA2 | 0.602 | < 0.001 |
| AC007255.1 | TNIK | 0.602 | < 0.001 |
| AC007255.1 | ABCD3 | 0.602 | < 0.001 |
| AC007255.1 | AC016292.1 | 0.602 | < 0.001 |
| AC007255.1 | SNRPCP13 | 0.602 | < 0.001 |
| AC007255.1 | TRAF4 | 0.602 | < 0.001 |
| AC007255.1 | GOLGA2P8 | 0.602 | < 0.001 |
| AC007255.1 | AC000111.2 | 0.602 | < 0.001 |
| AC007255.1 | GALC | 0.601 | < 0.001 |
| AC007255.1 | CMTM4 | 0.601 | < 0.001 |
| AC007255.1 | MTND5P25 | 0.601 | < 0.001 |
| AC007255.1 | PPM1B | 0.601 | < 0.001 |
| AC007255.1 | SYNJ2 | 0.601 | < 0.001 |
| AC007255.1 | HIBADH | 0.601 | < 0.001 |
| AC007255.1 | AL589765.6 | 0.601 | < 0.001 |
| AC007255.1 | IQGAP3 | 0.601 | < 0.001 |
| AC007255.1 | BCKDHB | 0.601 | < 0.001 |
| AC007255.1 | CCL28 | 0.600 | < 0.001 |
| AC007255.1 | VWA7 | 0.600 | < 0.001 |
| AC007255.1 | MIR6774 | 0.600 | < 0.001 |
| AC007255.1 | GET4 | 0.600 | < 0.001 |
| AC007255.1 | AP4B1-AS1 | 0.600 | < 0.001 |
| AC007255.1 | AL355076.2 | 0.600 | < 0.001 |
| AC007255.1 | LINC02826 | 0.599 | < 0.001 |
| AC007255.1 | KRT18P17 | 0.599 | < 0.001 |
| AC007255.1 | CCDC125 | 0.599 | < 0.001 |
| AC007255.1 | STX3 | 0.599 | < 0.001 |
| AC007255.1 | AC005104.2 | 0.599 | < 0.001 |
| AC007255.1 | RAB3IP | 0.599 | < 0.001 |
| AC007255.1 | RNU4-78P | 0.599 | < 0.001 |
| AC007255.1 | AC012354.10 | 0.599 | < 0.001 |
| AC007255.1 | AMBP | 0.598 | < 0.001 |
| AC007255.1 | KRT18P18 | 0.598 | < 0.001 |
| AC007255.1 | ANO7 | 0.598 | < 0.001 |
| AC007255.1 | COX19 | 0.598 | < 0.001 |
| AC007255.1 | ACAA2 | 0.598 | < 0.001 |
| AC007255.1 | C3orf35 | 0.597 | < 0.001 |
| AC007255.1 | TMEM150B | 0.597 | < 0.001 |
| AC007255.1 | SI | 0.597 | < 0.001 |
| AC007255.1 | FAR2 | 0.597 | < 0.001 |
| AC007255.1 | MAGI1 | 0.597 | < 0.001 |
| AC007255.1 | UMAD1 | 0.597 | < 0.001 |
| AC007255.1 | CCT4P2 | 0.597 | < 0.001 |
| AC007255.1 | RNU6-46P | 0.597 | < 0.001 |
| AC007255.1 | FXYD4 | 0.596 | < 0.001 |
| AC007255.1 | ARFGEF2 | 0.596 | < 0.001 |
| AC007255.1 | GUSB | 0.596 | < 0.001 |
| AC007255.1 | CLDN9 | 0.596 | < 0.001 |
| AC007255.1 | AC005280.1 | 0.596 | < 0.001 |
| AC007255.1 | PARD3B | 0.596 | < 0.001 |
| AC007255.1 | AL592546.2 | 0.596 | < 0.001 |
| AC007255.1 | LINC02363 | 0.596 | < 0.001 |
| AC007255.1 | MYORG | 0.596 | < 0.001 |
| AC007255.1 | CCL15 | 0.596 | < 0.001 |
| AC007255.1 | MBOAT1 | 0.595 | < 0.001 |
| AC007255.1 | FCGRT | 0.595 | < 0.001 |
| AC007255.1 | MESTIT1 | 0.595 | < 0.001 |
| AC007255.1 | AC015884.3 | 0.595 | < 0.001 |
| AC007255.1 | DPY19L2P3 | 0.595 | < 0.001 |
| AC007255.1 | SNAP23 | 0.595 | < 0.001 |
| AC007255.1 | ZFYVE27 | 0.595 | < 0.001 |
| AC007255.1 | FUCA2 | 0.595 | < 0.001 |
| AC007255.1 | LINC02585 | 0.595 | < 0.001 |
| AC007255.1 | ATP2A3 | 0.594 | < 0.001 |
| AC007255.1 | AP001148.1 | 0.594 | < 0.001 |
| AC007255.1 | NANOGP7 | 0.594 | < 0.001 |
| AC007255.1 | SAPCD1 | 0.594 | < 0.001 |
| AC007255.1 | REG4 | 0.594 | < 0.001 |
| AC007255.1 | PNPLA2 | 0.594 | < 0.001 |
| AC007255.1 | CTAGE3P | 0.594 | < 0.001 |
| AC007255.1 | AQP2 | 0.594 | < 0.001 |
| AC007255.1 | AC026368.1 | 0.594 | < 0.001 |
| AC007255.1 | ZNF852 | 0.593 | < 0.001 |
| AC007255.1 | COL9A1 | 0.593 | < 0.001 |
| AC007255.1 | TMEM38A | 0.593 | < 0.001 |
| AC007255.1 | SEC22B | 0.593 | < 0.001 |
| AC007255.1 | SLC4A4 | 0.593 | < 0.001 |
| AC007255.1 | GNA14 | 0.593 | < 0.001 |
| AC007255.1 | AC010327.5 | 0.593 | < 0.001 |
| AC007255.1 | PHYH | 0.593 | < 0.001 |
| AC007255.1 | AC015971.1 | 0.593 | < 0.001 |
| AC007255.1 | AC079061.1 | 0.592 | < 0.001 |
| AC007255.1 | ZNF518A | 0.592 | < 0.001 |
| AC007255.1 | SEMA3B | 0.592 | < 0.001 |
| AC007255.1 | GRID2IP | 0.592 | < 0.001 |
| AC007255.1 | ANKRD10 | 0.592 | < 0.001 |
| AC007255.1 | PLCH1-AS1 | 0.592 | < 0.001 |
| AC007255.1 | AL033504.1 | 0.592 | < 0.001 |
| AC007255.1 | CXCL3 | 0.592 | < 0.001 |
| AC007255.1 | GLB1L2 | 0.592 | < 0.001 |
| AC007255.1 | OR7E14P | 0.592 | < 0.001 |
| AC007255.1 | ITPKA | 0.592 | < 0.001 |
| AC007255.1 | RNU6-1093P | 0.592 | < 0.001 |
| AC007255.1 | FICD | 0.592 | < 0.001 |
| AC007255.1 | AL135924.2 | 0.591 | < 0.001 |
| AC007255.1 | EVX1-AS | 0.591 | < 0.001 |
| AC007255.1 | PHGR1 | 0.591 | < 0.001 |
| AC007255.1 | GALE | 0.591 | < 0.001 |
| AC007255.1 | UGT2A3 | 0.591 | < 0.001 |
| AC007255.1 | USP3 | 0.591 | < 0.001 |
| AC007255.1 | ZNF468 | 0.591 | < 0.001 |
| AC007255.1 | AC022762.2 | 0.591 | < 0.001 |
| AC007255.1 | CEACAM5 | 0.591 | < 0.001 |
| AC007255.1 | AC010761.4 | 0.591 | < 0.001 |
| AC007255.1 | CATSPERB | 0.591 | < 0.001 |
| AC007255.1 | AC084026.2 | 0.590 | < 0.001 |
| AC007255.1 | TNFRSF14 | 0.590 | < 0.001 |
| AC007255.1 | AL133370.1 | 0.590 | < 0.001 |
| AC007255.1 | XKR9 | 0.590 | < 0.001 |
| AC007255.1 | POF1B | 0.590 | < 0.001 |
| AC007255.1 | C1orf195 | 0.590 | < 0.001 |
| AC007255.1 | SHD | 0.590 | < 0.001 |
| AC007255.1 | SAMD5 | 0.590 | < 0.001 |
| AC007255.1 | RIMKLA | 0.590 | < 0.001 |
| AC007255.1 | LINC02633 | 0.590 | < 0.001 |
| AC007255.1 | SLCO4A1-AS1 | 0.590 | < 0.001 |
| AC007255.1 | AL512288.1 | 0.590 | < 0.001 |
| AC007255.1 | AC105935.2 | 0.589 | < 0.001 |
| AC007255.1 | HEPACAM2 | 0.589 | < 0.001 |
| AC007255.1 | RFLNA | 0.589 | < 0.001 |
| AC007255.1 | HOXA11 | 0.589 | < 0.001 |
| AC007255.1 | LCOR | 0.589 | < 0.001 |
| AC007255.1 | UGT8 | 0.589 | < 0.001 |
| AC007255.1 | LGALS9 | 0.589 | < 0.001 |
| AC007255.1 | KBTBD11 | 0.589 | < 0.001 |
| AC007255.1 | KRT8P49 | 0.589 | < 0.001 |
| AC007255.1 | AC104336.1 | 0.589 | < 0.001 |
| AC007255.1 | IQGAP2 | 0.589 | < 0.001 |
| AC007255.1 | LINC00456 | 0.589 | < 0.001 |
| AC007255.1 | AL121992.3 | 0.589 | < 0.001 |
| AC007255.1 | SNX14 | 0.589 | < 0.001 |
| AC007255.1 | UPK3A | 0.588 | < 0.001 |
| AC007255.1 | LINC00570 | 0.588 | < 0.001 |
| AC007255.1 | SCCPDH | 0.588 | < 0.001 |
| AC007255.1 | HCAR1 | 0.588 | < 0.001 |
| AC007255.1 | KRT8P7 | 0.588 | < 0.001 |
| AC007255.1 | KRT8P8 | 0.588 | < 0.001 |
| AC007255.1 | CCDC68 | 0.588 | < 0.001 |
| AC007255.1 | KRT18P38 | 0.588 | < 0.001 |
| AC007255.1 | AC004080.15 | 0.588 | < 0.001 |
| AC007255.1 | APOBEC4 | 0.588 | < 0.001 |
| AC007255.1 | TTC6 | 0.588 | < 0.001 |
| AC007255.1 | ATP10B | 0.588 | < 0.001 |
| AC007255.1 | BRI3BP | 0.588 | < 0.001 |
| AC007255.1 | PBLD | 0.588 | < 0.001 |
| AC007255.1 | ZDHHC14 | 0.588 | < 0.001 |
| AC007255.1 | AL359183.1 | 0.587 | < 0.001 |
| AC007255.1 | RND1 | 0.587 | < 0.001 |
| AC007255.1 | LINC00870 | 0.587 | < 0.001 |
| AC007255.1 | PIGT | 0.587 | < 0.001 |
| AC007255.1 | RPS15AP30 | 0.587 | < 0.001 |
| AC007255.1 | SYNPR-AS1 | 0.587 | < 0.001 |
| AC007255.1 | AC087269.1 | 0.587 | < 0.001 |
| AC007255.1 | TRNT1 | 0.586 | < 0.001 |
| AC007255.1 | AC147055.1 | 0.586 | < 0.001 |
| AC007255.1 | TBC1D8B | 0.586 | < 0.001 |
| AC007255.1 | MGAM | 0.586 | < 0.001 |
| AC007255.1 | AP000919.2 | 0.586 | < 0.001 |
| AC007255.1 | XYLB | 0.586 | < 0.001 |
| AC007255.1 | AC005041.3 | 0.586 | < 0.001 |
| AC007255.1 | SRMS | 0.586 | < 0.001 |
| AC007255.1 | AC080037.2 | 0.586 | < 0.001 |
| AC007255.1 | AL158837.1 | 0.586 | < 0.001 |
| AC007255.1 | ZNF816 | 0.586 | < 0.001 |
| AC007255.1 | CANT1 | 0.586 | < 0.001 |
| AC007255.1 | AHCYL2 | 0.586 | < 0.001 |
| AC007255.1 | AMOT | 0.586 | < 0.001 |
| AC007255.1 | IBTK | 0.586 | < 0.001 |
| AC007255.1 | FNDC3A | 0.585 | < 0.001 |
| AC007255.1 | SH3RF1 | 0.585 | < 0.001 |
| AC007255.1 | AC019257.1 | 0.585 | < 0.001 |
| AC007255.1 | RNF128 | 0.585 | < 0.001 |
| AC007255.1 | ATOH1 | 0.585 | < 0.001 |
| AC007255.1 | IFT20 | 0.585 | < 0.001 |
| AC007255.1 | MYH14 | 0.585 | < 0.001 |
| AC007255.1 | LYZ | 0.584 | < 0.001 |
| AC007255.1 | AC090505.1 | 0.584 | < 0.001 |
| AC007255.1 | AC004594.1 | 0.584 | < 0.001 |
| AC007255.1 | SLC17A9 | 0.584 | < 0.001 |
| AC007255.1 | AL513174.1 | 0.584 | < 0.001 |
| AC007255.1 | AC087588.2 | 0.584 | < 0.001 |
| AC007255.1 | SPRY1 | 0.584 | < 0.001 |
| AC007255.1 | PRELID3B | 0.584 | < 0.001 |
| AC007255.1 | NPIPB14P | 0.583 | < 0.001 |
| AC007255.1 | PNRC2 | 0.583 | < 0.001 |
| AC007255.1 | CAMK2N1 | 0.583 | < 0.001 |
| AC007255.1 | CT83 | 0.583 | < 0.001 |
| AC007255.1 | AC137630.1 | 0.583 | < 0.001 |
| AC007255.1 | LINC02535 | 0.583 | < 0.001 |
| AC007255.1 | AGT | 0.583 | < 0.001 |
| AC007255.1 | MAGIX | 0.583 | < 0.001 |
| AC007255.1 | CCDC198 | 0.583 | < 0.001 |
| AC007255.1 | AC010487.1 | 0.583 | < 0.001 |
| AC007255.1 | DYRK2 | 0.583 | < 0.001 |
| AC007255.1 | PTMAP3 | 0.583 | < 0.001 |
| AC007255.1 | SEPHS2 | 0.583 | < 0.001 |
| AC007255.1 | LINC02453 | 0.582 | < 0.001 |
| AC007255.1 | KCTD14 | 0.582 | < 0.001 |
| AC007255.1 | CALR4P | 0.582 | < 0.001 |
| AC007255.1 | CEACAM18 | 0.582 | < 0.001 |
| AC007255.1 | NSUN7 | 0.582 | < 0.001 |
| AC007255.1 | EIF3J-DT | 0.582 | < 0.001 |
| AC007255.1 | AC005041.2 | 0.582 | < 0.001 |
| AC007255.1 | AC010976.2 | 0.582 | < 0.001 |
| AC007255.1 | RNF125 | 0.581 | < 0.001 |
| AC007255.1 | AC091544.5 | 0.581 | < 0.001 |
| AC007255.1 | LRATD2 | 0.581 | < 0.001 |
| AC007255.1 | GYG2 | 0.581 | < 0.001 |
| AC007255.1 | BAIAP2L2 | 0.581 | < 0.001 |
| AC007255.1 | SLC9A8 | 0.581 | < 0.001 |
| AC007255.1 | GAU1 | 0.581 | < 0.001 |
| AC007255.1 | LGALS3 | 0.581 | < 0.001 |
| AC007255.1 | ZMIZ2 | 0.581 | < 0.001 |
| AC007255.1 | MICU1 | 0.581 | < 0.001 |
| AC007255.1 | MIR4728 | 0.581 | < 0.001 |
| AC007255.1 | CADPS2 | 0.580 | < 0.001 |
| AC007255.1 | TMC7 | 0.580 | < 0.001 |
| AC007255.1 | SLC18B1 | 0.580 | < 0.001 |
| AC007255.1 | AL359955.1 | 0.580 | < 0.001 |
| AC007255.1 | LINC00261 | 0.580 | < 0.001 |
| AC007255.1 | PAQR8 | 0.580 | < 0.001 |
| AC007255.1 | PLLP | 0.580 | < 0.001 |
| AC007255.1 | ADGRG5 | 0.580 | < 0.001 |
| AC007255.1 | LINC02331 | 0.579 | < 0.001 |
| AC007255.1 | MYRFL | 0.579 | < 0.001 |
| AC007255.1 | AZGP1 | 0.579 | < 0.001 |
| AC007255.1 | AL590438.1 | 0.579 | < 0.001 |
| AC007255.1 | SLC43A1 | 0.579 | < 0.001 |
| AC007255.1 | C4BPA | 0.579 | < 0.001 |
| AC007255.1 | TMEM229A | 0.579 | < 0.001 |
| AC007255.1 | KRT7 | 0.579 | < 0.001 |
| AC007255.1 | TMEM92 | 0.579 | < 0.001 |
| AC007255.1 | NEK6 | 0.579 | < 0.001 |
| AC007255.1 | LY75 | 0.579 | < 0.001 |
| AC007255.1 | PPM1H | 0.578 | < 0.001 |
| AC007255.1 | AC135782.1 | 0.578 | < 0.001 |
| AC007255.1 | TPK1 | 0.578 | < 0.001 |
| AC007255.1 | TMEM170A | 0.578 | < 0.001 |
| AC007255.1 | MUC5AC | 0.578 | < 0.001 |
| AC007255.1 | EAF1 | 0.578 | < 0.001 |
| AC007255.1 | PRSS8 | 0.578 | < 0.001 |
| AC007255.1 | LINC01833 | 0.578 | < 0.001 |
| AC007255.1 | LINC00336 | 0.578 | < 0.001 |
| AC007255.1 | NEK8 | 0.577 | < 0.001 |
| AC007255.1 | LHFPL3-AS2 | 0.577 | < 0.001 |
| AC007255.1 | ASS1P10 | 0.577 | < 0.001 |
| AC007255.1 | ZBED3 | 0.577 | < 0.001 |
| AC007255.1 | GMPPB | 0.577 | < 0.001 |
| AC007255.1 | EPB41L1 | 0.577 | < 0.001 |
| AC007255.1 | AC012354.5 | 0.577 | < 0.001 |
| AC007255.1 | FAM86LP | 0.577 | < 0.001 |
| AC007255.1 | AC012354.3 | 0.577 | < 0.001 |
| AC007255.1 | AC010503.5 | 0.577 | < 0.001 |
| AC007255.1 | ACE | 0.577 | < 0.001 |
| AC007255.1 | CCT8P1 | 0.577 | < 0.001 |
| AC007255.1 | SLC6A19 | 0.577 | < 0.001 |
| AC007255.1 | ZNF75D | 0.577 | < 0.001 |
| AC007255.1 | AC007272.1 | 0.577 | < 0.001 |
| AC007255.1 | AL355353.2 | 0.577 | < 0.001 |
| AC007255.1 | SUSD1 | 0.576 | < 0.001 |
| AC007255.1 | PCK1 | 0.576 | < 0.001 |
| AC007255.1 | AL162377.2 | 0.576 | < 0.001 |
| AC007255.1 | NOS2 | 0.576 | < 0.001 |
| AC007255.1 | AL691482.2 | 0.576 | < 0.001 |
| AC007255.1 | NFATC2 | 0.576 | < 0.001 |
| AC007255.1 | MAN2A2 | 0.576 | < 0.001 |
| AC007255.1 | THUMPD3-AS1 | 0.576 | < 0.001 |
| AC007255.1 | CELF3 | 0.576 | < 0.001 |
| AC007255.1 | FABP1 | 0.576 | < 0.001 |
| AC007255.1 | TPRN | 0.576 | < 0.001 |
| AC007255.1 | VPS13C | 0.575 | < 0.001 |
| AC007255.1 | CDR2L | 0.575 | < 0.001 |
| AC007255.1 | RPIA | 0.575 | < 0.001 |
| AC007255.1 | SPATA13-AS1 | 0.575 | < 0.001 |
| AC007255.1 | SLC9A2 | 0.575 | < 0.001 |
| AC007255.1 | IL9RP3 | 0.575 | < 0.001 |
| AC007255.1 | CDC42EP5 | 0.575 | < 0.001 |
| AC007255.1 | FGFR1OP | 0.575 | < 0.001 |
| AC007255.1 | ATP11A | 0.575 | < 0.001 |
| AC007255.1 | CA8 | 0.575 | < 0.001 |
| AC007255.1 | HCG9 | 0.575 | < 0.001 |
| AC007255.1 | ARHGAP44 | 0.575 | < 0.001 |
| AC007255.1 | KRT8P20 | 0.575 | < 0.001 |
| AC007255.1 | GATA4 | 0.575 | < 0.001 |
| AC007255.1 | DUXAP1 | 0.574 | < 0.001 |
| AC007255.1 | PEX11A | 0.574 | < 0.001 |
| AC007255.1 | DNAH12 | 0.574 | < 0.001 |
| AC007255.1 | VRTN | 0.574 | < 0.001 |
| AC007255.1 | AC010210.1 | 0.574 | < 0.001 |
| AC007255.1 | AC091167.6 | 0.574 | < 0.001 |
| AC007255.1 | PLEKHA7 | 0.574 | < 0.001 |
| AC007255.1 | AC012354.9 | 0.574 | < 0.001 |
| AC007255.1 | TLDC2 | 0.573 | < 0.001 |
| AC007255.1 | AC004080.1 | 0.573 | < 0.001 |
| AC007255.1 | ANKRD10-IT1 | 0.573 | < 0.001 |
| AC007255.1 | KRT18P4 | 0.573 | < 0.001 |
| AC007255.1 | LHFPL3 | 0.573 | < 0.001 |
| AC007255.1 | PLAC8 | 0.573 | < 0.001 |
| AC007255.1 | ARHGAP42 | 0.572 | < 0.001 |
| AC007255.1 | NR2C1 | 0.572 | < 0.001 |
| AC007255.1 | C2CD2L | 0.572 | < 0.001 |
| AC007255.1 | F5 | 0.572 | < 0.001 |
| AC007255.1 | DAG1 | 0.572 | < 0.001 |
| AC007255.1 | AC002451.1 | 0.572 | < 0.001 |
| AC007255.1 | AL391845.2 | 0.572 | < 0.001 |
| AC007255.1 | C11orf86 | 0.572 | < 0.001 |
| AC007255.1 | KANSL1L | 0.572 | < 0.001 |
| AC007255.1 | GPD1L | 0.572 | < 0.001 |
| AC007255.1 | SFTA2 | 0.572 | < 0.001 |
| AC007255.1 | SH3KBP1 | 0.572 | < 0.001 |
| AC007255.1 | SPATA12 | 0.571 | < 0.001 |
| AC007255.1 | ARRDC1 | 0.571 | < 0.001 |
| AC007255.1 | AC021739.2 | 0.571 | < 0.001 |
| AC007255.1 | MSLN | 0.571 | < 0.001 |
| AC007255.1 | PCYT2 | 0.571 | < 0.001 |
| AC007255.1 | RPS6KA3 | 0.571 | < 0.001 |
| AC007255.1 | AL445183.2 | 0.571 | < 0.001 |
| AC007255.1 | ALDH18A1 | 0.571 | < 0.001 |
| AC007255.1 | INSC | 0.571 | < 0.001 |
| AC007255.1 | AC016542.1 | 0.570 | < 0.001 |
| AC007255.1 | MYO5B | 0.570 | < 0.001 |
| AC007255.1 | AL353747.3 | 0.570 | < 0.001 |
| AC007255.1 | AC138932.2 | 0.570 | < 0.001 |
| AC007255.1 | ACTBP13 | 0.570 | < 0.001 |
| AC007255.1 | PCSK7 | 0.570 | < 0.001 |
| AC007255.1 | SGSM1 | 0.570 | < 0.001 |
| AC007255.1 | TAX1BP1 | 0.570 | < 0.001 |
| AC007255.1 | CDX1 | 0.570 | < 0.001 |
| AC007255.1 | NADK | 0.570 | < 0.001 |
| AC007255.1 | TPD52 | 0.570 | < 0.001 |
| AC007255.1 | LINC01106 | 0.570 | < 0.001 |
| AC007255.1 | MFSD4A | 0.569 | < 0.001 |
| AC007255.1 | GRAMD1B | 0.569 | < 0.001 |
| AC007255.1 | CHMP4B | 0.569 | < 0.001 |
| AC007255.1 | ZNF195 | 0.569 | < 0.001 |
| AC007255.1 | AC005753.1 | 0.569 | < 0.001 |
| AC007255.1 | AC092198.1 | 0.569 | < 0.001 |
| AC007255.1 | TMPRSS5 | 0.569 | < 0.001 |
| AC007255.1 | FOXA1 | 0.569 | < 0.001 |
| AC007255.1 | PAPSS2 | 0.569 | < 0.001 |
| AC007255.1 | BSPRY | 0.569 | < 0.001 |
| AC007255.1 | AC072052.1 | 0.569 | < 0.001 |
| AC007255.1 | RNA5SP311 | 0.569 | < 0.001 |
| AC007255.1 | ABHD16A | 0.569 | < 0.001 |
| AC007255.1 | ERBB2 | 0.569 | < 0.001 |
| AC007255.1 | PTPRB | 0.568 | < 0.001 |
| AC007255.1 | TRABD2A | 0.568 | < 0.001 |
| AC007255.1 | NUDT16P1 | 0.568 | < 0.001 |
| AC007255.1 | AL121839.1 | 0.568 | < 0.001 |
| AC007255.1 | KCNN4 | 0.568 | < 0.001 |
| AC007255.1 | TFF2 | 0.568 | < 0.001 |
| AC007255.1 | REG1A | 0.568 | < 0.001 |
| AC007255.1 | SH2D4A | 0.568 | < 0.001 |
| AC007255.1 | AC023090.1 | 0.568 | < 0.001 |
| AC007255.1 | ACHE | 0.568 | < 0.001 |
| AC007255.1 | AC243964.3 | 0.568 | < 0.001 |
| AC007255.1 | KDELR3 | 0.568 | < 0.001 |
| AC007255.1 | AL136231.1 | 0.567 | < 0.001 |
| AC007255.1 | TTC13 | 0.567 | < 0.001 |
| AC007255.1 | CHD7 | 0.567 | < 0.001 |
| AC007255.1 | AL024508.1 | 0.566 | < 0.001 |
| AC007255.1 | P2RX4 | 0.566 | < 0.001 |
| AC007255.1 | AGAP13P | 0.566 | < 0.001 |
| AC007255.1 | CCR9 | 0.566 | < 0.001 |
| AC007255.1 | OSBPL2 | 0.566 | < 0.001 |
| AC007255.1 | CYCSP34 | 0.566 | < 0.001 |
| AC007255.1 | KRT18P23 | 0.566 | < 0.001 |
| AC007255.1 | SLC35B3 | 0.566 | < 0.001 |
| AC007255.1 | TRIM36 | 0.566 | < 0.001 |
| AC007255.1 | ANKRD28 | 0.566 | < 0.001 |
| AC007255.1 | GAK | 0.566 | < 0.001 |
| AC007255.1 | LINC00494 | 0.566 | < 0.001 |
| AC007255.1 | AC002401.3 | 0.566 | < 0.001 |
| AC007255.1 | VWA3B | 0.566 | < 0.001 |
| AC007255.1 | KRT18P39 | 0.565 | < 0.001 |
| AC007255.1 | TCTA | 0.565 | < 0.001 |
| AC007255.1 | Z84723.1 | 0.565 | < 0.001 |
| AC007255.1 | AFDN-DT | 0.565 | < 0.001 |
| AC007255.1 | AC015743.1 | 0.565 | < 0.001 |
| AC007255.1 | AL008733.1 | 0.565 | < 0.001 |
| AC007255.1 | KRTAP5-4 | 0.565 | < 0.001 |
| AC007255.1 | AL158071.3 | 0.565 | < 0.001 |
| AC007255.1 | CATIP-AS2 | 0.565 | < 0.001 |
| AC007255.1 | MGRN1 | 0.565 | < 0.001 |
| AC007255.1 | VASP | 0.564 | < 0.001 |
| AC007255.1 | PRKCG | 0.564 | < 0.001 |
| AC007255.1 | INSR | 0.564 | < 0.001 |
| AC007255.1 | ZNF443 | 0.564 | < 0.001 |
| AC007255.1 | AC006449.3 | 0.564 | < 0.001 |
| AC007255.1 | CTSS | 0.564 | < 0.001 |
| AC007255.1 | C6orf223 | 0.564 | < 0.001 |
| AC007255.1 | ARX | 0.564 | < 0.001 |
| AC007255.1 | AP006748.1 | 0.564 | < 0.001 |
| AC007255.1 | AL138756.1 | 0.564 | < 0.001 |
| AC007255.1 | PARP4 | 0.563 | < 0.001 |
| AC007255.1 | SLC35A2 | 0.563 | < 0.001 |
| AC007255.1 | TTC39A-AS1 | 0.563 | < 0.001 |
| AC007255.1 | MGAT4B | 0.563 | < 0.001 |
| AC007255.1 | SMCO4 | 0.563 | < 0.001 |
| AC007255.1 | AC091806.1 | 0.563 | < 0.001 |
| AC007255.1 | AC011298.1 | 0.563 | < 0.001 |
| AC007255.1 | TST | 0.563 | < 0.001 |
| AC007255.1 | YIPF6 | 0.563 | < 0.001 |
| AC007255.1 | DENND1C | 0.563 | < 0.001 |
| AC007255.1 | AC080129.2 | 0.563 | < 0.001 |
| AC007255.1 | PRKCA | 0.563 | < 0.001 |
| AC007255.1 | AL352984.1 | 0.563 | < 0.001 |
| AC007255.1 | SCML1 | 0.563 | < 0.001 |
| AC007255.1 | AP1M2 | 0.563 | < 0.001 |
| AC007255.1 | AC089999.4 | 0.563 | < 0.001 |
| AC007255.1 | VRK3 | 0.562 | < 0.001 |
| AC007255.1 | WASF1P1 | 0.562 | < 0.001 |
| AC007255.1 | DGAT2L7P | 0.562 | < 0.001 |
| AC007255.1 | SLC52A3 | 0.562 | < 0.001 |
| AC007255.1 | SLC26A3 | 0.562 | < 0.001 |
| AC007255.1 | PDE11A | 0.562 | < 0.001 |
| AC007255.1 | MYBPC3 | 0.562 | < 0.001 |
| AC007255.1 | KANSL1L-AS1 | 0.562 | < 0.001 |
| AC007255.1 | PNMA2 | 0.562 | < 0.001 |
| AC007255.1 | AC095031.1 | 0.562 | < 0.001 |
| AC007255.1 | NR3C2 | 0.562 | < 0.001 |
| AC007255.1 | BRCC3 | 0.562 | < 0.001 |
| AC007255.1 | ZC2HC1C | 0.562 | < 0.001 |
| AC007255.1 | SLC25A38 | 0.562 | < 0.001 |
| AC007255.1 | RNU6-702P | 0.562 | < 0.001 |
| AC007255.1 | ROCK2 | 0.562 | < 0.001 |
| AC007255.1 | EPB41L4B | 0.561 | < 0.001 |
| AC007255.1 | AC012354.8 | 0.561 | < 0.001 |
| AC007255.1 | HOXB13 | 0.561 | < 0.001 |
| AC007255.1 | SNHG22 | 0.561 | < 0.001 |
| AC007255.1 | SPR | 0.561 | < 0.001 |
| AC007255.1 | GOLGA4 | 0.561 | < 0.001 |
| AC007255.1 | CYMP | 0.561 | < 0.001 |
| AC007255.1 | NOXA1 | 0.561 | < 0.001 |
| AC007255.1 | LYN | 0.561 | < 0.001 |
| AC007255.1 | AC007001.1 | 0.561 | < 0.001 |
| AC007255.1 | ARMC3 | 0.561 | < 0.001 |
| AC007255.1 | AL583722.1 | 0.561 | < 0.001 |
| AC007255.1 | AC010627.1 | 0.561 | < 0.001 |
| AC007255.1 | AC141002.1 | 0.561 | < 0.001 |
| AC007255.1 | AC004264.1 | 0.561 | < 0.001 |
| AC007255.1 | SMIM32 | 0.560 | < 0.001 |
| AC007255.1 | AC009686.2 | 0.560 | < 0.001 |
| AC007255.1 | KIF21B | 0.560 | < 0.001 |
| AC007255.1 | AL450344.2 | 0.560 | < 0.001 |
| AC007255.1 | AC012467.2 | 0.560 | < 0.001 |
| AC007255.1 | MROH3P | 0.560 | < 0.001 |
| AC007255.1 | CHST4 | 0.560 | < 0.001 |
| AC007255.1 | TSGA10 | 0.560 | < 0.001 |
| AC007255.1 | AC093797.1 | 0.560 | < 0.001 |
| AC007255.1 | AC027607.1 | 0.560 | < 0.001 |
| AC007255.1 | MIR4701 | 0.560 | < 0.001 |
| AC007255.1 | KRT18P19 | 0.559 | < 0.001 |
| AC007255.1 | AC010547.2 | 0.559 | < 0.001 |
| AC007255.1 | MUC12 | 0.559 | < 0.001 |
| AC007255.1 | PDSS2 | 0.559 | < 0.001 |
| AC007255.1 | ASS1P11 | 0.559 | < 0.001 |
| AC007255.1 | AC073592.10 | 0.559 | < 0.001 |
| AC007255.1 | C11orf42 | 0.559 | < 0.001 |
| AC007255.1 | SPTAN1 | 0.559 | < 0.001 |
| AC007255.1 | IGSF23 | 0.559 | < 0.001 |
| AC007255.1 | LINC01559 | 0.558 | < 0.001 |
| AC007255.1 | LINC01124 | 0.558 | < 0.001 |
| AC007255.1 | GSKIP | 0.558 | < 0.001 |
| AC007255.1 | AC009078.3 | 0.558 | < 0.001 |
| AC007255.1 | ZNF765 | 0.558 | < 0.001 |
| AC007255.1 | SCP2 | 0.558 | < 0.001 |
| AC007255.1 | RORC | 0.558 | < 0.001 |
| AC007255.1 | MIR326 | 0.558 | < 0.001 |
| AC007255.1 | ZNF792 | 0.558 | < 0.001 |
| AC007255.1 | CIB4 | 0.558 | < 0.001 |
| AC007255.1 | AL359715.3 | 0.558 | < 0.001 |
| AC007255.1 | CAMKMT | 0.558 | < 0.001 |
| AC007255.1 | TMEM176A | 0.558 | < 0.001 |
| AC007255.1 | ABHD14A-ACY1 | 0.558 | < 0.001 |
| AC007255.1 | ZNF587 | 0.558 | < 0.001 |
| AC007255.1 | SLC5A9 | 0.558 | < 0.001 |
| AC007255.1 | RGS3 | 0.558 | < 0.001 |
| AC007255.1 | KIAA1211L | 0.558 | < 0.001 |
| AC007255.1 | ACACB | 0.557 | < 0.001 |
| AC007255.1 | KRT18P1 | 0.557 | < 0.001 |
| AC007255.1 | CFAP99 | 0.557 | < 0.001 |
| AC007255.1 | ZNF91 | 0.557 | < 0.001 |
| AC007255.1 | LRRC36 | 0.557 | < 0.001 |
| AC007255.1 | TRPC7-AS1 | 0.557 | < 0.001 |
| AC007255.1 | RNU6-1065P | 0.557 | < 0.001 |
| AC007255.1 | LINC02365 | 0.557 | < 0.001 |
| AC007255.1 | CATSPER2 | 0.557 | < 0.001 |
| AC007255.1 | AC010745.1 | 0.556 | < 0.001 |
| AC007255.1 | ZNF799 | 0.556 | < 0.001 |
| AC007255.1 | ZMYND15 | 0.556 | < 0.001 |
| AC007255.1 | MAP4K3 | 0.556 | < 0.001 |
| AC007255.1 | SLC26A6 | 0.556 | < 0.001 |
| AC007255.1 | ENPP7P8 | 0.556 | < 0.001 |
| AC007255.1 | AP2B1 | 0.556 | < 0.001 |
| AC007255.1 | ABHD2 | 0.556 | < 0.001 |
| AC007255.1 | RN7SL8P | 0.556 | < 0.001 |
| AC007255.1 | SLC29A3 | 0.556 | < 0.001 |
| AC007255.1 | KBTBD11-OT1 | 0.556 | < 0.001 |
| AC007255.1 | AC131097.2 | 0.556 | < 0.001 |
| AC007255.1 | ENPP1 | 0.556 | < 0.001 |
| AC007255.1 | Z73420.1 | 0.556 | < 0.001 |
| AC007255.1 | RASSF6 | 0.556 | < 0.001 |
| AC007255.1 | AC008555.7 | 0.555 | < 0.001 |
| AC007255.1 | AC098679.2 | 0.555 | < 0.001 |
| AC007255.1 | KRT8P5 | 0.555 | < 0.001 |
| AC007255.1 | AL512328.1 | 0.555 | < 0.001 |
| AC007255.1 | AC002401.2 | 0.555 | < 0.001 |
| AC007255.1 | HYKK | 0.555 | < 0.001 |
| AC007255.1 | AC121338.2 | 0.555 | < 0.001 |
| AC007255.1 | AL607028.1 | 0.555 | < 0.001 |
| AC007255.1 | VNN1 | 0.555 | < 0.001 |
| AC007255.1 | RPS12P2 | 0.555 | < 0.001 |
| AC007255.1 | SP5 | 0.555 | < 0.001 |
| AC007255.1 | RNU6-418P | 0.555 | < 0.001 |
| AC007255.1 | GAL3ST2 | 0.554 | < 0.001 |
| AC007255.1 | TTC21A | 0.554 | < 0.001 |
| AC007255.1 | SLC25A35 | 0.554 | < 0.001 |
| AC007255.1 | SERPINA1 | 0.554 | < 0.001 |
| AC007255.1 | AP003065.1 | 0.554 | < 0.001 |
| AC007255.1 | LY6G5C | 0.554 | < 0.001 |
| AC007255.1 | AC104534.1 | 0.554 | < 0.001 |
| AC007255.1 | GMPPA | 0.554 | < 0.001 |
| AC007255.1 | AC016542.3 | 0.554 | < 0.001 |
| AC007255.1 | LRRC37A6P | 0.554 | < 0.001 |
| AC007255.1 | WNK4 | 0.553 | < 0.001 |
| AC007255.1 | CELSR3 | 0.553 | < 0.001 |
| AC007255.1 | AC092145.1 | 0.553 | < 0.001 |
| AC007255.1 | AC006026.3 | 0.553 | < 0.001 |
| AC007255.1 | MIR135A1 | 0.553 | < 0.001 |
| AC007255.1 | SERPINA5 | 0.553 | < 0.001 |
| AC007255.1 | TRAF5 | 0.553 | < 0.001 |
| AC007255.1 | SYT13 | 0.553 | < 0.001 |
| AC007255.1 | SH2D6 | 0.553 | < 0.001 |
| AC007255.1 | AC122108.3 | 0.553 | < 0.001 |
| AC007255.1 | ZNF169 | 0.553 | < 0.001 |
| AC007255.1 | SULT1B1 | 0.553 | < 0.001 |
| AC007255.1 | C8orf49 | 0.552 | < 0.001 |
| AC007255.1 | PTPRN2 | 0.552 | < 0.001 |
| AC007255.1 | AL354872.2 | 0.552 | < 0.001 |
| AC007255.1 | NUDT13 | 0.552 | < 0.001 |
| AC007255.1 | TMEM51 | 0.552 | < 0.001 |
| AC007255.1 | SYCP3 | 0.552 | < 0.001 |
| AC007255.1 | MUC6 | 0.552 | < 0.001 |
| AC007255.1 | ANKRD61 | 0.551 | < 0.001 |
| AC007255.1 | SLC16A4 | 0.551 | < 0.001 |
| AC007255.1 | CYP4F12 | 0.551 | < 0.001 |
| AC007255.1 | TLN2 | 0.551 | < 0.001 |
| AC007255.1 | RPL17P17 | 0.551 | < 0.001 |
| AC007255.1 | AC016542.2 | 0.551 | < 0.001 |
| AC007255.1 | AK9 | 0.551 | < 0.001 |
| AC007255.1 | AL358232.1 | 0.551 | < 0.001 |
| AC007255.1 | LINC00884 | 0.551 | < 0.001 |
| AC007255.1 | SEZ6L2 | 0.551 | < 0.001 |
| AC007255.1 | SLC45A4 | 0.550 | < 0.001 |
| AC007255.1 | LINC01593 | 0.550 | < 0.001 |
| AC007255.1 | AC243967.1 | 0.550 | < 0.001 |
| AC007255.1 | AC006441.1 | 0.550 | < 0.001 |
| AC007255.1 | CLCN4 | 0.550 | < 0.001 |
| AC007255.1 | RPS24P14 | 0.550 | < 0.001 |
| AC007255.1 | AL035420.1 | 0.550 | < 0.001 |
| AC007255.1 | SLC35A1 | 0.550 | < 0.001 |
| AC007255.1 | AC015908.2 | 0.550 | < 0.001 |
| AC007255.1 | KRT18P20 | 0.550 | < 0.001 |
| AC007255.1 | ANXA11 | 0.550 | < 0.001 |
| AC007255.1 | KCNK15 | 0.550 | < 0.001 |
| AC007255.1 | ACY1 | 0.550 | < 0.001 |
| AC007255.1 | TOB1 | 0.549 | < 0.001 |
| AC007255.1 | GDE1 | 0.549 | < 0.001 |
| AC007255.1 | CCDC7 | 0.549 | < 0.001 |
| AC007255.1 | PALM3 | 0.549 | < 0.001 |
| AC007255.1 | MTND4P9 | 0.549 | < 0.001 |
| AC007255.1 | MLYCD | 0.549 | < 0.001 |
| AC007255.1 | OIT3 | 0.549 | < 0.001 |
| AC007255.1 | SLC7A7 | 0.549 | < 0.001 |
| AC007255.1 | PLA2G12B | 0.549 | < 0.001 |
| AC007255.1 | TJP2 | 0.548 | < 0.001 |
| AC007255.1 | SNX25P1 | 0.548 | < 0.001 |
| AC007255.1 | CCDC186 | 0.548 | < 0.001 |
| AC007255.1 | TAF4 | 0.548 | < 0.001 |
| AC007255.1 | NANS | 0.548 | < 0.001 |
| AC007255.1 | AC107072.2 | 0.548 | < 0.001 |
| AC007255.1 | PFKFB2 | 0.548 | < 0.001 |
| AC007255.1 | KLC4 | 0.548 | < 0.001 |
| AC007255.1 | AL136418.3 | 0.548 | < 0.001 |
| AC007255.1 | OLFM4 | 0.548 | < 0.001 |
| AC007255.1 | ZNF124 | 0.548 | < 0.001 |
| AC007255.1 | LRRC26 | 0.547 | < 0.001 |
| AC007255.1 | PTER | 0.547 | < 0.001 |
| AC007255.1 | LINC00513 | 0.547 | < 0.001 |
| AC007255.1 | C1orf194 | 0.547 | < 0.001 |
| AC007255.1 | FAM171A1 | 0.547 | < 0.001 |
| AC007255.1 | ZNF252P-AS1 | 0.547 | < 0.001 |
| AC007255.1 | CNTD1 | 0.547 | < 0.001 |
| AC007255.1 | TFPI | 0.547 | < 0.001 |
| AC007255.1 | MRPS25 | 0.547 | < 0.001 |
| AC007255.1 | ALG11 | 0.547 | < 0.001 |
| AC007255.1 | AL353747.2 | 0.547 | < 0.001 |
| AC007255.1 | AC231533.2 | 0.547 | < 0.001 |
| AC007255.1 | GMDS-DT | 0.547 | < 0.001 |
| AC007255.1 | GOLGA2P5 | 0.547 | < 0.001 |
| AC007255.1 | FGD4 | 0.547 | < 0.001 |
| AC007255.1 | ATG16L1 | 0.546 | < 0.001 |
| AC007255.1 | ABCC6P2 | 0.546 | < 0.001 |
| AC007255.1 | ACADS | 0.546 | < 0.001 |
| AC007255.1 | MIR4641 | 0.546 | < 0.001 |
| AC007255.1 | AC060780.1 | 0.546 | < 0.001 |
| AC007255.1 | SPRY2 | 0.546 | < 0.001 |
| AC007255.1 | E2F5 | 0.546 | < 0.001 |
| AC007255.1 | RAB11FIP1 | 0.545 | < 0.001 |
| AC007255.1 | AC069335.1 | 0.545 | < 0.001 |
| AC007255.1 | RNU6-850P | 0.545 | < 0.001 |
| AC007255.1 | DCTN1-AS1 | 0.545 | < 0.001 |
| AC007255.1 | BTN1A1 | 0.545 | < 0.001 |
| AC007255.1 | SPAG5-AS1 | 0.545 | < 0.001 |
| AC007255.1 | HMGCL | 0.545 | < 0.001 |
| AC007255.1 | OTULINL | 0.545 | < 0.001 |
| AC007255.1 | PRSS51 | 0.545 | < 0.001 |
| AC007255.1 | CELA3A | 0.545 | < 0.001 |
| AC007255.1 | TLX1 | 0.545 | < 0.001 |
| AC007255.1 | LARP4B | 0.544 | < 0.001 |
| AC007255.1 | TMEM181 | 0.544 | < 0.001 |
| AC007255.1 | TUSC2 | 0.544 | < 0.001 |
| AC007255.1 | CNOT11 | 0.544 | < 0.001 |
| AC007255.1 | KRT8P24 | 0.544 | < 0.001 |
| AC007255.1 | PTGER2 | 0.544 | < 0.001 |
| AC007255.1 | GBA2 | 0.544 | < 0.001 |
| AC007255.1 | DCLRE1C | 0.544 | < 0.001 |
| AC007255.1 | FRK | 0.544 | < 0.001 |
| AC007255.1 | NLK | 0.544 | < 0.001 |
| AC007255.1 | FEZF1-AS1 | 0.544 | < 0.001 |
| AC007255.1 | AC116025.2 | 0.544 | < 0.001 |
| AC007255.1 | TEP1 | 0.544 | < 0.001 |
| AC007255.1 | DAPK1 | 0.544 | < 0.001 |
| AC007255.1 | FARP2 | 0.544 | < 0.001 |
| AC007255.1 | OSBPL10 | 0.544 | < 0.001 |
| AC007255.1 | ANKMY1 | 0.543 | < 0.001 |
| AC007255.1 | CASP8 | 0.543 | < 0.001 |
| AC007255.1 | CLCN5 | 0.543 | < 0.001 |
| AC007255.1 | STK36 | 0.543 | < 0.001 |
| AC007255.1 | RBM6 | 0.543 | < 0.001 |
| AC007255.1 | ZNF56 | 0.543 | < 0.001 |
| AC007255.1 | DNMBP | 0.543 | < 0.001 |
| AC007255.1 | LINC01913 | 0.543 | < 0.001 |
| AC007255.1 | RNY1P16 | 0.543 | < 0.001 |
| AC007255.1 | ZNF28 | 0.543 | < 0.001 |
| AC007255.1 | PBX4 | 0.542 | < 0.001 |
| AC007255.1 | AC008171.1 | 0.542 | < 0.001 |
| AC007255.1 | BMP4 | 0.542 | < 0.001 |
| AC007255.1 | CCDC32 | 0.542 | < 0.001 |
| AC007255.1 | TM9SF2 | 0.542 | < 0.001 |
| AC007255.1 | AL445123.1 | 0.542 | < 0.001 |
| AC007255.1 | ZNF823 | 0.542 | < 0.001 |
| AC007255.1 | AXIN2 | 0.542 | < 0.001 |
| AC007255.1 | FFAR4 | 0.542 | < 0.001 |
| AC007255.1 | ALPG | 0.542 | < 0.001 |
| AC007255.1 | NEK4 | 0.542 | < 0.001 |
| AC007255.1 | KDELC1P1 | 0.542 | < 0.001 |
| AC007255.1 | MNX1-AS1 | 0.542 | < 0.001 |
| AC007255.1 | PTBP1P | 0.542 | < 0.001 |
| AC007255.1 | AC013355.1 | 0.542 | < 0.001 |
| AC007255.1 | PNLIPRP2 | 0.542 | < 0.001 |
| AC007255.1 | FCAMR | 0.542 | < 0.001 |
| AC007255.1 | LGALS2 | 0.541 | < 0.001 |
| AC007255.1 | AC006960.4 | 0.541 | < 0.001 |
| AC007255.1 | AC106827.2 | 0.541 | < 0.001 |
| AC007255.1 | AL391988.1 | 0.541 | < 0.001 |
| AC007255.1 | TDGF1 | 0.541 | < 0.001 |
| AC007255.1 | HENMT1 | 0.541 | < 0.001 |
| AC007255.1 | RETSAT | 0.541 | < 0.001 |
| AC007255.1 | C2orf50 | 0.541 | < 0.001 |
| AC007255.1 | KRT18P3 | 0.541 | < 0.001 |
| AC007255.1 | CABP4 | 0.541 | < 0.001 |
| AC007255.1 | TPCN1 | 0.541 | < 0.001 |
| AC007255.1 | LINC02344 | 0.540 | < 0.001 |
| AC007255.1 | ZFP36L2 | 0.540 | < 0.001 |
| AC007255.1 | GDPD5 | 0.540 | < 0.001 |
| AC007255.1 | LINC02352 | 0.540 | < 0.001 |
| AC007255.1 | STT3B | 0.540 | < 0.001 |
| AC007255.1 | TMEM97 | 0.540 | < 0.001 |
| AC007255.1 | CDK5RAP3 | 0.540 | < 0.001 |
| AC007255.1 | GAS2 | 0.540 | < 0.001 |
| AC007255.1 | FOXP4-AS1 | 0.540 | < 0.001 |
| AC007255.1 | AC022413.1 | 0.539 | < 0.001 |
| AC007255.1 | AL512383.1 | 0.539 | < 0.001 |
| AC007255.1 | AL136115.2 | 0.539 | < 0.001 |
| AC007255.1 | RPS27L | 0.539 | < 0.001 |
| AC007255.1 | HLA-J | 0.539 | < 0.001 |
| AC007255.1 | BRSK2 | 0.539 | < 0.001 |
| AC007255.1 | WWC1 | 0.539 | < 0.001 |
| AC007255.1 | RASGRP3 | 0.539 | < 0.001 |
| AC007255.1 | SLC27A2 | 0.539 | < 0.001 |
| AC007255.1 | CDC42BPA | 0.539 | < 0.001 |
| AC007255.1 | MUC2 | 0.539 | < 0.001 |
| AC007255.1 | CYP2B7P | 0.539 | < 0.001 |
| AC007255.1 | AC023389.2 | 0.539 | < 0.001 |
| AC007255.1 | BTBD16 | 0.538 | < 0.001 |
| AC007255.1 | RNU6-668P | 0.538 | < 0.001 |
| AC007255.1 | ST14 | 0.538 | < 0.001 |
| AC007255.1 | APTR | 0.538 | < 0.001 |
| AC007255.1 | ZNF600 | 0.538 | < 0.001 |
| AC007255.1 | MTRF1 | 0.537 | < 0.001 |
| AC007255.1 | AP1S3 | 0.537 | < 0.001 |
| AC007255.1 | PTGDR2 | 0.537 | < 0.001 |
| AC007255.1 | BTN2A1 | 0.537 | < 0.001 |
| AC007255.1 | FCHO1 | 0.537 | < 0.001 |
| AC007255.1 | LINC00342 | 0.537 | < 0.001 |
| AC007255.1 | SLC2A10 | 0.537 | < 0.001 |
| AC007255.1 | FEZF1 | 0.537 | < 0.001 |
| AC007255.1 | ZSWIM5 | 0.537 | < 0.001 |
| AC007255.1 | PLEKHB2 | 0.537 | < 0.001 |
| AC007255.1 | UNC93B1 | 0.537 | < 0.001 |
| AC007255.1 | ARSD | 0.537 | < 0.001 |
| AC007255.1 | CCK | 0.537 | < 0.001 |
| AC007255.1 | C20orf96 | 0.537 | < 0.001 |
| AC007255.1 | BCL2L1 | 0.537 | < 0.001 |
| AC007255.1 | AC090015.1 | 0.536 | < 0.001 |
| AC007255.1 | CHST5 | 0.536 | < 0.001 |
| AC007255.1 | RAB37 | 0.536 | < 0.001 |
| AC007255.1 | AC099518.4 | 0.536 | < 0.001 |
| AC007255.1 | ITLN1 | 0.536 | < 0.001 |
| AC007255.1 | AL353705.3 | 0.536 | < 0.001 |
| AC007255.1 | AC005532.2 | 0.536 | < 0.001 |
| AC007255.1 | AL591178.1 | 0.536 | < 0.001 |
| AC007255.1 | OTUD3 | 0.536 | < 0.001 |
| AC007255.1 | RN7SL19P | 0.536 | < 0.001 |
| AC007255.1 | HOXA10 | 0.536 | < 0.001 |
| AC007255.1 | ATP9A | 0.536 | < 0.001 |
| AC007255.1 | AC011481.2 | 0.536 | < 0.001 |
| AC007255.1 | ATE1 | 0.536 | < 0.001 |
| AC007255.1 | KIAA1324 | 0.535 | < 0.001 |
| AC007255.1 | BTD | 0.535 | < 0.001 |
| AC007255.1 | HEPH | 0.535 | < 0.001 |
| AC007255.1 | IGHV7-81 | 0.535 | < 0.001 |
| AC007255.1 | MUC5B | 0.535 | < 0.001 |
| AC007255.1 | AC008915.3 | 0.535 | < 0.001 |
| AC007255.1 | PLBD1 | 0.535 | < 0.001 |
| AC007255.1 | ODR4 | 0.535 | < 0.001 |
| AC007255.1 | RN7SKP25 | 0.535 | < 0.001 |
| AC007255.1 | DAGLA | 0.535 | < 0.001 |
| AC007255.1 | COQ8A | 0.535 | < 0.001 |
| AC007255.1 | ZFPM1 | 0.535 | < 0.001 |
| AC007255.1 | MYADML2 | 0.534 | < 0.001 |
| AC007255.1 | AC018809.1 | 0.534 | < 0.001 |
| AC007255.1 | CASC2 | 0.534 | < 0.001 |
| AC007255.1 | AL162724.1 | 0.534 | < 0.001 |
| AC007255.1 | CYBA | 0.534 | < 0.001 |
| AC007255.1 | TCAP | 0.534 | < 0.001 |
| AC007255.1 | CLUHP3 | 0.534 | < 0.001 |
| AC007255.1 | ERN1 | 0.534 | < 0.001 |
| AC007255.1 | POLR3GP1 | 0.534 | < 0.001 |
| AC007255.1 | AL671277.2 | 0.534 | < 0.001 |
| AC007255.1 | IMPG1 | 0.534 | < 0.001 |
| AC007255.1 | AC108751.4 | 0.534 | < 0.001 |
| AC007255.1 | MIR4635 | 0.533 | < 0.001 |
| AC007255.1 | CIR1P2 | 0.533 | < 0.001 |
| AC007255.1 | AC008379.1 | 0.533 | < 0.001 |
| AC007255.1 | DNAJC15 | 0.533 | < 0.001 |
| AC007255.1 | AC096633.1 | 0.533 | < 0.001 |
| AC007255.1 | CEACAM6 | 0.533 | < 0.001 |
| AC007255.1 | GK5 | 0.533 | < 0.001 |
| AC007255.1 | DPP4 | 0.533 | < 0.001 |
| AC007255.1 | AC016738.1 | 0.533 | < 0.001 |
| AC007255.1 | RNA5SP123 | 0.533 | < 0.001 |
| AC007255.1 | DUSP4 | 0.533 | < 0.001 |
| AC007255.1 | KRT8P35 | 0.533 | < 0.001 |
| AC007255.1 | DCP1A | 0.533 | < 0.001 |
| AC007255.1 | AC025265.3 | 0.533 | < 0.001 |
| AC007255.1 | UNC13B | 0.533 | < 0.001 |
| AC007255.1 | SLC25A19 | 0.533 | < 0.001 |
| AC007255.1 | EFCAB14 | 0.533 | < 0.001 |
| AC007255.1 | UBE2J1 | 0.532 | < 0.001 |
| AC007255.1 | C15orf65 | 0.532 | < 0.001 |
| AC007255.1 | AC093620.1 | 0.532 | < 0.001 |
| AC007255.1 | AC144836.1 | 0.532 | < 0.001 |
| AC007255.1 | STXBP6 | 0.532 | < 0.001 |
| AC007255.1 | MACC1-AS1 | 0.532 | < 0.001 |
| AC007255.1 | AL450344.3 | 0.532 | < 0.001 |
| AC007255.1 | SRPK1 | 0.532 | < 0.001 |
| AC007255.1 | AC103740.1 | 0.532 | < 0.001 |
| AC007255.1 | AL049612.1 | 0.532 | < 0.001 |
| AC007255.1 | TAOK3 | 0.532 | < 0.001 |
| AC007255.1 | RPL12P25 | 0.532 | < 0.001 |
| AC007255.1 | DNAH1 | 0.532 | < 0.001 |
| AC007255.1 | UTP14C | 0.532 | < 0.001 |
| AC007255.1 | NEUROG3 | 0.532 | < 0.001 |
| AC007255.1 | AGAP1-IT1 | 0.532 | < 0.001 |
| AC007255.1 | ASAP2 | 0.532 | < 0.001 |
| AC007255.1 | TNS3 | 0.532 | < 0.001 |
| AC007255.1 | ATP6V0A2 | 0.532 | < 0.001 |
| AC007255.1 | C9orf50 | 0.532 | < 0.001 |
| AC007255.1 | ALG5 | 0.531 | < 0.001 |
| AC007255.1 | LNX1-AS2 | 0.531 | < 0.001 |
| AC007255.1 | C20orf202 | 0.531 | < 0.001 |
| AC007255.1 | LINC02575 | 0.531 | < 0.001 |
| AC007255.1 | MLXIP | 0.531 | < 0.001 |
| AC007255.1 | TNFRSF11B | 0.531 | < 0.001 |
| AC007255.1 | TRPM4 | 0.531 | < 0.001 |
| AC007255.1 | AC005154.1 | 0.531 | < 0.001 |
| AC007255.1 | TMEM176B | 0.531 | < 0.001 |
| AC007255.1 | ZNF485 | 0.531 | < 0.001 |
| AC007255.1 | HOXB9 | 0.531 | < 0.001 |
| AC007255.1 | GUCA2A | 0.531 | < 0.001 |
| AC007255.1 | AC005274.1 | 0.531 | < 0.001 |
| AC007255.1 | TOR4A | 0.530 | < 0.001 |
| AC007255.1 | AC068658.1 | 0.530 | < 0.001 |
| AC007255.1 | AC068880.4 | 0.530 | < 0.001 |
| AC007255.1 | AC114772.1 | 0.530 | < 0.001 |
| AC007255.1 | AC139491.2 | 0.530 | < 0.001 |
| AC007255.1 | PRAC2 | 0.530 | < 0.001 |
| AC007255.1 | NR2F2 | 0.530 | < 0.001 |
| AC007255.1 | KRT18P68 | 0.530 | < 0.001 |
| AC007255.1 | CD55 | 0.530 | < 0.001 |
| AC007255.1 | TMEM248 | 0.530 | < 0.001 |
| AC007255.1 | AL627308.1 | 0.530 | < 0.001 |
| AC007255.1 | LINC01276 | 0.530 | < 0.001 |
| AC007255.1 | NKTR | 0.529 | < 0.001 |
| AC007255.1 | FAM3B | 0.529 | < 0.001 |
| AC007255.1 | CXorf38 | 0.529 | < 0.001 |
| AC007255.1 | AC026704.1 | 0.529 | < 0.001 |
| AC007255.1 | BAAT | 0.529 | < 0.001 |
| AC007255.1 | DOK7 | 0.529 | < 0.001 |
| AC007255.1 | MAGI1-AS1 | 0.529 | < 0.001 |
| AC007255.1 | ATP11A-AS1 | 0.529 | < 0.001 |
| AC007255.1 | STX7 | 0.529 | < 0.001 |
| AC007255.1 | FRMD3 | 0.529 | < 0.001 |
| AC007255.1 | AL162727.1 | 0.529 | < 0.001 |
| AC007255.1 | LINC00574 | 0.529 | < 0.001 |
| AC007255.1 | ARFGAP1 | 0.529 | < 0.001 |
| AC007255.1 | CDC42SE2 | 0.529 | < 0.001 |
| AC007255.1 | C11orf54 | 0.529 | < 0.001 |
| AC007255.1 | SLC19A3 | 0.529 | < 0.001 |
| AC007255.1 | INAVA | 0.529 | < 0.001 |
| AC007255.1 | USP3-AS1 | 0.528 | < 0.001 |
| AC007255.1 | AL139383.1 | 0.528 | < 0.001 |
| AC007255.1 | AC023024.1 | 0.528 | < 0.001 |
| AC007255.1 | FUT2 | 0.528 | < 0.001 |
| AC007255.1 | AL365271.1 | 0.528 | < 0.001 |
| AC007255.1 | KRT19 | 0.528 | < 0.001 |
| AC007255.1 | EFCAB5 | 0.528 | < 0.001 |
| AC007255.1 | LIF | 0.528 | < 0.001 |
| AC007255.1 | PRSS1 | 0.528 | < 0.001 |
| AC007255.1 | AC096751.2 | 0.527 | < 0.001 |
| AC007255.1 | TATDN2P2 | 0.527 | < 0.001 |
| AC007255.1 | SACM1L | 0.527 | < 0.001 |
| AC007255.1 | MTCO3P44 | 0.527 | < 0.001 |
| AC007255.1 | CPLX1 | 0.527 | < 0.001 |
| AC007255.1 | AC083843.3 | 0.527 | < 0.001 |
| AC007255.1 | ALG14 | 0.527 | < 0.001 |
| AC007255.1 | ARRDC2 | 0.527 | < 0.001 |
| AC007255.1 | GNE | 0.527 | < 0.001 |
| AC007255.1 | AC013489.1 | 0.527 | < 0.001 |
| AC007255.1 | MARVELD3 | 0.526 | < 0.001 |
| AC007255.1 | FAM160A2 | 0.526 | < 0.001 |
| AC007255.1 | AC104964.4 | 0.526 | < 0.001 |
| AC007255.1 | CYP3A4 | 0.526 | < 0.001 |
| AC007255.1 | FP325332.1 | 0.526 | < 0.001 |
| AC007255.1 | CTPS2 | 0.526 | < 0.001 |
| AC007255.1 | TMEM74B | 0.526 | < 0.001 |
| AC007255.1 | YBX2 | 0.526 | < 0.001 |
| AC007255.1 | LINC01011 | 0.526 | < 0.001 |
| AC007255.1 | HMGN2P28 | 0.526 | < 0.001 |
| AC007255.1 | CASC16 | 0.525 | < 0.001 |
| AC007255.1 | AL353807.1 | 0.525 | < 0.001 |
| AC007255.1 | URGCP | 0.525 | < 0.001 |
| AC007255.1 | C8orf31 | 0.525 | < 0.001 |
| AC007255.1 | TAAR1 | 0.525 | < 0.001 |
| AC007255.1 | KRT18P25 | 0.525 | < 0.001 |
| AC007255.1 | AC003965.2 | 0.525 | < 0.001 |
| AC007255.1 | AC091544.4 | 0.525 | < 0.001 |
| AC007255.1 | EHMT2-AS1 | 0.525 | < 0.001 |
| AC007255.1 | AC104958.2 | 0.525 | < 0.001 |
| AC007255.1 | RRN3P1 | 0.525 | < 0.001 |
| AC007255.1 | STK38 | 0.525 | < 0.001 |
| AC007255.1 | NCEH1 | 0.524 | < 0.001 |
| AC007255.1 | MEP1A | 0.524 | < 0.001 |
| AC007255.1 | ELFN1-AS1 | 0.524 | < 0.001 |
| AC007255.1 | KRT18P65 | 0.524 | < 0.001 |
| AC007255.1 | DHRS11 | 0.524 | < 0.001 |
| AC007255.1 | TMEM256P1 | 0.524 | < 0.001 |
| AC007255.1 | AC131888.1 | 0.524 | < 0.001 |
| AC007255.1 | ZNF35 | 0.524 | < 0.001 |
| AC007255.1 | AK7 | 0.524 | < 0.001 |
| AC007255.1 | LEFTY1 | 0.524 | < 0.001 |
| AC007255.1 | AL133406.1 | 0.524 | < 0.001 |
| AC007255.1 | DDAH2 | 0.524 | < 0.001 |
| AC007255.1 | CTH | 0.524 | < 0.001 |
| AC007255.1 | ANO5 | 0.524 | < 0.001 |
| AC007255.1 | MOGAT2 | 0.523 | < 0.001 |
| AC007255.1 | AL441964.1 | 0.523 | < 0.001 |
| AC007255.1 | LRP5 | 0.523 | < 0.001 |
| AC007255.1 | CISD3 | 0.523 | < 0.001 |
| AC007255.1 | GSE1 | 0.523 | < 0.001 |
| AC007255.1 | CCDC174 | 0.523 | < 0.001 |
| AC007255.1 | ZDHHC9 | 0.523 | < 0.001 |
| AC007255.1 | ZBTB18 | 0.523 | < 0.001 |
| AC007255.1 | AC046168.2 | 0.523 | < 0.001 |
| AC007255.1 | IFNL3P1 | 0.523 | < 0.001 |
| AC007255.1 | AL121583.1 | 0.523 | < 0.001 |
| AC007255.1 | Z99127.1 | 0.523 | < 0.001 |
| AC007255.1 | SYNE4 | 0.523 | < 0.001 |
| AC007255.1 | C2CD4A | 0.523 | < 0.001 |
| AC007255.1 | AL132657.1 | 0.523 | < 0.001 |
| AC007255.1 | AC108860.2 | 0.523 | < 0.001 |
| AC007255.1 | AC103702.1 | 0.522 | < 0.001 |
| AC007255.1 | CFTR-AS1 | 0.522 | < 0.001 |
| AC007255.1 | SLCO4A1 | 0.522 | < 0.001 |
| AC007255.1 | CNDP1 | 0.522 | < 0.001 |
| AC007255.1 | FBP2 | 0.522 | < 0.001 |
| AC007255.1 | AL590708.1 | 0.522 | < 0.001 |
| AC007255.1 | ZDHHC23 | 0.522 | < 0.001 |
| AC007255.1 | AL353807.4 | 0.522 | < 0.001 |
| AC007255.1 | AC010203.1 | 0.522 | < 0.001 |
| AC007255.1 | NR1H3 | 0.522 | < 0.001 |
| AC007255.1 | CAPN10-DT | 0.522 | < 0.001 |
| AC007255.1 | MTMR14 | 0.522 | < 0.001 |
| AC007255.1 | ABCC6 | 0.522 | < 0.001 |
| AC007255.1 | LINC00239 | 0.522 | < 0.001 |
| AC007255.1 | LCN2 | 0.522 | < 0.001 |
| AC007255.1 | ANXA9 | 0.522 | < 0.001 |
| AC007255.1 | MIR595 | 0.521 | < 0.001 |
| AC007255.1 | FGFRL1 | 0.521 | < 0.001 |
| AC007255.1 | HLA-W | 0.521 | < 0.001 |
| AC007255.1 | MTND4LP13 | 0.521 | < 0.001 |
| AC007255.1 | AATK | 0.521 | < 0.001 |
| AC007255.1 | NT5C3A | 0.521 | < 0.001 |
| AC007255.1 | AC108449.1 | 0.521 | < 0.001 |
| AC007255.1 | LINC00299 | 0.521 | < 0.001 |
| AC007255.1 | BOD1P1 | 0.521 | < 0.001 |
| AC007255.1 | TMEM238 | 0.521 | < 0.001 |
| AC007255.1 | HINT3 | 0.521 | < 0.001 |
| AC007255.1 | ALAS1 | 0.521 | < 0.001 |
| AC007255.1 | GPRIN3 | 0.521 | < 0.001 |
| AC007255.1 | CLDN18 | 0.520 | < 0.001 |
| AC007255.1 | ZNF763 | 0.520 | < 0.001 |
| AC007255.1 | FAM3D | 0.520 | < 0.001 |
| AC007255.1 | KIAA0319L | 0.520 | < 0.001 |
| AC007255.1 | CRLS1 | 0.520 | < 0.001 |
| AC007255.1 | AC008649.2 | 0.520 | < 0.001 |
| AC007255.1 | MARVELD2 | 0.520 | < 0.001 |
| AC007255.1 | RPH3AL | 0.520 | < 0.001 |
| AC007255.1 | AC007952.6 | 0.520 | < 0.001 |
| AC007255.1 | ARHGAP26-IT1 | 0.520 | < 0.001 |
| AC007255.1 | SRC | 0.520 | < 0.001 |
| AC007255.1 | ZNF587B | 0.520 | < 0.001 |
| AC007255.1 | NPM1P26 | 0.520 | < 0.001 |
| AC007255.1 | AC007849.1 | 0.520 | < 0.001 |
| AC007255.1 | C3orf85 | 0.520 | < 0.001 |
| AC007255.1 | PDXDC2P | 0.520 | < 0.001 |
| AC007255.1 | SIDT1 | 0.520 | < 0.001 |
| AC007255.1 | DPY19L1 | 0.519 | < 0.001 |
| AC007255.1 | CES3 | 0.519 | < 0.001 |
| AC007255.1 | AC096741.1 | 0.519 | < 0.001 |
| AC007255.1 | ZNRF2P2 | 0.519 | < 0.001 |
| AC007255.1 | AC080080.1 | 0.519 | < 0.001 |
| AC007255.1 | GAS2L3 | 0.519 | < 0.001 |
| AC007255.1 | MCF2L | 0.519 | < 0.001 |
| AC007255.1 | ERLEC1 | 0.519 | < 0.001 |
| AC007255.1 | MYB-AS1 | 0.519 | < 0.001 |
| AC007255.1 | AC121493.1 | 0.519 | < 0.001 |
| AC007255.1 | MAP3K21 | 0.519 | < 0.001 |
| AC007255.1 | AHI1 | 0.519 | < 0.001 |
| AC007255.1 | RNF114 | 0.519 | < 0.001 |
| AC007255.1 | AL136115.1 | 0.519 | < 0.001 |
| AC007255.1 | MIR3679 | 0.519 | < 0.001 |
| AC007255.1 | PGM3 | 0.518 | < 0.001 |
| AC007255.1 | AC023389.3 | 0.518 | < 0.001 |
| AC007255.1 | AC073651.1 | 0.518 | < 0.001 |
| AC007255.1 | AL596223.1 | 0.518 | < 0.001 |
| AC007255.1 | PIK3AP1 | 0.518 | < 0.001 |
| AC007255.1 | AP001351.1 | 0.518 | < 0.001 |
| AC007255.1 | MARCKSL1 | 0.518 | < 0.001 |
| AC007255.1 | CCNO | 0.518 | < 0.001 |
| AC007255.1 | DMBX1 | 0.518 | < 0.001 |
| AC007255.1 | DNAJC3 | 0.518 | < 0.001 |
| AC007255.1 | KRT18P29 | 0.518 | < 0.001 |
| AC007255.1 | MIA | 0.518 | < 0.001 |
| AC007255.1 | KIAA1958 | 0.518 | < 0.001 |
| AC007255.1 | AC022080.2 | 0.518 | < 0.001 |
| AC007255.1 | AC073333.1 | 0.517 | < 0.001 |
| AC007255.1 | CHMP4C | 0.517 | < 0.001 |
| AC007255.1 | BTNL9 | 0.517 | < 0.001 |
| AC007255.1 | CELA3B | 0.517 | < 0.001 |
| AC007255.1 | LINC02000 | 0.517 | < 0.001 |
| AC007255.1 | AC011411.1 | 0.517 | < 0.001 |
| AC007255.1 | ZNF444 | 0.517 | < 0.001 |
| AC007255.1 | KLHL23 | 0.517 | < 0.001 |
| AC007255.1 | AC010300.1 | 0.517 | < 0.001 |
| AC007255.1 | MACROD2 | 0.517 | < 0.001 |
| AC007255.1 | AC103591.2 | 0.517 | < 0.001 |
| AC007255.1 | TSEN54 | 0.517 | < 0.001 |
| AC007255.1 | AL121832.3 | 0.517 | < 0.001 |
| AC007255.1 | TENT5A | 0.517 | < 0.001 |
| AC007255.1 | VIPR1-AS1 | 0.517 | < 0.001 |
| AC007255.1 | RN7SL45P | 0.516 | < 0.001 |
| AC007255.1 | COG2 | 0.516 | < 0.001 |
| AC007255.1 | USP54 | 0.516 | < 0.001 |
| AC007255.1 | RN7SL749P | 0.516 | < 0.001 |
| AC007255.1 | UBE2R2-AS1 | 0.516 | < 0.001 |
| AC007255.1 | TMEM220-AS1 | 0.516 | < 0.001 |
| AC007255.1 | NPC1L1 | 0.516 | < 0.001 |
| AC007255.1 | TLX1NB | 0.516 | < 0.001 |
| AC007255.1 | DET1 | 0.516 | < 0.001 |
| AC007255.1 | AC104964.1 | 0.516 | < 0.001 |
| AC007255.1 | BTBD3 | 0.516 | < 0.001 |
| AC007255.1 | AC011477.2 | 0.516 | < 0.001 |
| AC007255.1 | AGAP1 | 0.516 | < 0.001 |
| AC007255.1 | VPS33B | 0.516 | < 0.001 |
| AC007255.1 | CD63 | 0.516 | < 0.001 |
| AC007255.1 | AC074138.1 | 0.516 | < 0.001 |
| AC007255.1 | AL121790.2 | 0.516 | < 0.001 |
| AC007255.1 | MTND3P5 | 0.515 | < 0.001 |
| AC007255.1 | HMGB1P23 | 0.515 | < 0.001 |
| AC007255.1 | TMEM214 | 0.515 | < 0.001 |
| AC007255.1 | TMBIM6 | 0.515 | < 0.001 |
| AC007255.1 | BPNT1 | 0.515 | < 0.001 |
| AC007255.1 | HSD11B2 | 0.515 | < 0.001 |
| AC007255.1 | AL021068.1 | 0.515 | < 0.001 |
| AC007255.1 | SLC25A45 | 0.515 | < 0.001 |
| AC007255.1 | AC078922.1 | 0.515 | < 0.001 |
| AC007255.1 | LBX2 | 0.515 | < 0.001 |
| AC007255.1 | LINC00858 | 0.515 | < 0.001 |
| AC007255.1 | LINC02421 | 0.515 | < 0.001 |
| AC007255.1 | AC103681.1 | 0.515 | < 0.001 |
| AC007255.1 | SYS1 | 0.515 | < 0.001 |
| AC007255.1 | LINC00881 | 0.515 | < 0.001 |
| AC007255.1 | OR13Z2P | 0.515 | < 0.001 |
| AC007255.1 | GMIP | 0.515 | < 0.001 |
| AC007255.1 | ZC4H2 | 0.515 | < 0.001 |
| AC007255.1 | C1orf220 | 0.515 | < 0.001 |
| AC007255.1 | GCKR | 0.515 | < 0.001 |
| AC007255.1 | AP000346.2 | 0.515 | < 0.001 |
| AC007255.1 | CYMP-AS1 | 0.515 | < 0.001 |
| AC007255.1 | FOXD2-AS1 | 0.514 | < 0.001 |
| AC007255.1 | LPCAT4 | 0.514 | < 0.001 |
| AC007255.1 | GXYLT1 | 0.514 | < 0.001 |
| AC007255.1 | RNF32 | 0.514 | < 0.001 |
| AC007255.1 | AQP12B | 0.514 | < 0.001 |
| AC007255.1 | MICALL2 | 0.514 | < 0.001 |
| AC007255.1 | LINC00323 | 0.514 | < 0.001 |
| AC007255.1 | FAM107B | 0.514 | < 0.001 |
| AC007255.1 | CTSH | 0.514 | < 0.001 |
| AC007255.1 | TMEM231P1 | 0.514 | < 0.001 |
| AC007255.1 | AC100791.3 | 0.514 | < 0.001 |
| AC007255.1 | LYPD6 | 0.514 | < 0.001 |
| AC007255.1 | CCPG1 | 0.514 | < 0.001 |
| AC007255.1 | NFE2L3 | 0.514 | < 0.001 |
| AC007255.1 | AC092168.2 | 0.514 | < 0.001 |
| AC007255.1 | AC087284.1 | 0.514 | < 0.001 |
| AC007255.1 | AC011773.2 | 0.514 | < 0.001 |
| AC007255.1 | NCLP1 | 0.514 | < 0.001 |
| AC007255.1 | AC244100.4 | 0.514 | < 0.001 |
| AC007255.1 | CFAP73 | 0.513 | < 0.001 |
| AC007255.1 | CTBP1-AS | 0.513 | < 0.001 |
| AC007255.1 | PRDM16-DT | 0.513 | < 0.001 |
| AC007255.1 | AC078942.1 | 0.513 | < 0.001 |
| AC007255.1 | IL17REL | 0.513 | < 0.001 |
| AC007255.1 | IVD | 0.513 | < 0.001 |
| AC007255.1 | PODXL | 0.513 | < 0.001 |
| AC007255.1 | KLHL32 | 0.513 | < 0.001 |
| AC007255.1 | TRIM54 | 0.513 | < 0.001 |
| AC007255.1 | NUDT4 | 0.513 | < 0.001 |
| AC007255.1 | AP001972.5 | 0.513 | < 0.001 |
| AC007255.1 | ABALON | 0.513 | < 0.001 |
| AC007255.1 | ASS1P7 | 0.512 | < 0.001 |
| AC007255.1 | RNU6-570P | 0.512 | < 0.001 |
| AC007255.1 | AC013268.3 | 0.512 | < 0.001 |
| AC007255.1 | BATF | 0.512 | < 0.001 |
| AC007255.1 | EDEM2 | 0.512 | < 0.001 |
| AC007255.1 | AC092338.1 | 0.512 | < 0.001 |
| AC007255.1 | ASS1P9 | 0.512 | < 0.001 |
| AC007255.1 | PDK4 | 0.512 | < 0.001 |
| AC007255.1 | CYP4V2 | 0.512 | < 0.001 |
| AC007255.1 | CYP2J2 | 0.512 | < 0.001 |
| AC007255.1 | AP003057.1 | 0.512 | < 0.001 |
| AC007255.1 | LINC01479 | 0.511 | < 0.001 |
| AC007255.1 | NEK10 | 0.511 | < 0.001 |
| AC007255.1 | RNF148 | 0.511 | < 0.001 |
| AC007255.1 | LRRC19 | 0.511 | < 0.001 |
| AC007255.1 | AP000640.1 | 0.511 | < 0.001 |
| AC007255.1 | LINC01648 | 0.511 | < 0.001 |
| AC007255.1 | CACNA2D4 | 0.511 | < 0.001 |
| AC007255.1 | KCTD6 | 0.511 | < 0.001 |
| AC007255.1 | POLM | 0.511 | < 0.001 |
| AC007255.1 | SFT2D1 | 0.511 | < 0.001 |
| AC007255.1 | ORAOV1P1 | 0.511 | < 0.001 |
| AC007255.1 | MCRIP2 | 0.511 | < 0.001 |
| AC007255.1 | TMEM164 | 0.510 | < 0.001 |
| AC007255.1 | RNU6-669P | 0.510 | < 0.001 |
| AC007255.1 | SARM1 | 0.510 | < 0.001 |
| AC007255.1 | RNU6-197P | 0.510 | < 0.001 |
| AC007255.1 | AC015688.4 | 0.510 | < 0.001 |
| AC007255.1 | THEM4 | 0.510 | < 0.001 |
| AC007255.1 | SUGT1P3 | 0.510 | < 0.001 |
| AC007255.1 | AC114291.1 | 0.510 | < 0.001 |
| AC007255.1 | MIR6740 | 0.510 | < 0.001 |
| AC007255.1 | ZNF189 | 0.510 | < 0.001 |
| AC007255.1 | ZNF587P1 | 0.510 | < 0.001 |
| AC007255.1 | ATF7IP2 | 0.510 | < 0.001 |
| AC007255.1 | PHOSPHO2 | 0.510 | < 0.001 |
| AC007255.1 | EPB41 | 0.510 | < 0.001 |
| AC007255.1 | PGAP3 | 0.510 | < 0.001 |
| AC007255.1 | KRT18P16 | 0.510 | < 0.001 |
| AC007255.1 | PCSK6 | 0.510 | < 0.001 |
| AC007255.1 | JADE1 | 0.510 | < 0.001 |
| AC007255.1 | AC007327.2 | 0.510 | < 0.001 |
| AC007255.1 | MIR3133 | 0.510 | < 0.001 |
| AC007255.1 | NRAP | 0.509 | < 0.001 |
| AC007255.1 | AP000919.3 | 0.509 | < 0.001 |
| AC007255.1 | RARA-AS1 | 0.509 | < 0.001 |
| AC007255.1 | LINC00514 | 0.509 | < 0.001 |
| AC007255.1 | EFCC1 | 0.509 | < 0.001 |
| AC007255.1 | PSMC1P2 | 0.509 | < 0.001 |
| AC007255.1 | AC010200.1 | 0.509 | < 0.001 |
| AC007255.1 | AL158166.2 | 0.509 | < 0.001 |
| AC007255.1 | FBXL14 | 0.509 | < 0.001 |
| AC007255.1 | AC002128.2 | 0.509 | < 0.001 |
| AC007255.1 | ZC3H12D | 0.509 | < 0.001 |
| AC007255.1 | CTSA | 0.508 | < 0.001 |
| AC007255.1 | AC018809.2 | 0.508 | < 0.001 |
| AC007255.1 | GKAP1 | 0.508 | < 0.001 |
| AC007255.1 | HOOK1 | 0.508 | < 0.001 |
| AC007255.1 | MAST3 | 0.508 | < 0.001 |
| AC007255.1 | BCLAF3 | 0.508 | < 0.001 |
| AC007255.1 | LINC00365 | 0.508 | < 0.001 |
| AC007255.1 | GRM8 | 0.508 | < 0.001 |
| AC007255.1 | TDRKH | 0.508 | < 0.001 |
| AC007255.1 | CAPN13 | 0.508 | < 0.001 |
| AC007255.1 | LSR | 0.508 | < 0.001 |
| AC007255.1 | AC004080.12 | 0.508 | < 0.001 |
| AC007255.1 | C12orf49 | 0.508 | < 0.001 |
| AC007255.1 | ANKRD36C | 0.508 | < 0.001 |
| AC007255.1 | ENPP7P2 | 0.508 | < 0.001 |
| AC007255.1 | HACL1 | 0.508 | < 0.001 |
| AC007255.1 | CXXC5 | 0.508 | < 0.001 |
| AC007255.1 | NARF-AS1 | 0.508 | < 0.001 |
| AC007255.1 | HTR1D | 0.507 | < 0.001 |
| AC007255.1 | PCGF5 | 0.507 | < 0.001 |
| AC007255.1 | OVOL2 | 0.507 | < 0.001 |
| AC007255.1 | AC068533.4 | 0.507 | < 0.001 |
| AC007255.1 | NPRL2 | 0.507 | < 0.001 |
| AC007255.1 | RABGAP1L-IT1 | 0.507 | < 0.001 |
| AC007255.1 | C6orf136 | 0.507 | < 0.001 |
| AC007255.1 | AL357033.3 | 0.507 | < 0.001 |
| AC007255.1 | SLC4A2 | 0.507 | < 0.001 |
| AC007255.1 | COLCA2 | 0.507 | < 0.001 |
| AC007255.1 | AC109460.2 | 0.507 | < 0.001 |
| AC007255.1 | FSCN2 | 0.507 | < 0.001 |
| AC007255.1 | DENND5B-AS1 | 0.507 | < 0.001 |
| AC007255.1 | RNF157-AS1 | 0.507 | < 0.001 |
| AC007255.1 | AC245884.8 | 0.507 | < 0.001 |
| AC007255.1 | AC018926.3 | 0.507 | < 0.001 |
| AC007255.1 | AL162724.2 | 0.506 | < 0.001 |
| AC007255.1 | AGAP4 | 0.506 | < 0.001 |
| AC007255.1 | KLHL25 | 0.506 | < 0.001 |
| AC007255.1 | DOCK5 | 0.506 | < 0.001 |
| AC007255.1 | C5orf30 | 0.506 | < 0.001 |
| AC007255.1 | AL390728.4 | 0.506 | < 0.001 |
| AC007255.1 | TTC39A | 0.506 | < 0.001 |
| AC007255.1 | AL133406.2 | 0.506 | < 0.001 |
| AC007255.1 | RPL15P21 | 0.506 | < 0.001 |
| AC007255.1 | KRT87P | 0.506 | < 0.001 |
| AC007255.1 | CATSPERG | 0.506 | < 0.001 |
| AC007255.1 | KPNA7 | 0.506 | < 0.001 |
| AC007255.1 | AIFM1 | 0.506 | < 0.001 |
| AC007255.1 | AL049552.1 | 0.506 | < 0.001 |
| AC007255.1 | AC090844.2 | 0.506 | < 0.001 |
| AC007255.1 | RPL23AP66 | 0.506 | < 0.001 |
| AC007255.1 | RCC2P6 | 0.506 | < 0.001 |
| AC007255.1 | EDN3 | 0.506 | < 0.001 |
| AC007255.1 | PSEN2 | 0.506 | < 0.001 |
| AC007255.1 | AL135844.1 | 0.506 | < 0.001 |
| AC007255.1 | AL590282.1 | 0.506 | < 0.001 |
| AC007255.1 | PRELID2 | 0.506 | < 0.001 |
| AC007255.1 | SLC30A1 | 0.505 | < 0.001 |
| AC007255.1 | FUT3 | 0.505 | < 0.001 |
| AC007255.1 | AC010973.2 | 0.505 | < 0.001 |
| AC007255.1 | GALK2 | 0.505 | < 0.001 |
| AC007255.1 | RAD18 | 0.504 | < 0.001 |
| AC007255.1 | LINC02532 | 0.504 | < 0.001 |
| AC007255.1 | MTMR9LP | 0.504 | < 0.001 |
| AC007255.1 | RAD51-AS1 | 0.504 | < 0.001 |
| AC007255.1 | FOXQ1 | 0.504 | < 0.001 |
| AC007255.1 | ITPK1 | 0.504 | < 0.001 |
| AC007255.1 | MIR657 | 0.504 | < 0.001 |
| AC007255.1 | AL359715.1 | 0.504 | < 0.001 |
| AC007255.1 | GRTP1 | 0.504 | < 0.001 |
| AC007255.1 | ZNF182 | 0.504 | < 0.001 |
| AC007255.1 | ZNF780A | 0.503 | < 0.001 |
| AC007255.1 | PLIN1 | 0.503 | < 0.001 |
| AC007255.1 | AL360219.1 | 0.503 | < 0.001 |
| AC007255.1 | NCAPGP1 | 0.503 | < 0.001 |
| AC007255.1 | ZNF493 | 0.503 | < 0.001 |
| AC007255.1 | PIGM | 0.503 | < 0.001 |
| AC007255.1 | AC009686.1 | 0.503 | < 0.001 |
| AC007255.1 | MIR647 | 0.503 | < 0.001 |
| AC007255.1 | CLDN15 | 0.503 | < 0.001 |
| AC007255.1 | PIWIL1 | 0.503 | < 0.001 |
| AC007255.1 | CAPN7 | 0.503 | < 0.001 |
| AC007255.1 | FOXO4 | 0.503 | < 0.001 |
| AC007255.1 | RN7SKP299 | 0.503 | < 0.001 |
| AC007255.1 | TGFBR2 | 0.503 | < 0.001 |
| AC007255.1 | AC091934.1 | 0.503 | < 0.001 |
| AC007255.1 | LINC01123 | 0.503 | < 0.001 |
| AC007255.1 | AL356652.1 | 0.503 | < 0.001 |
| AC007255.1 | SOX9 | 0.503 | < 0.001 |
| AC007255.1 | MUC4 | 0.503 | < 0.001 |
| AC007255.1 | TPM1-AS | 0.502 | < 0.001 |
| AC007255.1 | CYP2W1 | 0.502 | < 0.001 |
| AC007255.1 | TTPA | 0.502 | < 0.001 |
| AC007255.1 | NLRP6 | 0.502 | < 0.001 |
| AC007255.1 | PGC | 0.502 | < 0.001 |
| AC007255.1 | GPR39 | 0.502 | < 0.001 |
| AC007255.1 | TOE1 | 0.502 | < 0.001 |
| AC007255.1 | AL353622.1 | 0.502 | < 0.001 |
| AC007255.1 | AP001065.4 | 0.502 | < 0.001 |
| AC007255.1 | MIR4756 | 0.502 | < 0.001 |
| AC007255.1 | ABHD17C | 0.502 | < 0.001 |
| AC007255.1 | PTMAP1 | 0.502 | < 0.001 |
| AC007255.1 | OACYLP | 0.502 | < 0.001 |
| AC007255.1 | DPY19L1P2 | 0.501 | < 0.001 |
| AC007255.1 | AC073592.2 | 0.501 | < 0.001 |
| AC007255.1 | KRT18P35 | 0.501 | < 0.001 |
| AC007255.1 | STX18 | 0.501 | < 0.001 |
| AC007255.1 | SLC2A13 | 0.501 | < 0.001 |
| AC007255.1 | ARHGEF10L | 0.501 | < 0.001 |
| AC007255.1 | KIF28P | 0.501 | < 0.001 |
| AC007255.1 | AC022400.5 | 0.501 | < 0.001 |
| AC007255.1 | ATP2C2 | 0.501 | < 0.001 |
| AC007255.1 | AL162595.1 | 0.501 | < 0.001 |
| AC007255.1 | AQP6 | 0.501 | < 0.001 |
| AC007255.1 | PDE12 | 0.501 | < 0.001 |
| AC007255.1 | PANK1 | 0.501 | < 0.001 |
| AC007255.1 | BRPF3 | 0.501 | < 0.001 |
| AC007255.1 | AC003070.1 | 0.501 | < 0.001 |
| AC007255.1 | AC005154.4 | 0.501 | < 0.001 |
| AC007255.1 | HSD17B13 | 0.501 | < 0.001 |
| AC007255.1 | RNU6-1340P | 0.501 | < 0.001 |
| AC007255.1 | AC008456.1 | 0.501 | < 0.001 |
| AC007255.1 | AL158166.1 | 0.500 | < 0.001 |
| AC007255.1 | HNRNPA1P49 | 0.500 | < 0.001 |
| AC007255.1 | SLC37A4 | 0.500 | < 0.001 |
| AC007255.1 | GAB2 | 0.500 | < 0.001 |
| AC007255.1 | CALM2 | 0.500 | < 0.001 |
| AC007255.1 | CXCL2 | 0.500 | < 0.001 |
| AC007255.1 | PCSK6-AS1 | 0.500 | < 0.001 |
| AC007255.1 | AC098934.1 | 0.500 | < 0.001 |
| AC007255.1 | SLC5A1 | 0.500 | < 0.001 |
| AC007255.1 | TAMM41 | 0.500 | < 0.001 |
| AC007255.1 | ADAM28 | 0.500 | < 0.001 |
| AC007255.1 | KDELR1 | 0.500 | < 0.001 |
| AC007255.1 | MED23 | 0.500 | < 0.001 |
| AC007255.1 | AL009178.2 | 0.500 | < 0.001 |
| AC007255.1 | AL391056.1 | 0.500 | < 0.001 |
| AC007255.1 | FOXJ1 | 0.500 | < 0.001 |
| AC007255.1 | ACAA1 | 0.500 | < 0.001 |
| AC007255.1 | GALNT3 | 0.500 | < 0.001 |
| AC007255.1 | AL449106.1 | 0.500 | < 0.001 |
| AC007255.1 | YWHAB | 0.499 | < 0.001 |
| AC007255.1 | AFTPH | 0.499 | < 0.001 |
| AC007255.1 | AP000919.4 | 0.499 | < 0.001 |
| AC007255.1 | CPD | 0.499 | < 0.001 |
| AC007255.1 | AC120114.3 | 0.499 | < 0.001 |
| AC007255.1 | KRT8P13 | 0.499 | < 0.001 |
| AC007255.1 | LINC01504 | 0.499 | < 0.001 |
| AC007255.1 | SPDYC | 0.499 | < 0.001 |
| AC007255.1 | RASSF1 | 0.499 | < 0.001 |
| AC007255.1 | AC011379.2 | 0.499 | < 0.001 |
| AC007255.1 | AC010205.1 | 0.499 | < 0.001 |
| AC007255.1 | VPS13A | 0.499 | < 0.001 |
| AC007255.1 | AL591806.3 | 0.499 | < 0.001 |
| AC007255.1 | RPN2 | 0.499 | < 0.001 |
| AC007255.1 | AL360268.1 | 0.499 | < 0.001 |
| AC007255.1 | AL662797.1 | 0.498 | < 0.001 |
| AC007255.1 | AL109659.2 | 0.498 | < 0.001 |
| AC007255.1 | LINC02387 | 0.498 | < 0.001 |
| AC007255.1 | PDZK1 | 0.498 | < 0.001 |
| AC007255.1 | TNNC2 | 0.498 | < 0.001 |
| AC007255.1 | TLK2P2 | 0.498 | < 0.001 |
| AC007255.1 | CALHM3 | 0.498 | < 0.001 |
| AC007255.1 | AC009403.1 | 0.498 | < 0.001 |
| AC007255.1 | TIMM10B | 0.498 | < 0.001 |
| AC007255.1 | NRBF2P5 | 0.497 | < 0.001 |
| AC007255.1 | LINC01409 | 0.497 | < 0.001 |
| AC007255.1 | Z99127.2 | 0.497 | < 0.001 |
| AC007255.1 | ALDH1A1 | 0.497 | < 0.001 |
| AC007255.1 | CHRNA10 | 0.497 | < 0.001 |
| AC007255.1 | ZNF33B | 0.497 | < 0.001 |
| AC007255.1 | SBK3 | 0.497 | < 0.001 |
| AC007255.1 | BIRC3 | 0.497 | < 0.001 |
| AC007255.1 | AC091544.2 | 0.497 | < 0.001 |
| AC007255.1 | DNM2 | 0.497 | < 0.001 |
| AC007255.1 | NXF3 | 0.497 | < 0.001 |
| AC007255.1 | RDH5 | 0.497 | < 0.001 |
| AC007255.1 | AC002073.1 | 0.497 | < 0.001 |
| AC007255.1 | PCDHAC2 | 0.497 | < 0.001 |
| AC007255.1 | LDHAL6EP | 0.497 | < 0.001 |
| AC007255.1 | PRKCA-AS1 | 0.497 | < 0.001 |
| AC007255.1 | CD46 | 0.497 | < 0.001 |
| AC007255.1 | AL591848.2 | 0.497 | < 0.001 |
| AC007255.1 | AC015921.1 | 0.496 | < 0.001 |
| AC007255.1 | CRYM | 0.496 | < 0.001 |
| AC007255.1 | ZNF320 | 0.496 | < 0.001 |
| AC007255.1 | DIDO1 | 0.496 | < 0.001 |
| AC007255.1 | HMGA1P5 | 0.496 | < 0.001 |
| AC007255.1 | MTG2 | 0.496 | < 0.001 |
| AC007255.1 | GRK5-IT1 | 0.496 | < 0.001 |
| AC007255.1 | DGAT1 | 0.496 | < 0.001 |
| AC007255.1 | MVP | 0.496 | < 0.001 |
| AC007255.1 | MTND2P11 | 0.496 | < 0.001 |
| AC007255.1 | BTBD8 | 0.496 | < 0.001 |
| AC007255.1 | SYTL4 | 0.496 | < 0.001 |
| AC007255.1 | SLC13A2 | 0.496 | < 0.001 |
| AC007255.1 | MIR5195 | 0.496 | < 0.001 |
| AC007255.1 | EPB41L2 | 0.495 | < 0.001 |
| AC007255.1 | AL121895.2 | 0.495 | < 0.001 |
| AC007255.1 | GCNT7 | 0.495 | < 0.001 |
| AC007255.1 | FAM136A | 0.495 | < 0.001 |
| AC007255.1 | SLC35C1 | 0.495 | < 0.001 |
| AC007255.1 | CMTM2 | 0.495 | < 0.001 |
| AC007255.1 | AC010326.4 | 0.495 | < 0.001 |
| AC007255.1 | AC024580.2 | 0.495 | < 0.001 |
| AC007255.1 | PRKCD | 0.495 | < 0.001 |
| AC007255.1 | AC010761.3 | 0.495 | < 0.001 |
| AC007255.1 | SFXN2 | 0.495 | < 0.001 |
| AC007255.1 | MUC20 | 0.495 | < 0.001 |
| AC007255.1 | ACOT8 | 0.495 | < 0.001 |
| AC007255.1 | SEPSECS | 0.494 | < 0.001 |
| AC007255.1 | RILP | 0.494 | < 0.001 |
| AC007255.1 | RN7SL840P | 0.494 | < 0.001 |
| AC007255.1 | MOXD2P | 0.494 | < 0.001 |
| AC007255.1 | CLIC1 | 0.494 | < 0.001 |
| AC007255.1 | ARHGAP6 | 0.494 | < 0.001 |
| AC007255.1 | ULK4 | 0.494 | < 0.001 |
| AC007255.1 | CATIP-AS1 | 0.494 | < 0.001 |
| AC007255.1 | AC010326.5 | 0.494 | < 0.001 |
| AC007255.1 | CNPPD1 | 0.494 | < 0.001 |
| AC007255.1 | ERICH4 | 0.494 | < 0.001 |
| AC007255.1 | AC012464.1 | 0.494 | < 0.001 |
| AC007255.1 | TENT5C | 0.494 | < 0.001 |
| AC007255.1 | AL136221.1 | 0.494 | < 0.001 |
| AC007255.1 | AP000974.1 | 0.494 | < 0.001 |
| AC007255.1 | REG1B | 0.494 | < 0.001 |
| AC007255.1 | FGF18 | 0.494 | < 0.001 |
| AC007255.1 | CDHR3 | 0.494 | < 0.001 |
| AC007255.1 | LINC00672 | 0.494 | < 0.001 |
| AC007255.1 | CDK11A | 0.494 | < 0.001 |
| AC007255.1 | SLC39A14 | 0.494 | < 0.001 |
| AC007255.1 | TMEM87B | 0.494 | < 0.001 |
| AC007255.1 | SLC30A7 | 0.493 | < 0.001 |
| AC007255.1 | DGKE | 0.493 | < 0.001 |
| AC007255.1 | AC002306.1 | 0.493 | < 0.001 |
| AC007255.1 | UBE4A | 0.493 | < 0.001 |
| AC007255.1 | AC103760.1 | 0.493 | < 0.001 |
| AC007255.1 | AC008514.1 | 0.493 | < 0.001 |
| AC007255.1 | LINC00853 | 0.493 | < 0.001 |
| AC007255.1 | ASLP1 | 0.493 | < 0.001 |
| AC007255.1 | AC066613.1 | 0.493 | < 0.001 |
| AC007255.1 | BPIFB1 | 0.493 | < 0.001 |
| AC007255.1 | CCNB3 | 0.493 | < 0.001 |
| AC007255.1 | SNX9 | 0.493 | < 0.001 |
| AC007255.1 | ACO1 | 0.493 | < 0.001 |
| AC007255.1 | SLC28A2 | 0.493 | < 0.001 |
| AC007255.1 | HAVCR1 | 0.493 | < 0.001 |
| AC007255.1 | SEC16A | 0.493 | < 0.001 |
| AC007255.1 | OTC | 0.493 | < 0.001 |
| AC007255.1 | KIN | 0.492 | < 0.001 |
| AC007255.1 | SPPL2B | 0.492 | < 0.001 |
| AC007255.1 | PNMT | 0.492 | < 0.001 |
| AC007255.1 | FITM2 | 0.492 | < 0.001 |
| AC007255.1 | C5orf63 | 0.492 | < 0.001 |
| AC007255.1 | AC099518.2 | 0.492 | < 0.001 |
| AC007255.1 | SAPCD1-AS1 | 0.492 | < 0.001 |
| AC007255.1 | SLC12A7 | 0.492 | < 0.001 |
| AC007255.1 | ZNF761 | 0.492 | < 0.001 |
| AC007255.1 | NSF | 0.492 | < 0.001 |
| AC007255.1 | AC004009.3 | 0.492 | < 0.001 |
| AC007255.1 | HCG18 | 0.492 | < 0.001 |
| AC007255.1 | MUCL3 | 0.492 | < 0.001 |
| AC007255.1 | ZNF621 | 0.492 | < 0.001 |
| AC007255.1 | AC024361.1 | 0.492 | < 0.001 |
| AC007255.1 | RSRP1 | 0.492 | < 0.001 |
| AC007255.1 | CRELD1 | 0.492 | < 0.001 |
| AC007255.1 | DAPK1-IT1 | 0.492 | < 0.001 |
| AC007255.1 | AC092916.2 | 0.492 | < 0.001 |
| AC007255.1 | POU5F1 | 0.492 | < 0.001 |
| AC007255.1 | AC016405.1 | 0.492 | < 0.001 |
| AC007255.1 | AL159163.1 | 0.491 | < 0.001 |
| AC007255.1 | MIR3176 | 0.491 | < 0.001 |
| AC007255.1 | SNORD69 | 0.491 | < 0.001 |
| AC007255.1 | UBD | 0.491 | < 0.001 |
| AC007255.1 | AC098934.2 | 0.491 | < 0.001 |
| AC007255.1 | AP000697.1 | 0.491 | < 0.001 |
| AC007255.1 | DLEC1 | 0.491 | < 0.001 |
| AC007255.1 | ATP1B1 | 0.491 | < 0.001 |
| AC007255.1 | KIF5B | 0.491 | < 0.001 |
| AC007255.1 | ADH6 | 0.491 | < 0.001 |
| AC007255.1 | AC092042.1 | 0.491 | < 0.001 |
| AC007255.1 | LRCH1 | 0.491 | < 0.001 |
| AC007255.1 | AC010719.1 | 0.491 | < 0.001 |
| AC007255.1 | HNRNPA3P11 | 0.491 | < 0.001 |
| AC007255.1 | AC083843.4 | 0.491 | < 0.001 |
| AC007255.1 | AC073333.2 | 0.491 | < 0.001 |
| AC007255.1 | DAP | 0.491 | < 0.001 |
| AC007255.1 | GP2 | 0.490 | < 0.001 |
| AC007255.1 | BX470102.1 | 0.490 | < 0.001 |
| AC007255.1 | TNFRSF1B | 0.490 | < 0.001 |
| AC007255.1 | AC099509.1 | 0.490 | < 0.001 |
| AC007255.1 | MAPK8 | 0.490 | < 0.001 |
| AC007255.1 | ZNF233 | 0.490 | < 0.001 |
| AC007255.1 | DUSP8 | 0.490 | < 0.001 |
| AC007255.1 | HOXA-AS3 | 0.490 | < 0.001 |
| AC007255.1 | PRDX3 | 0.490 | < 0.001 |
| AC007255.1 | WIPF2 | 0.490 | < 0.001 |
| AC007255.1 | SNRPCP4 | 0.490 | < 0.001 |
| AC007255.1 | ALPP | 0.490 | < 0.001 |
| AC007255.1 | LINC01224 | 0.489 | < 0.001 |
| AC007255.1 | SETDB2 | 0.489 | < 0.001 |
| AC007255.1 | AC005162.2 | 0.489 | < 0.001 |
| AC007255.1 | KCNQ1OT1 | 0.489 | < 0.001 |
| AC007255.1 | TMPPE | 0.489 | < 0.001 |
| AC007255.1 | Z83841.1 | 0.489 | < 0.001 |
| AC007255.1 | ELN-AS1 | 0.489 | < 0.001 |
| AC007255.1 | DDX23 | 0.489 | < 0.001 |
| AC007255.1 | GPS2P2 | 0.489 | < 0.001 |
| AC007255.1 | AL358075.1 | 0.489 | < 0.001 |
| AC007255.1 | PRKAA1 | 0.489 | < 0.001 |
| AC007255.1 | BX255925.2 | 0.489 | < 0.001 |
| AC007255.1 | PLEKHG7 | 0.489 | < 0.001 |
| AC007255.1 | E2F8 | 0.489 | < 0.001 |
| AC007255.1 | MADD | 0.489 | < 0.001 |
| AC007255.1 | AC092902.2 | 0.489 | < 0.001 |
| AC007255.1 | RNU6-494P | 0.489 | < 0.001 |
| AC007255.1 | CEACAM16 | 0.489 | < 0.001 |
| AC007255.1 | NPAS2 | 0.489 | < 0.001 |
| AC007255.1 | AL591848.4 | 0.489 | < 0.001 |
| AC007255.1 | AGPAT2 | 0.489 | < 0.001 |
| AC007255.1 | AC068533.2 | 0.489 | < 0.001 |
| AC007255.1 | STIL | 0.489 | < 0.001 |
| AC007255.1 | RPS3AP14 | 0.488 | < 0.001 |
| AC007255.1 | SLC25A24 | 0.488 | < 0.001 |
| AC007255.1 | AC096637.1 | 0.488 | < 0.001 |
| AC007255.1 | AC068587.2 | 0.488 | < 0.001 |
| AC007255.1 | ILF2P1 | 0.488 | < 0.001 |
| AC007255.1 | RANBP17 | 0.488 | < 0.001 |
| AC007255.1 | MSANTD2 | 0.488 | < 0.001 |
| AC007255.1 | CNOT10 | 0.488 | < 0.001 |
| AC007255.1 | NR1H4 | 0.488 | < 0.001 |
| AC007255.1 | AC009093.4 | 0.488 | < 0.001 |
| AC007255.1 | ACTL10 | 0.488 | < 0.001 |
| AC007255.1 | REG3A | 0.488 | < 0.001 |
| AC007255.1 | RNF126P1 | 0.488 | < 0.001 |
| AC007255.1 | AC140479.1 | 0.488 | < 0.001 |
| AC007255.1 | ZNF789 | 0.488 | < 0.001 |
| AC007255.1 | PLA1A | 0.487 | < 0.001 |
| AC007255.1 | AC090340.1 | 0.487 | < 0.001 |
| AC007255.1 | AC024592.2 | 0.487 | < 0.001 |
| AC007255.1 | AL583722.4 | 0.487 | < 0.001 |
| AC007255.1 | FGD5-AS1 | 0.487 | < 0.001 |
| AC007255.1 | TRIM27 | 0.487 | < 0.001 |
| AC007255.1 | SPATA6 | 0.487 | < 0.001 |
| AC007255.1 | CFAP44 | 0.487 | < 0.001 |
| AC007255.1 | TMPRSS7 | 0.487 | < 0.001 |
| AC007255.1 | U73166.1 | 0.487 | < 0.001 |
| AC007255.1 | MRNIP | 0.487 | < 0.001 |
| AC007255.1 | LRRC27 | 0.487 | < 0.001 |
| AC007255.1 | PLA2G2A | 0.487 | < 0.001 |
| AC007255.1 | CNNM4 | 0.487 | < 0.001 |
| AC007255.1 | DOP1B | 0.487 | < 0.001 |
| AC007255.1 | AC010087.1 | 0.487 | < 0.001 |
| AC007255.1 | TAOK2 | 0.487 | < 0.001 |
| AC007255.1 | AL592211.1 | 0.487 | < 0.001 |
| AC007255.1 | AC024060.2 | 0.487 | < 0.001 |
| AC007255.1 | AL512444.1 | 0.486 | < 0.001 |
| AC007255.1 | AC108516.2 | 0.486 | < 0.001 |
| AC007255.1 | ZG16 | 0.486 | < 0.001 |
| AC007255.1 | LIMD1 | 0.486 | < 0.001 |
| AC007255.1 | AL512770.1 | 0.486 | < 0.001 |
| AC007255.1 | PRDM16 | 0.486 | < 0.001 |
| AC007255.1 | CYP2C19 | 0.486 | < 0.001 |
| AC007255.1 | SNORA47 | 0.486 | < 0.001 |
| AC007255.1 | PDSS1 | 0.486 | < 0.001 |
| AC007255.1 | AC006994.1 | 0.485 | < 0.001 |
| AC007255.1 | EPB41L5 | 0.485 | < 0.001 |
| AC007255.1 | VWA2 | 0.485 | < 0.001 |
| AC007255.1 | ARF4 | 0.485 | < 0.001 |
| AC007255.1 | AC145146.1 | 0.485 | < 0.001 |
| AC007255.1 | IQCH-AS1 | 0.485 | < 0.001 |
| AC007255.1 | PRSS2 | 0.485 | < 0.001 |
| AC007255.1 | AC138393.1 | 0.485 | < 0.001 |
| AC007255.1 | AGXT | 0.485 | < 0.001 |
| AC007255.1 | MAT2A | 0.485 | < 0.001 |
| AC007255.1 | MTCO3P43 | 0.485 | < 0.001 |
| AC007255.1 | RABGAP1L | 0.485 | < 0.001 |
| AC007255.1 | MIR5189 | 0.485 | < 0.001 |
| AC007255.1 | AL035405.1 | 0.485 | < 0.001 |
| AC007255.1 | LINC02018 | 0.485 | < 0.001 |
| AC007255.1 | REPIN1 | 0.485 | < 0.001 |
| AC007255.1 | AC007272.2 | 0.485 | < 0.001 |
| AC007255.1 | ZNF137P | 0.485 | < 0.001 |
| AC007255.1 | ADH1C | 0.485 | < 0.001 |
| AC007255.1 | DACH1 | 0.484 | < 0.001 |
| AC007255.1 | MSI2 | 0.484 | < 0.001 |
| AC007255.1 | AC135050.6 | 0.484 | < 0.001 |
| AC007255.1 | LINC01819 | 0.484 | < 0.001 |
| AC007255.1 | INPP5J | 0.484 | < 0.001 |
| AC007255.1 | C2-AS1 | 0.484 | < 0.001 |
| AC007255.1 | GRIN2D | 0.484 | < 0.001 |
| AC007255.1 | UBL3 | 0.484 | < 0.001 |
| AC007255.1 | AC068533.3 | 0.484 | < 0.001 |
| AC007255.1 | RPL36P16 | 0.484 | < 0.001 |
| AC007255.1 | SEMA3B-AS1 | 0.484 | < 0.001 |
| AC007255.1 | AP001372.1 | 0.484 | < 0.001 |
| AC007255.1 | AC096921.2 | 0.484 | < 0.001 |
| AC007255.1 | CHP1 | 0.484 | < 0.001 |
| AC007255.1 | TMEM106C | 0.484 | < 0.001 |
| AC007255.1 | TMTC4 | 0.484 | < 0.001 |
| AC007255.1 | KAAG1 | 0.484 | < 0.001 |
| AC007255.1 | PHBP19 | 0.483 | < 0.001 |
| AC007255.1 | AP002498.1 | 0.483 | < 0.001 |
| AC007255.1 | ZNF585A | 0.483 | < 0.001 |
| AC007255.1 | C2CD4D | 0.483 | < 0.001 |
| AC007255.1 | SEMA3F-AS1 | 0.483 | < 0.001 |
| AC007255.1 | DECR2 | 0.483 | < 0.001 |
| AC007255.1 | TMEM106A | 0.483 | < 0.001 |
| AC007255.1 | MAL2-AS1 | 0.483 | < 0.001 |
| AC007255.1 | TAS2R38 | 0.483 | < 0.001 |
| AC007255.1 | AF124730.2 | 0.483 | < 0.001 |
| AC007255.1 | CADPS | 0.483 | < 0.001 |
| AC007255.1 | ARL4AP2 | 0.483 | < 0.001 |
| AC007255.1 | STK4 | 0.483 | < 0.001 |
| AC007255.1 | RN7SL834P | 0.483 | < 0.001 |
| AC007255.1 | AL031768.1 | 0.483 | < 0.001 |
| AC007255.1 | RNFT2 | 0.483 | < 0.001 |
| AC007255.1 | CYP3A7 | 0.483 | < 0.001 |
| AC007255.1 | GC | 0.482 | < 0.001 |
| AC007255.1 | GAD1 | 0.482 | < 0.001 |
| AC007255.1 | KRT18P8 | 0.482 | < 0.001 |
| AC007255.1 | GCHFR | 0.482 | < 0.001 |
| AC007255.1 | LINC00910 | 0.482 | < 0.001 |
| AC007255.1 | HEATR5A | 0.482 | < 0.001 |
| AC007255.1 | CPVL | 0.482 | < 0.001 |
| AC007255.1 | TLE6 | 0.482 | < 0.001 |
| AC007255.1 | AC126564.1 | 0.482 | < 0.001 |
| AC007255.1 | ZNF432 | 0.482 | < 0.001 |
| AC007255.1 | AC104809.2 | 0.482 | < 0.001 |
| AC007255.1 | TRNP1 | 0.482 | < 0.001 |
| AC007255.1 | LINC00659 | 0.482 | < 0.001 |
| AC007255.1 | AC132872.3 | 0.482 | < 0.001 |
| AC007255.1 | SHROOM1 | 0.482 | < 0.001 |
| AC007255.1 | MINDY4 | 0.481 | < 0.001 |
| AC007255.1 | ACAD8 | 0.481 | < 0.001 |
| AC007255.1 | ZNF44 | 0.481 | < 0.001 |
| AC007255.1 | MAP2K6 | 0.481 | < 0.001 |
| AC007255.1 | AC092755.1 | 0.481 | < 0.001 |
| AC007255.1 | TUBG1P | 0.481 | < 0.001 |
| AC007255.1 | NCOA7 | 0.481 | < 0.001 |
| AC007255.1 | ZNF33BP1 | 0.481 | < 0.001 |
| AC007255.1 | AC019257.2 | 0.481 | < 0.001 |
| AC007255.1 | HMGB1P7 | 0.481 | < 0.001 |
| AC007255.1 | AC078962.2 | 0.481 | < 0.001 |
| AC007255.1 | AC026191.1 | 0.481 | < 0.001 |
| AC007255.1 | AC084262.1 | 0.481 | < 0.001 |
| AC007255.1 | ADD3 | 0.481 | < 0.001 |
| AC007255.1 | LCORL | 0.481 | < 0.001 |
| AC007255.1 | HIP1R | 0.481 | < 0.001 |
| AC007255.1 | DLL4 | 0.481 | < 0.001 |
| AC007255.1 | ZNF552 | 0.481 | < 0.001 |
| AC007255.1 | AC012363.2 | 0.481 | < 0.001 |
| AC007255.1 | MAST2 | 0.481 | < 0.001 |
| AC007255.1 | RNU6-704P | 0.481 | < 0.001 |
| AC007255.1 | CAT | 0.481 | < 0.001 |
| AC007255.1 | AC092171.1 | 0.481 | < 0.001 |
| AC007255.1 | ZNF708 | 0.480 | < 0.001 |
| AC007255.1 | RNU6-820P | 0.480 | < 0.001 |
| AC007255.1 | HCG27 | 0.480 | < 0.001 |
| AC007255.1 | TP53TG5 | 0.480 | < 0.001 |
| AC007255.1 | KRT8P31 | 0.480 | < 0.001 |
| AC007255.1 | C2orf88 | 0.480 | < 0.001 |
| AC007255.1 | SLAIN1 | 0.480 | < 0.001 |
| AC007255.1 | SDSL | 0.480 | < 0.001 |
| AC007255.1 | RNU1-22P | 0.480 | < 0.001 |
| AC007255.1 | CYP2C9 | 0.480 | < 0.001 |
| AC007255.1 | PEAK1 | 0.480 | < 0.001 |
| AC007255.1 | KRT8P22 | 0.480 | < 0.001 |
| AC007255.1 | KIAA1143 | 0.480 | < 0.001 |
| AC007255.1 | AF064860.2 | 0.480 | < 0.001 |
| AC007255.1 | FAM155A | 0.480 | < 0.001 |
| AC007255.1 | AC092117.2 | 0.480 | < 0.001 |
| AC007255.1 | SLC6A7 | 0.480 | < 0.001 |
| AC007255.1 | GRTP1-AS1 | 0.480 | < 0.001 |
| AC007255.1 | PP2D1 | 0.480 | < 0.001 |
| AC007255.1 | AC106037.3 | 0.480 | < 0.001 |
| AC007255.1 | AC104447.1 | 0.480 | < 0.001 |
| AC007255.1 | Z94721.1 | 0.480 | < 0.001 |
| AC007255.1 | DRICH1 | 0.480 | < 0.001 |
| AC007255.1 | RPS29P14 | 0.480 | < 0.001 |
| AC007255.1 | COTL1 | 0.479 | < 0.001 |
| AC007255.1 | AL359532.1 | 0.479 | < 0.001 |
| AC007255.1 | OXSM | 0.479 | < 0.001 |
| AC007255.1 | AC139720.2 | 0.479 | < 0.001 |
| AC007255.1 | ZNF396 | 0.479 | < 0.001 |
| AC007255.1 | HSPD1P11 | 0.479 | < 0.001 |
| AC007255.1 | AC243967.2 | 0.479 | < 0.001 |
| AC007255.1 | ZNF253 | 0.479 | < 0.001 |
| AC007255.1 | MTIF3 | 0.479 | < 0.001 |
| AC007255.1 | HCG20 | 0.479 | < 0.001 |
| AC007255.1 | GNL3 | 0.479 | < 0.001 |
| AC007255.1 | SEZ6 | 0.479 | < 0.001 |
| AC007255.1 | TUSC8 | 0.479 | < 0.001 |
| AC007255.1 | GALNT5 | 0.479 | < 0.001 |
| AC007255.1 | AC003991.2 | 0.479 | < 0.001 |
| AC007255.1 | VDAC1P11 | 0.479 | < 0.001 |
| AC007255.1 | AC022784.1 | 0.479 | < 0.001 |
| AC007255.1 | PRSS55 | 0.479 | < 0.001 |
| AC007255.1 | AL021407.3 | 0.479 | < 0.001 |
| AC007255.1 | RN7SL145P | 0.479 | < 0.001 |
| AC007255.1 | PRKAR2A | 0.479 | < 0.001 |
| AC007255.1 | AC023590.1 | 0.479 | < 0.001 |
| AC007255.1 | CASP7 | 0.478 | < 0.001 |
| AC007255.1 | BCL2L1-AS1 | 0.478 | < 0.001 |
| AC007255.1 | RHPN1 | 0.478 | < 0.001 |
| AC007255.1 | DNAJC16 | 0.478 | < 0.001 |
| AC007255.1 | MORF4L2-AS1 | 0.478 | < 0.001 |
| AC007255.1 | RAB15 | 0.478 | < 0.001 |
| AC007255.1 | TOMM34 | 0.478 | < 0.001 |
| AC007255.1 | ARIH2 | 0.478 | < 0.001 |
| AC007255.1 | ZNF503-AS1 | 0.478 | < 0.001 |
| AC007255.1 | AC008700.1 | 0.478 | < 0.001 |
| AC007255.1 | SOWAHD | 0.478 | < 0.001 |
| AC007255.1 | TGDS | 0.478 | < 0.001 |
| AC007255.1 | FAM186A | 0.478 | < 0.001 |
| AC007255.1 | AL138999.1 | 0.478 | < 0.001 |
| AC007255.1 | PHF5CP | 0.478 | < 0.001 |
| AC007255.1 | AL021920.2 | 0.477 | < 0.001 |
| AC007255.1 | AL513185.3 | 0.477 | < 0.001 |
| AC007255.1 | TMEM178A | 0.477 | < 0.001 |
| AC007255.1 | MTND4P26 | 0.477 | < 0.001 |
| AC007255.1 | AL132780.5 | 0.477 | < 0.001 |
| AC007255.1 | HMGN1P24 | 0.477 | < 0.001 |
| AC007255.1 | AC010615.1 | 0.477 | < 0.001 |
| AC007255.1 | NPFFR1 | 0.477 | < 0.001 |
| AC007255.1 | HOXB-AS2 | 0.477 | < 0.001 |
| AC007255.1 | TMEM243 | 0.477 | < 0.001 |
| AC007255.1 | PACSIN2 | 0.477 | < 0.001 |
| AC007255.1 | TTBK1 | 0.477 | < 0.001 |
| AC007255.1 | BMI1 | 0.477 | < 0.001 |
| AC007255.1 | DGAT2 | 0.477 | < 0.001 |
| AC007255.1 | TRIM2 | 0.477 | < 0.001 |
| AC007255.1 | AC020928.2 | 0.477 | < 0.001 |
| AC007255.1 | CRY1 | 0.477 | < 0.001 |
| AC007255.1 | AC018607.1 | 0.477 | < 0.001 |
| AC007255.1 | GSTO2 | 0.477 | < 0.001 |
| AC007255.1 | AC092375.1 | 0.477 | < 0.001 |
| AC007255.1 | RPS3AP39 | 0.477 | < 0.001 |
| AC007255.1 | AC104964.2 | 0.476 | < 0.001 |
| AC007255.1 | RHOF | 0.476 | < 0.001 |
| AC007255.1 | B4GALT5 | 0.476 | < 0.001 |
| AC007255.1 | AC009237.3 | 0.476 | < 0.001 |
| AC007255.1 | PEX1 | 0.476 | < 0.001 |
| AC007255.1 | AC136475.3 | 0.476 | < 0.001 |
| AC007255.1 | FAM221B | 0.476 | < 0.001 |
| AC007255.1 | IFT172 | 0.476 | < 0.001 |
| AC007255.1 | AL160314.2 | 0.476 | < 0.001 |
| AC007255.1 | AC008758.2 | 0.476 | < 0.001 |
| AC007255.1 | FBXL19-AS1 | 0.476 | < 0.001 |
| AC007255.1 | CYB561 | 0.476 | < 0.001 |
| AC007255.1 | SIM2 | 0.475 | < 0.001 |
| AC007255.1 | LINC00114 | 0.475 | < 0.001 |
| AC007255.1 | SMAGP | 0.475 | < 0.001 |
| AC007255.1 | AC009086.2 | 0.475 | < 0.001 |
| AC007255.1 | RPGR | 0.475 | < 0.001 |
| AC007255.1 | PCBD1 | 0.475 | < 0.001 |
| AC007255.1 | CCDC17 | 0.475 | < 0.001 |
| AC007255.1 | RN7SL381P | 0.475 | < 0.001 |
| AC007255.1 | C5orf52 | 0.475 | < 0.001 |
| AC007255.1 | TARDBPP2 | 0.474 | < 0.001 |
| AC007255.1 | SLC1A7 | 0.474 | < 0.001 |
| AC007255.1 | GLCCI1 | 0.474 | < 0.001 |
| AC007255.1 | IFT88 | 0.474 | < 0.001 |
| AC007255.1 | UNC13A | 0.474 | < 0.001 |
| AC007255.1 | AL591848.1 | 0.474 | < 0.001 |
| AC007255.1 | RBM39 | 0.474 | < 0.001 |
| AC007255.1 | NDUFA3P4 | 0.474 | < 0.001 |
| AC007255.1 | AC012236.1 | 0.474 | < 0.001 |
| AC007255.1 | SLC16A5 | 0.474 | < 0.001 |
| AC007255.1 | AC027290.3 | 0.474 | < 0.001 |
| AC007255.1 | FAM222A-AS1 | 0.474 | < 0.001 |
| AC007255.1 | LARP1B | 0.474 | < 0.001 |
| AC007255.1 | MTND5P28 | 0.474 | < 0.001 |
| AC007255.1 | BRPF1 | 0.474 | < 0.001 |
| AC007255.1 | PRKAR2A-AS1 | 0.474 | < 0.001 |
| AC007255.1 | AL049776.1 | 0.474 | < 0.001 |
| AC007255.1 | AC139149.1 | 0.474 | < 0.001 |
| AC007255.1 | DMXL2 | 0.474 | < 0.001 |
| AC007255.1 | ZNF664 | 0.473 | < 0.001 |
| AC007255.1 | AC087286.4 | 0.473 | < 0.001 |
| AC007255.1 | KRT8P11 | 0.473 | < 0.001 |
| AC007255.1 | AC068724.2 | 0.473 | < 0.001 |
| AC007255.1 | ABHD18 | 0.473 | < 0.001 |
| AC007255.1 | LRRC37A16P | 0.473 | < 0.001 |
| AC007255.1 | CITED1 | 0.473 | < 0.001 |
| AC007255.1 | LRCH4 | 0.473 | < 0.001 |
| AC007255.1 | UBR1 | 0.473 | < 0.001 |
| AC007255.1 | GEMIN7-AS1 | 0.473 | < 0.001 |
| AC007255.1 | HEMK1 | 0.473 | < 0.001 |
| AC007255.1 | AL590096.1 | 0.473 | < 0.001 |
| AC007255.1 | AC092919.1 | 0.473 | < 0.001 |
| AC007255.1 | ATP5F1EP1 | 0.473 | < 0.001 |
| AC007255.1 | STK31 | 0.473 | < 0.001 |
| AC007255.1 | SLC38A10 | 0.473 | < 0.001 |
| AC007255.1 | AC009237.8 | 0.473 | < 0.001 |
| AC007255.1 | SKAP2 | 0.473 | < 0.001 |
| AC007255.1 | GABRD | 0.473 | < 0.001 |
| AC007255.1 | AC090948.2 | 0.472 | < 0.001 |
| AC007255.1 | ANKRD42 | 0.472 | < 0.001 |
| AC007255.1 | LYZL4 | 0.472 | < 0.001 |
| AC007255.1 | TTLL3 | 0.472 | < 0.001 |
| AC007255.1 | BIRC7 | 0.472 | < 0.001 |
| AC007255.1 | AC114748.1 | 0.472 | < 0.001 |
| AC007255.1 | PGM2L1 | 0.472 | < 0.001 |
| AC007255.1 | SCIN | 0.472 | < 0.001 |
| AC007255.1 | AC013452.2 | 0.472 | < 0.001 |
| AC007255.1 | GPRIN2 | 0.472 | < 0.001 |
| AC007255.1 | PLCL2 | 0.472 | < 0.001 |
| AC007255.1 | KRT18P51 | 0.472 | < 0.001 |
| AC007255.1 | PDIA3P2 | 0.472 | < 0.001 |
| AC007255.1 | SLAMF6P1 | 0.472 | < 0.001 |
| AC007255.1 | AC011747.1 | 0.472 | < 0.001 |
| AC007255.1 | PNPLA7 | 0.472 | < 0.001 |
| AC007255.1 | AL121829.2 | 0.472 | < 0.001 |
| AC007255.1 | PSORS1C3 | 0.471 | < 0.001 |
| AC007255.1 | FHIT | 0.471 | < 0.001 |
| AC007255.1 | LIPT2 | 0.471 | < 0.001 |
| AC007255.1 | UGGT2 | 0.471 | < 0.001 |
| AC007255.1 | TNRC18 | 0.471 | < 0.001 |
| AC007255.1 | AC004691.1 | 0.471 | < 0.001 |
| AC007255.1 | TMEM135 | 0.471 | < 0.001 |
| AC007255.1 | ACOXL | 0.471 | < 0.001 |
| AC007255.1 | FAM3D-AS1 | 0.471 | < 0.001 |
| AC007255.1 | AP000347.1 | 0.471 | < 0.001 |
| AC007255.1 | AL139081.1 | 0.471 | < 0.001 |
| AC007255.1 | ETHE1 | 0.471 | < 0.001 |
| AC007255.1 | SPCS1 | 0.470 | < 0.001 |
| AC007255.1 | MT-ATP6 | 0.470 | < 0.001 |
| AC007255.1 | CEACAM8 | 0.470 | < 0.001 |
| AC007255.1 | TMEM30B | 0.470 | < 0.001 |
| AC007255.1 | TEC | 0.470 | < 0.001 |
| AC007255.1 | AC102945.2 | 0.470 | < 0.001 |
| AC007255.1 | MIR429 | 0.470 | < 0.001 |
| AC007255.1 | AC010538.1 | 0.470 | < 0.001 |
| AC007255.1 | PXMP4 | 0.470 | < 0.001 |
| AC007255.1 | CALM2P2 | 0.470 | < 0.001 |
| AC007255.1 | SPRY4 | 0.470 | < 0.001 |
| AC007255.1 | CALM3 | 0.470 | < 0.001 |
| AC007255.1 | AC064874.1 | 0.470 | < 0.001 |
| AC007255.1 | CPNE9 | 0.470 | < 0.001 |
| AC007255.1 | AL049697.1 | 0.470 | < 0.001 |
| AC007255.1 | KRT8P14 | 0.470 | < 0.001 |
| AC007255.1 | IL2RG | 0.470 | < 0.001 |
| AC007255.1 | AC083906.3 | 0.470 | < 0.001 |
| AC007255.1 | LINC02753 | 0.470 | < 0.001 |
| AC007255.1 | AC011477.1 | 0.470 | < 0.001 |
| AC007255.1 | SLC35D3 | 0.469 | < 0.001 |
| AC007255.1 | SATB1 | 0.469 | < 0.001 |
| AC007255.1 | ADCY4 | 0.469 | < 0.001 |
| AC007255.1 | AC011676.3 | 0.469 | < 0.001 |
| AC007255.1 | CAPS | 0.469 | < 0.001 |
| AC007255.1 | LINC00896 | 0.469 | < 0.001 |
| AC007255.1 | SLC34A2 | 0.469 | < 0.001 |
| AC007255.1 | TSC22D1-AS1 | 0.469 | < 0.001 |
| AC007255.1 | MIR1266 | 0.469 | < 0.001 |
| AC007255.1 | AC027601.5 | 0.469 | < 0.001 |
| AC007255.1 | KIAA0895 | 0.469 | < 0.001 |
| AC007255.1 | EIF2AK3 | 0.469 | < 0.001 |
| AC007255.1 | AL133215.3 | 0.469 | < 0.001 |
| AC007255.1 | ZSWIM3 | 0.469 | < 0.001 |
| AC007255.1 | 1-Mar | 0.469 | < 0.001 |
| AC007255.1 | TERT | 0.469 | < 0.001 |
| AC007255.1 | RIPPLY1 | 0.469 | < 0.001 |
| AC007255.1 | IGBP1-AS1 | 0.469 | < 0.001 |
| AC007255.1 | AC108134.1 | 0.469 | < 0.001 |
| AC007255.1 | TOMM20L | 0.469 | < 0.001 |
| AC007255.1 | YPEL2 | 0.469 | < 0.001 |
| AC007255.1 | RNA5SP440 | 0.469 | < 0.001 |
| AC007255.1 | AC079753.1 | 0.469 | < 0.001 |
| AC007255.1 | RPS29P12 | 0.468 | < 0.001 |
| AC007255.1 | AC093157.2 | 0.468 | < 0.001 |
| AC007255.1 | AC058791.1 | 0.468 | < 0.001 |
| AC007255.1 | OGG1 | 0.468 | < 0.001 |
| AC007255.1 | RNU6-1099P | 0.468 | < 0.001 |
| AC007255.1 | HPCAL1 | 0.468 | < 0.001 |
| AC007255.1 | ZNFX1 | 0.468 | < 0.001 |
| AC007255.1 | RHOA-IT1 | 0.468 | < 0.001 |
| AC007255.1 | KRT18P54 | 0.468 | < 0.001 |
| AC007255.1 | AP005233.2 | 0.468 | < 0.001 |
| AC007255.1 | FLVCR1 | 0.468 | < 0.001 |
| AC007255.1 | AC109361.2 | 0.467 | < 0.001 |
| AC007255.1 | CRB3 | 0.467 | < 0.001 |
| AC007255.1 | PRRT2 | 0.467 | < 0.001 |
| AC007255.1 | CRIP3 | 0.467 | < 0.001 |
| AC007255.1 | AL158071.4 | 0.467 | < 0.001 |
| AC007255.1 | DPH3 | 0.467 | < 0.001 |
| AC007255.1 | DDOST | 0.467 | < 0.001 |
| AC007255.1 | GNPNAT1 | 0.467 | < 0.001 |
| AC007255.1 | AC108751.5 | 0.467 | < 0.001 |
| AC007255.1 | ZNF608 | 0.467 | < 0.001 |
| AC007255.1 | AC080112.4 | 0.467 | < 0.001 |
| AC007255.1 | MSRA | 0.467 | < 0.001 |
| AC007255.1 | LINC01975 | 0.467 | < 0.001 |
| AC007255.1 | AC121336.1 | 0.467 | < 0.001 |
| AC007255.1 | GARS-DT | 0.467 | < 0.001 |
| AC007255.1 | MIR5094 | 0.467 | < 0.001 |
| AC007255.1 | AC112512.1 | 0.467 | < 0.001 |
| AC007255.1 | ABHD14B | 0.467 | < 0.001 |
| AC007255.1 | VHL | 0.467 | < 0.001 |
| AC007255.1 | NDUFB8P2 | 0.467 | < 0.001 |
| AC007255.1 | PDIA2 | 0.467 | < 0.001 |
| AC007255.1 | AURKAP2 | 0.467 | < 0.001 |
| AC007255.1 | AC023024.2 | 0.467 | < 0.001 |
| AC007255.1 | SLC35E1P1 | 0.467 | < 0.001 |
| AC007255.1 | MAP1S | 0.467 | < 0.001 |
| AC007255.1 | APOBEC2 | 0.466 | < 0.001 |
| AC007255.1 | TMEM151A | 0.466 | < 0.001 |
| AC007255.1 | MTND4P20 | 0.466 | < 0.001 |
| AC007255.1 | ZNF808 | 0.466 | < 0.001 |
| AC007255.1 | ZNF780B | 0.466 | < 0.001 |
| AC007255.1 | GAPDHP39 | 0.466 | < 0.001 |
| AC007255.1 | SNORD19C | 0.466 | < 0.001 |
| AC007255.1 | H3-4 | 0.466 | < 0.001 |
| AC007255.1 | LDLRAD1 | 0.466 | < 0.001 |
| AC007255.1 | RERE | 0.466 | < 0.001 |
| AC007255.1 | EBLN2 | 0.466 | < 0.001 |
| AC007255.1 | ACBD7 | 0.466 | < 0.001 |
| AC007255.1 | TMCO4 | 0.466 | < 0.001 |
| AC007255.1 | AL603750.1 | 0.466 | < 0.001 |
| AC007255.1 | AL583722.3 | 0.466 | < 0.001 |
| AC007255.1 | STARD10 | 0.466 | < 0.001 |
| AC007255.1 | MYH15 | 0.465 | < 0.001 |
| AC007255.1 | PIGHP1 | 0.465 | < 0.001 |
| AC007255.1 | BIN1 | 0.465 | < 0.001 |
| AC007255.1 | KRT8P48 | 0.465 | < 0.001 |
| AC007255.1 | SNORA71B | 0.465 | < 0.001 |
| AC007255.1 | ADAMTS9 | 0.465 | < 0.001 |
| AC007255.1 | EIF2AK1 | 0.465 | < 0.001 |
| AC007255.1 | AL133215.2 | 0.465 | < 0.001 |
| AC007255.1 | ZNF527 | 0.465 | < 0.001 |
| AC007255.1 | MIR196A1 | 0.465 | < 0.001 |
| AC007255.1 | RNU6-1280P | 0.465 | < 0.001 |
| AC007255.1 | COPA | 0.465 | < 0.001 |
| AC007255.1 | AL161719.1 | 0.465 | < 0.001 |
| AC007255.1 | AC008667.3 | 0.465 | < 0.001 |
| AC007255.1 | AL357033.4 | 0.465 | < 0.001 |
| AC007255.1 | AL031666.2 | 0.465 | < 0.001 |
| AC007255.1 | NR2C2 | 0.465 | < 0.001 |
| AC007255.1 | FUT6 | 0.465 | < 0.001 |
| AC007255.1 | MANEAL | 0.465 | < 0.001 |
| AC007255.1 | AL158801.5 | 0.465 | < 0.001 |
| AC007255.1 | AC008114.1 | 0.465 | < 0.001 |
| AC007255.1 | AC106772.1 | 0.465 | < 0.001 |
| AC007255.1 | RNU6-154P | 0.465 | < 0.001 |
| AC007255.1 | PPP1R15B | 0.465 | < 0.001 |
| AC007255.1 | AC083906.1 | 0.465 | < 0.001 |
| AC007255.1 | AL365181.2 | 0.465 | < 0.001 |
| AC007255.1 | AL445223.1 | 0.465 | < 0.001 |
| AC007255.1 | AC004923.2 | 0.464 | < 0.001 |
| AC007255.1 | RARRES1 | 0.464 | < 0.001 |
| AC007255.1 | PIK3R3 | 0.464 | < 0.001 |
| AC007255.1 | AL731566.2 | 0.464 | < 0.001 |
| AC007255.1 | BUD23 | 0.464 | < 0.001 |
| AC007255.1 | AC005699.1 | 0.464 | < 0.001 |
| AC007255.1 | AC010542.1 | 0.464 | < 0.001 |
| AC007255.1 | LDC1P | 0.464 | < 0.001 |
| AC007255.1 | LARGE-AS1 | 0.464 | < 0.001 |
| AC007255.1 | STS | 0.464 | < 0.001 |
| AC007255.1 | CCZ1B | 0.464 | < 0.001 |
| AC007255.1 | DNAAF4 | 0.464 | < 0.001 |
| AC007255.1 | STBD1 | 0.464 | < 0.001 |
| AC007255.1 | AC067852.3 | 0.464 | < 0.001 |
| AC007255.1 | ODC1-DT | 0.464 | < 0.001 |
| AC007255.1 | Z84492.1 | 0.464 | < 0.001 |
| AC007255.1 | RNF5 | 0.463 | < 0.001 |
| AC007255.1 | AC022080.4 | 0.463 | < 0.001 |
| AC007255.1 | AC018645.3 | 0.463 | < 0.001 |
| AC007255.1 | CDNF | 0.463 | < 0.001 |
| AC007255.1 | AL136531.3 | 0.463 | < 0.001 |
| AC007255.1 | RPL10P19 | 0.463 | < 0.001 |
| AC007255.1 | LINC00926 | 0.463 | < 0.001 |
| AC007255.1 | STRCP1 | 0.463 | < 0.001 |
| AC007255.1 | MIR215 | 0.463 | < 0.001 |
| AC007255.1 | AL133517.1 | 0.463 | < 0.001 |
| AC007255.1 | QARS1 | 0.463 | < 0.001 |
| AC007255.1 | TMEM89 | 0.463 | < 0.001 |
| AC007255.1 | PLAC4 | 0.463 | < 0.001 |
| AC007255.1 | APOA4 | 0.463 | < 0.001 |
| AC007255.1 | UCA1 | 0.463 | < 0.001 |
| AC007255.1 | DDX27 | 0.463 | < 0.001 |
| AC007255.1 | AL121601.2 | 0.463 | < 0.001 |
| AC007255.1 | CFAP221 | 0.463 | < 0.001 |
| AC007255.1 | SEC16B | 0.463 | < 0.001 |
| AC007255.1 | AC092119.2 | 0.463 | < 0.001 |
| AC007255.1 | ARRB2 | 0.463 | < 0.001 |
| AC007255.1 | AC055811.4 | 0.463 | < 0.001 |
| AC007255.1 | UPRT | 0.463 | < 0.001 |
| AC007255.1 | Z97634.1 | 0.463 | < 0.001 |
| AC007255.1 | COG3 | 0.463 | < 0.001 |
| AC007255.1 | IDH1 | 0.463 | < 0.001 |
| AC007255.1 | EPC1 | 0.463 | < 0.001 |
| AC007255.1 | GRK5 | 0.463 | < 0.001 |
| AC007255.1 | DPAGT1 | 0.463 | < 0.001 |
| AC007255.1 | NAALADL2 | 0.462 | < 0.001 |
| AC007255.1 | RPS6KA2 | 0.462 | < 0.001 |
| AC007255.1 | AC060780.3 | 0.462 | < 0.001 |
| AC007255.1 | NORAD | 0.462 | < 0.001 |
| AC007255.1 | DRD1 | 0.462 | < 0.001 |
| AC007255.1 | KLHDC4 | 0.462 | < 0.001 |
| AC007255.1 | SLC4A8 | 0.462 | < 0.001 |
| AC007255.1 | KLKB1 | 0.462 | < 0.001 |
| AC007255.1 | SELENOS | 0.462 | < 0.001 |
| AC007255.1 | ARHGAP26-AS1 | 0.462 | < 0.001 |
| AC007255.1 | MT-CO1 | 0.462 | < 0.001 |
| AC007255.1 | STEAP2 | 0.462 | < 0.001 |
| AC007255.1 | GPT | 0.462 | < 0.001 |
| AC007255.1 | SH3YL1 | 0.462 | < 0.001 |
| AC007255.1 | TMEM59 | 0.462 | < 0.001 |
| AC007255.1 | GATAD1 | 0.462 | < 0.001 |
| AC007255.1 | GNAQ | 0.462 | < 0.001 |
| AC007255.1 | MIR6784 | 0.462 | < 0.001 |
| AC007255.1 | AC005740.1 | 0.462 | < 0.001 |
| AC007255.1 | MATN1-AS1 | 0.462 | < 0.001 |
| AC007255.1 | GNA14-AS1 | 0.462 | < 0.001 |
| AC007255.1 | MFSD6L | 0.461 | < 0.001 |
| AC007255.1 | SEC11C | 0.461 | < 0.001 |
| AC007255.1 | AKR1C4 | 0.461 | < 0.001 |
| AC007255.1 | AGAP5 | 0.461 | < 0.001 |
| AC007255.1 | TRPC4AP | 0.461 | < 0.001 |
| AC007255.1 | AC005277.2 | 0.461 | < 0.001 |
| AC007255.1 | FANCF | 0.461 | < 0.001 |
| AC007255.1 | SCX | 0.461 | < 0.001 |
| AC007255.1 | KIAA1257 | 0.461 | < 0.001 |
| AC007255.1 | KIF9-AS1 | 0.461 | < 0.001 |
| AC007255.1 | UGT1A1 | 0.461 | < 0.001 |
| AC007255.1 | BNIP3P25 | 0.461 | < 0.001 |
| AC007255.1 | LINC01134 | 0.461 | < 0.001 |
| AC007255.1 | CCDC200 | 0.461 | < 0.001 |
| AC007255.1 | MSLNL | 0.461 | < 0.001 |
| AC007255.1 | AL022316.1 | 0.460 | < 0.001 |
| AC007255.1 | ANXA3 | 0.460 | < 0.001 |
| AC007255.1 | ERCC5 | 0.460 | < 0.001 |
| AC007255.1 | GALNTL6 | 0.460 | < 0.001 |
| AC007255.1 | ANKS6 | 0.460 | < 0.001 |
| AC007255.1 | G6PC3 | 0.460 | < 0.001 |
| AC007255.1 | AL078587.1 | 0.460 | < 0.001 |
| AC007255.1 | ZNF32-AS2 | 0.460 | < 0.001 |
| AC007255.1 | AC103746.1 | 0.460 | < 0.001 |
| AC007255.1 | ABCD1 | 0.460 | < 0.001 |
| AC007255.1 | HNRNPA1P59 | 0.460 | < 0.001 |
| AC007255.1 | LINC01738 | 0.460 | < 0.001 |
| AC007255.1 | GCNA | 0.460 | < 0.001 |
| AC007255.1 | REG3G | 0.460 | < 0.001 |
| AC007255.1 | KRT8P39 | 0.460 | < 0.001 |
| AC007255.1 | AL359715.2 | 0.460 | < 0.001 |
| AC007255.1 | AL138807.1 | 0.460 | < 0.001 |
| AC007255.1 | AP003555.2 | 0.460 | < 0.001 |
| AC007255.1 | AL512303.1 | 0.460 | < 0.001 |
| AC007255.1 | TTLL4 | 0.459 | < 0.001 |
| AC007255.1 | AC133961.1 | 0.459 | < 0.001 |
| AC007255.1 | AC244093.4 | 0.459 | < 0.001 |
| AC007255.1 | SLC22A5 | 0.459 | < 0.001 |
| AC007255.1 | IQCD | 0.459 | < 0.001 |
| AC007255.1 | AL035252.4 | 0.459 | < 0.001 |
| AC007255.1 | PPM1D | 0.459 | < 0.001 |
| AC007255.1 | TOB2 | 0.459 | < 0.001 |
| AC007255.1 | CPHL1P | 0.459 | < 0.001 |
| AC007255.1 | SNTB1 | 0.459 | < 0.001 |
| AC007255.1 | NUTM1 | 0.459 | < 0.001 |
| AC007255.1 | ACADSB | 0.459 | < 0.001 |
| AC007255.1 | AL158206.1 | 0.459 | < 0.001 |
| AC007255.1 | IL23R | 0.459 | < 0.001 |
| AC007255.1 | AKNA | 0.459 | < 0.001 |
| AC007255.1 | SLC22A18AS | 0.459 | < 0.001 |
| AC007255.1 | MICAL1 | 0.459 | < 0.001 |
| AC007255.1 | AC113143.1 | 0.459 | < 0.001 |
| AC007255.1 | AL583810.1 | 0.459 | < 0.001 |
| AC007255.1 | RN7SL125P | 0.458 | < 0.001 |
| AC007255.1 | TTC31 | 0.458 | < 0.001 |
| AC007255.1 | VAT1L | 0.458 | < 0.001 |
| AC007255.1 | DPEP1 | 0.458 | < 0.001 |
| AC007255.1 | GRIN2B | 0.458 | < 0.001 |
| AC007255.1 | AL049836.1 | 0.458 | < 0.001 |
| AC007255.1 | RNU6-610P | 0.458 | < 0.001 |
| AC007255.1 | NARS1P2 | 0.458 | < 0.001 |
| AC007255.1 | AC007566.1 | 0.458 | < 0.001 |
| AC007255.1 | AL158068.1 | 0.458 | < 0.001 |
| AC007255.1 | AFDN | 0.458 | < 0.001 |
| AC007255.1 | AC010542.6 | 0.458 | < 0.001 |
| AC007255.1 | AC093495.1 | 0.458 | < 0.001 |
| AC007255.1 | AC061975.7 | 0.458 | < 0.001 |
| AC007255.1 | OGT | 0.458 | < 0.001 |
| AC007255.1 | BIK | 0.458 | < 0.001 |
| AC007255.1 | ZNF417 | 0.458 | < 0.001 |
| AC007255.1 | TREH | 0.457 | < 0.001 |
| AC007255.1 | AP002992.1 | 0.457 | < 0.001 |
| AC007255.1 | DAPK2 | 0.457 | < 0.001 |
| AC007255.1 | AL022238.2 | 0.457 | < 0.001 |
| AC007255.1 | CCDC170 | 0.457 | < 0.001 |
| AC007255.1 | RNU5B-2P | 0.457 | < 0.001 |
| AC007255.1 | AL357140.2 | 0.457 | < 0.001 |
| AC007255.1 | DENND2D | 0.457 | < 0.001 |
| AC007255.1 | AC012615.4 | 0.457 | < 0.001 |
| AC007255.1 | SPATA32 | 0.457 | < 0.001 |
| AC007255.1 | ABCD4 | 0.457 | < 0.001 |
| AC007255.1 | XPC | 0.457 | < 0.001 |
| AC007255.1 | SCARNA6 | 0.457 | < 0.001 |
| AC007255.1 | AL139120.1 | 0.457 | < 0.001 |
| AC007255.1 | AL512637.1 | 0.457 | < 0.001 |
| AC007255.1 | AL583824.1 | 0.457 | < 0.001 |
| AC007255.1 | UVSSA | 0.457 | < 0.001 |
| AC007255.1 | THEM6 | 0.457 | < 0.001 |
| AC007255.1 | APEH | 0.457 | < 0.001 |
| AC007255.1 | TRAM1 | 0.457 | < 0.001 |
| AC007255.1 | TOB1-AS1 | 0.457 | < 0.001 |
| AC007255.1 | RNU6-377P | 0.456 | < 0.001 |
| AC007255.1 | AC009163.3 | 0.456 | < 0.001 |
| AC007255.1 | ENTPD6 | 0.456 | < 0.001 |
| AC007255.1 | TMEM182 | 0.456 | < 0.001 |
| AC007255.1 | ZNF506 | 0.456 | < 0.001 |
| AC007255.1 | MGAT5 | 0.456 | < 0.001 |
| AC007255.1 | AL118511.2 | 0.456 | < 0.001 |
| AC007255.1 | SLC23A3 | 0.456 | < 0.001 |
| AC007255.1 | LRRC2 | 0.456 | < 0.001 |
| AC007255.1 | AK3P5 | 0.456 | < 0.001 |
| AC007255.1 | LAMTOR5-AS1 | 0.456 | < 0.001 |
| AC007255.1 | ADRA2A | 0.456 | < 0.001 |
| AC007255.1 | DNTTIP1 | 0.456 | < 0.001 |
| AC007255.1 | AL021408.1 | 0.456 | < 0.001 |
| AC007255.1 | RNU6-251P | 0.456 | < 0.001 |
| AC007255.1 | MOAP1 | 0.456 | < 0.001 |
| AC007255.1 | MIR5587 | 0.456 | < 0.001 |
| AC007255.1 | AC004263.1 | 0.456 | < 0.001 |
| AC007255.1 | RAVER2 | 0.456 | < 0.001 |
| AC007255.1 | HSD17B7 | 0.456 | < 0.001 |
| AC007255.1 | RNA5SP277 | 0.456 | < 0.001 |
| AC007255.1 | AC087491.1 | 0.456 | < 0.001 |
| AC007255.1 | CA7 | 0.456 | < 0.001 |
| AC007255.1 | SOX30 | 0.456 | < 0.001 |
| AC007255.1 | WNT8B | 0.456 | < 0.001 |
| AC007255.1 | AL137013.1 | 0.456 | < 0.001 |
| AC007255.1 | NBR1 | 0.456 | < 0.001 |
| AC007255.1 | SPG11 | 0.456 | < 0.001 |
| AC007255.1 | TMEM205 | 0.456 | < 0.001 |
| AC007255.1 | IDNK | 0.455 | < 0.001 |
| AC007255.1 | DUSP28 | 0.455 | < 0.001 |
| AC007255.1 | TNFRSF10C | 0.455 | < 0.001 |
| AC007255.1 | GULP1 | 0.455 | < 0.001 |
| AC007255.1 | SLC22A18 | 0.455 | < 0.001 |
| AC007255.1 | AC092127.1 | 0.455 | < 0.001 |
| AC007255.1 | AC009779.4 | 0.455 | < 0.001 |
| AC007255.1 | AL139384.2 | 0.455 | < 0.001 |
| AC007255.1 | AC011676.2 | 0.455 | < 0.001 |
| AC007255.1 | LINC01359 | 0.455 | < 0.001 |
| AC007255.1 | AC100843.2 | 0.455 | < 0.001 |
| AC007255.1 | HEXB | 0.455 | < 0.001 |
| AC007255.1 | SLC51B | 0.455 | < 0.001 |
| AC007255.1 | OR7E85P | 0.455 | < 0.001 |
| AC007255.1 | HPGD | 0.455 | < 0.001 |
| AC007255.1 | AL450124.1 | 0.454 | < 0.001 |
| AC007255.1 | ARHGEF16 | 0.454 | < 0.001 |
| AC007255.1 | MIR3142HG | 0.454 | < 0.001 |
| AC007255.1 | AL157392.3 | 0.454 | < 0.001 |
| AC007255.1 | WDFY1 | 0.454 | < 0.001 |
| AC007255.1 | LRP6 | 0.454 | < 0.001 |
| AC007255.1 | ACVR1B | 0.454 | < 0.001 |
| AC007255.1 | LGR4-AS1 | 0.454 | < 0.001 |
| AC007255.1 | RNPC3 | 0.454 | < 0.001 |
| AC007255.1 | AC091179.4 | 0.454 | < 0.001 |
| AC007255.1 | ZNF721 | 0.454 | < 0.001 |
| AC007255.1 | LINGO4 | 0.454 | < 0.001 |
| AC007255.1 | KLRG1 | 0.454 | < 0.001 |
| AC007255.1 | AP001972.1 | 0.454 | < 0.001 |
| AC007255.1 | WFDC2 | 0.454 | < 0.001 |
| AC007255.1 | DXO | 0.454 | < 0.001 |
| AC007255.1 | LETMD1 | 0.454 | < 0.001 |
| AC007255.1 | INF2 | 0.454 | < 0.001 |
| AC007255.1 | CEACAM20 | 0.454 | < 0.001 |
| AC007255.1 | HS3ST1 | 0.454 | < 0.001 |
| AC007255.1 | JAGN1 | 0.454 | < 0.001 |
| AC007255.1 | AL357078.2 | 0.454 | < 0.001 |
| AC007255.1 | SLC25A53 | 0.454 | < 0.001 |
| AC007255.1 | MYEOV | 0.453 | < 0.001 |
| AC007255.1 | MEP1AP4 | 0.453 | < 0.001 |
| AC007255.1 | AC002316.1 | 0.453 | < 0.001 |
| AC007255.1 | MIR135B | 0.453 | < 0.001 |
| AC007255.1 | TLCD2 | 0.453 | < 0.001 |
| AC007255.1 | SPAG4 | 0.453 | < 0.001 |
| AC007255.1 | GMCL1P2 | 0.453 | < 0.001 |
| AC007255.1 | MOGAT1 | 0.453 | < 0.001 |
| AC007255.1 | LINC02701 | 0.453 | < 0.001 |
| AC007255.1 | LINC01356 | 0.453 | < 0.001 |
| AC007255.1 | DRGX | 0.453 | < 0.001 |
| AC007255.1 | HM13-IT1 | 0.453 | < 0.001 |
| AC007255.1 | ST6GAL1 | 0.453 | < 0.001 |
| AC007255.1 | MLH3 | 0.453 | < 0.001 |
| AC007255.1 | CCNG1P1 | 0.453 | < 0.001 |
| AC007255.1 | ZSCAN20 | 0.453 | < 0.001 |
| AC007255.1 | RN7SL204P | 0.453 | < 0.001 |
| AC007255.1 | LIPG | 0.453 | < 0.001 |
| AC007255.1 | PIK3CB | 0.453 | < 0.001 |
| AC007255.1 | AC008750.5 | 0.453 | < 0.001 |
| AC007255.1 | ZNF611 | 0.453 | < 0.001 |
| AC007255.1 | AC092902.1 | 0.453 | < 0.001 |
| AC007255.1 | CCNJ | 0.453 | < 0.001 |
| AC007255.1 | FUT9 | 0.453 | < 0.001 |
| AC007255.1 | APOOL | 0.453 | < 0.001 |
| AC007255.1 | SESTD1 | 0.453 | < 0.001 |
| AC007255.1 | TBX10 | 0.452 | < 0.001 |
| AC007255.1 | AL356740.2 | 0.452 | < 0.001 |
| AC007255.1 | AC107884.2 | 0.452 | < 0.001 |
| AC007255.1 | LINC01597 | 0.452 | < 0.001 |
| AC007255.1 | ZNF841 | 0.452 | < 0.001 |
| AC007255.1 | CIAO1 | 0.452 | < 0.001 |
| AC007255.1 | AL357134.1 | 0.452 | < 0.001 |
| AC007255.1 | ZNF37BP | 0.452 | < 0.001 |
| AC007255.1 | ARGLU1 | 0.452 | < 0.001 |
| AC007255.1 | TCIM | 0.452 | < 0.001 |
| AC007255.1 | GLB1L | 0.452 | < 0.001 |
| AC007255.1 | MRPS31P5 | 0.452 | < 0.001 |
| AC007255.1 | ENGASE | 0.452 | < 0.001 |
| AC007255.1 | JTB | 0.452 | < 0.001 |
| AC007255.1 | GOSR1 | 0.452 | < 0.001 |
| AC007255.1 | AC002064.2 | 0.452 | < 0.001 |
| AC007255.1 | MYRIP | 0.452 | < 0.001 |
| AC007255.1 | PTCHD3P2 | 0.451 | < 0.001 |
| AC007255.1 | MLLT6 | 0.451 | < 0.001 |
| AC007255.1 | AC005154.2 | 0.451 | < 0.001 |
| AC007255.1 | SPATA25 | 0.451 | < 0.001 |
| AC007255.1 | C15orf48 | 0.451 | < 0.001 |
| AC007255.1 | GUCA2B | 0.451 | < 0.001 |
| AC007255.1 | PPIP5K1 | 0.451 | < 0.001 |
| AC007255.1 | PAX4 | 0.451 | < 0.001 |
| AC007255.1 | EEF1AKNMT | 0.451 | < 0.001 |
| AC007255.1 | AC007842.1 | 0.451 | < 0.001 |
| AC007255.1 | CEACAM1 | 0.451 | < 0.001 |
| AC007255.1 | HSPA14 | 0.451 | < 0.001 |
| AC007255.1 | TOP1 | 0.451 | < 0.001 |
| AC007255.1 | CHRNA7 | 0.451 | < 0.001 |
| AC007255.1 | ADGRG6 | 0.451 | < 0.001 |
| AC007255.1 | AC015908.3 | 0.451 | < 0.001 |
| AC007255.1 | AP001610.3 | 0.450 | < 0.001 |
| AC007255.1 | BRCC3P1 | 0.450 | < 0.001 |
| AC007255.1 | MTHFR | 0.450 | < 0.001 |
| AC007255.1 | HDDC3 | 0.450 | < 0.001 |
| AC007255.1 | LINC00654 | 0.450 | < 0.001 |
| AC007255.1 | RBM15-AS1 | 0.450 | < 0.001 |
| AC007255.1 | AGFG2 | 0.450 | < 0.001 |
| AC007255.1 | AC104805.1 | 0.450 | < 0.001 |
| AC007255.1 | SSR4 | 0.450 | < 0.001 |
| AC007255.1 | TBRG1 | 0.450 | < 0.001 |
| AC007255.1 | EZR | 0.450 | < 0.001 |
| AC007255.1 | AL590133.1 | 0.450 | < 0.001 |
| AC007255.1 | AC005726.2 | 0.450 | < 0.001 |
| AC007255.1 | ENTPD2 | 0.450 | < 0.001 |
| AC007255.1 | RPL7P39 | 0.450 | < 0.001 |
| AC007255.1 | CDK5RAP1 | 0.450 | < 0.001 |
| AC007255.1 | AC087884.1 | 0.450 | < 0.001 |
| AC007255.1 | ELMOD3 | 0.450 | < 0.001 |
| AC007255.1 | ALG1 | 0.450 | < 0.001 |
| AC007255.1 | FOSL1P1 | 0.450 | < 0.001 |
| AC007255.1 | PTPRG | 0.450 | < 0.001 |
| AC007255.1 | PECR | 0.450 | < 0.001 |
| AC007255.1 | AC005332.3 | 0.450 | < 0.001 |
| AC007255.1 | CA4 | 0.449 | < 0.001 |
| AC007255.1 | MIR4676 | 0.449 | < 0.001 |
| AC007255.1 | KCNH8 | 0.449 | < 0.001 |
| AC007255.1 | PCTP | 0.449 | < 0.001 |
| AC007255.1 | AC019117.2 | 0.449 | < 0.001 |
| AC007255.1 | RBP4 | 0.449 | < 0.001 |
| AC007255.1 | ZNF891 | 0.449 | < 0.001 |
| AC007255.1 | SLC38A11 | 0.449 | < 0.001 |
| AC007255.1 | AMELX | 0.449 | < 0.001 |
| AC007255.1 | AL031668.1 | 0.449 | < 0.001 |
| AC007255.1 | OCEL1 | 0.449 | < 0.001 |
| AC007255.1 | SCLY | 0.449 | < 0.001 |
| AC007255.1 | AC022784.6 | 0.449 | < 0.001 |
| AC007255.1 | PRSS12 | 0.449 | < 0.001 |
| AC007255.1 | NPDC1 | 0.449 | < 0.001 |
| AC007255.1 | AC018926.1 | 0.449 | < 0.001 |
| AC007255.1 | TLCD4 | 0.449 | < 0.001 |
| AC007255.1 | ANO9 | 0.449 | < 0.001 |
| AC007255.1 | AC068792.1 | 0.448 | < 0.001 |
| AC007255.1 | NHLRC2 | 0.448 | < 0.001 |
| AC007255.1 | OTUD6B-AS1 | 0.448 | < 0.001 |
| AC007255.1 | ALG13 | 0.448 | < 0.001 |
| AC007255.1 | FDX1 | 0.448 | < 0.001 |
| AC007255.1 | NDUFB1P2 | 0.448 | < 0.001 |
| AC007255.1 | PTPRJ-AS1 | 0.448 | < 0.001 |
| AC007255.1 | PLXNA3 | 0.448 | < 0.001 |
| AC007255.1 | WDR55 | 0.448 | < 0.001 |
| AC007255.1 | EXOG | 0.448 | < 0.001 |
| AC007255.1 | AC011290.2 | 0.448 | < 0.001 |
| AC007255.1 | HMGB2P1 | 0.448 | < 0.001 |
| AC007255.1 | AC074124.1 | 0.448 | < 0.001 |
| AC007255.1 | AL353689.1 | 0.448 | < 0.001 |
| AC007255.1 | SUCO | 0.448 | < 0.001 |
| AC007255.1 | LRRC37A9P | 0.448 | < 0.001 |
| AC007255.1 | MIR6761 | 0.448 | < 0.001 |
| AC007255.1 | PIAS1 | 0.448 | < 0.001 |
| AC007255.1 | AC124067.3 | 0.448 | < 0.001 |
| AC007255.1 | TAF1 | 0.448 | < 0.001 |
| AC007255.1 | NSMCE4A | 0.448 | < 0.001 |
| AC007255.1 | AL355076.1 | 0.448 | < 0.001 |
| AC007255.1 | SLC19A2 | 0.447 | < 0.001 |
| AC007255.1 | AL359697.1 | 0.447 | < 0.001 |
| AC007255.1 | EXOC3L4 | 0.447 | < 0.001 |
| AC007255.1 | AC020917.4 | 0.447 | < 0.001 |
| AC007255.1 | AC097359.2 | 0.447 | < 0.001 |
| AC007255.1 | CMBL | 0.447 | < 0.001 |
| AC007255.1 | AC093535.1 | 0.447 | < 0.001 |
| AC007255.1 | SORT1 | 0.447 | < 0.001 |
| AC007255.1 | NEIL1 | 0.447 | < 0.001 |
| AC007255.1 | VSTM5 | 0.447 | < 0.001 |
| AC007255.1 | ZFAND4 | 0.447 | < 0.001 |
| AC007255.1 | AC105910.1 | 0.447 | < 0.001 |
| AC007255.1 | ZNF224 | 0.447 | < 0.001 |
| AC007255.1 | SGMS1-AS1 | 0.447 | < 0.001 |
| AC007255.1 | RNU6-944P | 0.447 | < 0.001 |
| AC007255.1 | LINC00265 | 0.446 | < 0.001 |
| AC007255.1 | AC068299.1 | 0.446 | < 0.001 |
| AC007255.1 | CLASP2 | 0.446 | < 0.001 |
| AC007255.1 | RAD17P2 | 0.446 | < 0.001 |
| AC007255.1 | ARHGEF39 | 0.446 | < 0.001 |
| AC007255.1 | STX1A | 0.446 | < 0.001 |
| AC007255.1 | MBNL3 | 0.446 | < 0.001 |
| AC007255.1 | PIK3C2A | 0.446 | < 0.001 |
| AC007255.1 | ATP6V0E1P2 | 0.446 | < 0.001 |
| AC007255.1 | AC106037.2 | 0.446 | < 0.001 |
| AC007255.1 | SYNGR4 | 0.446 | < 0.001 |
| AC007255.1 | SRPK3 | 0.446 | < 0.001 |
| AC007255.1 | SMIM5 | 0.446 | < 0.001 |
| AC007255.1 | AC022306.2 | 0.446 | < 0.001 |
| AC007255.1 | AC139720.1 | 0.446 | < 0.001 |
| AC007255.1 | YRDCP1 | 0.446 | < 0.001 |
| AC007255.1 | AC132219.2 | 0.446 | < 0.001 |
| AC007255.1 | C6 | 0.446 | < 0.001 |
| AC007255.1 | AP5Z1 | 0.446 | < 0.001 |
| AC007255.1 | AC005840.3 | 0.446 | < 0.001 |
| AC007255.1 | AC109583.3 | 0.446 | < 0.001 |
| AC007255.1 | C1orf105 | 0.446 | < 0.001 |
| AC007255.1 | AC013275.1 | 0.446 | < 0.001 |
| AC007255.1 | AC021739.5 | 0.446 | < 0.001 |
| AC007255.1 | AL355870.1 | 0.446 | < 0.001 |
| AC007255.1 | AL354696.1 | 0.445 | < 0.001 |
| AC007255.1 | ZBED3-AS1 | 0.445 | < 0.001 |
| AC007255.1 | SLC31A1 | 0.445 | < 0.001 |
| AC007255.1 | AL132780.1 | 0.445 | < 0.001 |
| AC007255.1 | PABPC1L | 0.445 | < 0.001 |
| AC007255.1 | AL390879.1 | 0.445 | < 0.001 |
| AC007255.1 | RNU6-1010P | 0.445 | < 0.001 |
| AC007255.1 | AC107993.1 | 0.445 | < 0.001 |
| AC007255.1 | BECN1 | 0.445 | < 0.001 |
| AC007255.1 | HDAC1 | 0.445 | < 0.001 |
| AC007255.1 | AC005532.1 | 0.445 | < 0.001 |
| AC007255.1 | MRPL35P2 | 0.445 | < 0.001 |
| AC007255.1 | NAPEPLD | 0.445 | < 0.001 |
| AC007255.1 | FZD4 | 0.445 | < 0.001 |
| AC007255.1 | ABTB2 | 0.445 | < 0.001 |
| AC007255.1 | RPS3AP34 | 0.445 | < 0.001 |
| AC007255.1 | AC008543.3 | 0.445 | < 0.001 |
| AC007255.1 | ZNF564 | 0.445 | < 0.001 |
| AC007255.1 | AC009237.15 | 0.444 | < 0.001 |
| AC007255.1 | AC021483.2 | 0.444 | < 0.001 |
| AC007255.1 | RDM1 | 0.444 | < 0.001 |
| AC007255.1 | XBP1P1 | 0.444 | < 0.001 |
| AC007255.1 | RNU6-73P | 0.444 | < 0.001 |
| AC007255.1 | AL031726.1 | 0.444 | < 0.001 |
| AC007255.1 | CYTH2 | 0.444 | < 0.001 |
| AC007255.1 | AC025252.3 | 0.444 | < 0.001 |
| AC007255.1 | LINC02086 | 0.444 | < 0.001 |
| AC007255.1 | SLC35E2B | 0.444 | < 0.001 |
| AC007255.1 | C4orf33 | 0.444 | < 0.001 |
| AC007255.1 | AC010132.4 | 0.444 | < 0.001 |
| AC007255.1 | AC022080.3 | 0.444 | < 0.001 |
| AC007255.1 | AC099778.1 | 0.444 | < 0.001 |
| AC007255.1 | SNX8 | 0.444 | < 0.001 |
| AC007255.1 | POLD3 | 0.444 | < 0.001 |
| AC007255.1 | PODXL2 | 0.444 | < 0.001 |
| AC007255.1 | AC018665.1 | 0.444 | < 0.001 |
| AC007255.1 | AL731567.1 | 0.444 | < 0.001 |
| AC007255.1 | C3P1 | 0.444 | < 0.001 |
| AC007255.1 | AC012150.2 | 0.444 | < 0.001 |
| AC007255.1 | SIDT1-AS1 | 0.444 | < 0.001 |
| AC007255.1 | AL031665.1 | 0.444 | < 0.001 |
| AC007255.1 | TPMT | 0.444 | < 0.001 |
| AC007255.1 | CRBN | 0.444 | < 0.001 |
| AC007255.1 | LBR | 0.444 | < 0.001 |
| AC007255.1 | SUCLG2P2 | 0.443 | < 0.001 |
| AC007255.1 | FAM122B | 0.443 | < 0.001 |
| AC007255.1 | AL359715.4 | 0.443 | < 0.001 |
| AC007255.1 | FAAH2 | 0.443 | < 0.001 |
| AC007255.1 | KRT8P34 | 0.443 | < 0.001 |
| AC007255.1 | XPNPEP1 | 0.443 | < 0.001 |
| AC007255.1 | ZNF226 | 0.443 | < 0.001 |
| AC007255.1 | TMEM72 | 0.443 | < 0.001 |
| AC007255.1 | ATAT1 | 0.443 | < 0.001 |
| AC007255.1 | AC007405.3 | 0.443 | < 0.001 |
| AC007255.1 | AC108134.3 | 0.443 | < 0.001 |
| AC007255.1 | RGS7BP | 0.443 | < 0.001 |
| AC007255.1 | HOXB8 | 0.443 | < 0.001 |
| AC007255.1 | TNFRSF10D | 0.443 | < 0.001 |
| AC007255.1 | AL121820.2 | 0.443 | < 0.001 |
| AC007255.1 | LRP5L | 0.443 | < 0.001 |
| AC007255.1 | UPF2 | 0.442 | < 0.001 |
| AC007255.1 | CDK12 | 0.442 | < 0.001 |
| AC007255.1 | FRZB | 0.442 | < 0.001 |
| AC007255.1 | AP000346.1 | 0.442 | < 0.001 |
| AC007255.1 | LINC02718 | 0.442 | < 0.001 |
| AC007255.1 | AP002812.3 | 0.442 | < 0.001 |
| AC007255.1 | AC022400.2 | 0.442 | < 0.001 |
| AC007255.1 | AC007285.2 | 0.442 | < 0.001 |
| AC007255.1 | DCAF11 | 0.442 | < 0.001 |
| AC007255.1 | SLFN13 | 0.442 | < 0.001 |
| AC007255.1 | CPNE5 | 0.442 | < 0.001 |
| AC007255.1 | LINC02863 | 0.442 | < 0.001 |
| AC007255.1 | RGN | 0.442 | < 0.001 |
| AC007255.1 | RPSAP9 | 0.442 | < 0.001 |
| AC007255.1 | MIR1972-1 | 0.442 | < 0.001 |
| AC007255.1 | MTCO2P11 | 0.442 | < 0.001 |
| AC007255.1 | SYAP1 | 0.442 | < 0.001 |
| AC007255.1 | SCYL3 | 0.442 | < 0.001 |
| AC007255.1 | TRAK2 | 0.442 | < 0.001 |
| AC007255.1 | AL121944.2 | 0.442 | < 0.001 |
| AC007255.1 | LINC00674 | 0.442 | < 0.001 |
| AC007255.1 | CLINT1 | 0.442 | < 0.001 |
| AC007255.1 | C3orf52 | 0.441 | < 0.001 |
| AC007255.1 | AC025647.3 | 0.441 | < 0.001 |
| AC007255.1 | CIB1 | 0.441 | < 0.001 |
| AC007255.1 | OSBPL3 | 0.441 | < 0.001 |
| AC007255.1 | MTATP6P1 | 0.441 | < 0.001 |
| AC007255.1 | ELL3 | 0.441 | < 0.001 |
| AC007255.1 | ZNF41 | 0.441 | < 0.001 |
| AC007255.1 | AC092868.3 | 0.441 | < 0.001 |
| AC007255.1 | BPIFA2 | 0.441 | < 0.001 |
| AC007255.1 | AC021739.4 | 0.441 | < 0.001 |
| AC007255.1 | AF124730.1 | 0.441 | < 0.001 |
| AC007255.1 | STX18-IT1 | 0.441 | < 0.001 |
| AC007255.1 | AL392172.1 | 0.441 | < 0.001 |
| AC007255.1 | ILDR1 | 0.441 | < 0.001 |
| AC007255.1 | NFATC2IP | 0.441 | < 0.001 |
| AC007255.1 | AC022150.2 | 0.441 | < 0.001 |
| AC007255.1 | SEC13 | 0.441 | < 0.001 |
| AC007255.1 | NUDT16 | 0.441 | < 0.001 |
| AC007255.1 | AC015849.3 | 0.441 | < 0.001 |
| AC007255.1 | AGMO | 0.441 | < 0.001 |
| AC007255.1 | NIPA2 | 0.441 | < 0.001 |
| AC007255.1 | AC015883.1 | 0.441 | < 0.001 |
| AC007255.1 | ACOX1 | 0.441 | < 0.001 |
| AC007255.1 | IGHV5-78 | 0.440 | < 0.001 |
| AC007255.1 | AL662844.3 | 0.440 | < 0.001 |
| AC007255.1 | ZDHHC20 | 0.440 | < 0.001 |
| AC007255.1 | NAAA | 0.440 | < 0.001 |
| AC007255.1 | ODAM | 0.440 | < 0.001 |
| AC007255.1 | THUMPD3 | 0.440 | < 0.001 |
| AC007255.1 | AL357874.2 | 0.440 | < 0.001 |
| AC007255.1 | THRB-AS1 | 0.440 | < 0.001 |
| AC007255.1 | KRT18P48 | 0.440 | < 0.001 |
| AC007255.1 | AC005332.6 | 0.440 | < 0.001 |
| AC007255.1 | AC021739.3 | 0.440 | < 0.001 |
| AC007255.1 | ZKSCAN2-DT | 0.440 | < 0.001 |
| AC007255.1 | HNRNPF | 0.440 | < 0.001 |
| AC007255.1 | GDAP2 | 0.440 | < 0.001 |
| AC007255.1 | ZSCAN12P1 | 0.440 | < 0.001 |
| AC007255.1 | C9orf64 | 0.440 | < 0.001 |
| AC007255.1 | KRT8P40 | 0.440 | < 0.001 |
| AC007255.1 | AC032044.1 | 0.440 | < 0.001 |
| AC007255.1 | ZNF573 | 0.440 | < 0.001 |
| AC007255.1 | RNU6-1102P | 0.440 | < 0.001 |
| AC007255.1 | CERS2 | 0.440 | < 0.001 |
| AC007255.1 | RGS5 | 0.440 | < 0.001 |
| AC007255.1 | AC010203.2 | 0.440 | < 0.001 |
| AC007255.1 | RN7SL589P | 0.440 | < 0.001 |
| AC007255.1 | SPAG1 | 0.440 | < 0.001 |
| AC007255.1 | AC020779.2 | 0.440 | < 0.001 |
| AC007255.1 | ACER2 | 0.440 | < 0.001 |
| AC007255.1 | MCF2L-AS1 | 0.439 | < 0.001 |
| AC007255.1 | SMC5 | 0.439 | < 0.001 |
| AC007255.1 | RPP38-DT | 0.439 | < 0.001 |
| AC007255.1 | AL110115.1 | 0.439 | < 0.001 |
| AC007255.1 | AQP12A | 0.439 | < 0.001 |
| AC007255.1 | SNORA71C | 0.439 | < 0.001 |
| AC007255.1 | CCDC126 | 0.439 | < 0.001 |
| AC007255.1 | STYK1 | 0.439 | < 0.001 |
| AC007255.1 | AC068580.2 | 0.439 | < 0.001 |
| AC007255.1 | PLCB4 | 0.439 | < 0.001 |
| AC007255.1 | RAPGEF2 | 0.439 | < 0.001 |
| AC007255.1 | IDUA | 0.439 | < 0.001 |
| AC007255.1 | AL035420.2 | 0.439 | < 0.001 |
| AC007255.1 | RPL18P10 | 0.439 | < 0.001 |
| AC007255.1 | TMEM233 | 0.439 | < 0.001 |
| AC007255.1 | TINAG | 0.439 | < 0.001 |
| AC007255.1 | TLE1P1 | 0.439 | < 0.001 |
| AC007255.1 | SKAP1 | 0.439 | < 0.001 |
| AC007255.1 | AC007485.2 | 0.439 | < 0.001 |
| AC007255.1 | MAPKAPK5 | 0.439 | < 0.001 |
| AC007255.1 | AC025171.2 | 0.439 | < 0.001 |
| AC007255.1 | RPL17P10 | 0.439 | < 0.001 |
| AC007255.1 | MTATP6P26 | 0.438 | < 0.001 |
| AC007255.1 | MIR548C | 0.438 | < 0.001 |
| AC007255.1 | MTND4LP14 | 0.438 | < 0.001 |
| AC007255.1 | MT-CO3 | 0.438 | < 0.001 |
| AC007255.1 | D2HGDH | 0.438 | < 0.001 |
| AC007255.1 | AC127164.1 | 0.438 | < 0.001 |
| AC007255.1 | AC090877.1 | 0.438 | < 0.001 |
| AC007255.1 | DUSP27 | 0.438 | < 0.001 |
| AC007255.1 | RNU2-7P | 0.438 | < 0.001 |
| AC007255.1 | AC015914.1 | 0.438 | < 0.001 |
| AC007255.1 | AC004543.1 | 0.438 | < 0.001 |
| AC007255.1 | AC006042.3 | 0.438 | < 0.001 |
| AC007255.1 | CTBP2P4 | 0.438 | < 0.001 |
| AC007255.1 | KRT19P1 | 0.438 | < 0.001 |
| AC007255.1 | AC009962.1 | 0.438 | < 0.001 |
| AC007255.1 | AL391094.1 | 0.438 | < 0.001 |
| AC007255.1 | CAMSAP3 | 0.438 | < 0.001 |
| AC007255.1 | NMUR2 | 0.438 | < 0.001 |
| AC007255.1 | LINC02411 | 0.438 | < 0.001 |
| AC007255.1 | SGO1 | 0.438 | < 0.001 |
| AC007255.1 | HHEX | 0.438 | < 0.001 |
| AC007255.1 | PWWP2A | 0.438 | < 0.001 |
| AC007255.1 | SUGT1P2 | 0.438 | < 0.001 |
| AC007255.1 | AC131097.1 | 0.438 | < 0.001 |
| AC007255.1 | AC012499.1 | 0.437 | < 0.001 |
| AC007255.1 | AL121832.1 | 0.437 | < 0.001 |
| AC007255.1 | AP003086.2 | 0.437 | < 0.001 |
| AC007255.1 | LINC02041 | 0.437 | < 0.001 |
| AC007255.1 | ERVK3-1 | 0.437 | < 0.001 |
| AC007255.1 | RNU6-724P | 0.437 | < 0.001 |
| AC007255.1 | AC120498.4 | 0.437 | < 0.001 |
| AC007255.1 | GPR37L1 | 0.437 | < 0.001 |
| AC007255.1 | FRAT1 | 0.437 | < 0.001 |
| AC007255.1 | PPP1R11 | 0.437 | < 0.001 |
| AC007255.1 | MIR3127 | 0.437 | < 0.001 |
| AC007255.1 | TPSG1 | 0.437 | < 0.001 |
| AC007255.1 | XIAPP3 | 0.437 | < 0.001 |
| AC007255.1 | CSNK1D | 0.437 | < 0.001 |
| AC007255.1 | LMO7 | 0.437 | < 0.001 |
| AC007255.1 | RN7SL431P | 0.437 | < 0.001 |
| AC007255.1 | AC012676.5 | 0.437 | < 0.001 |
| AC007255.1 | SLC39A11 | 0.437 | < 0.001 |
| AC007255.1 | CMTM7 | 0.437 | < 0.001 |
| AC007255.1 | AC002070.1 | 0.437 | < 0.001 |
| AC007255.1 | ZFAND2B | 0.437 | < 0.001 |
| AC007255.1 | MT-ATP8 | 0.437 | < 0.001 |
| AC007255.1 | AC124319.1 | 0.437 | < 0.001 |
| AC007255.1 | DRAM2 | 0.437 | < 0.001 |
| AC007255.1 | SEPTIN7P9 | 0.437 | < 0.001 |
| AC007255.1 | AL031847.1 | 0.436 | < 0.001 |
| AC007255.1 | MINAR1 | 0.436 | < 0.001 |
| AC007255.1 | CARD8 | 0.436 | < 0.001 |
| AC007255.1 | TUBE1 | 0.436 | < 0.001 |
| AC007255.1 | LINC02615 | 0.436 | < 0.001 |
| AC007255.1 | AL445649.1 | 0.436 | < 0.001 |
| AC007255.1 | THOC2 | 0.436 | < 0.001 |
| AC007255.1 | PLPP5 | 0.436 | < 0.001 |
| AC007255.1 | PIWIL2 | 0.436 | < 0.001 |
| AC007255.1 | JSRP1 | 0.436 | < 0.001 |
| AC007255.1 | GSTA2 | 0.436 | < 0.001 |
| AC007255.1 | YWHABP2 | 0.436 | < 0.001 |
| AC007255.1 | LENG8 | 0.436 | < 0.001 |
| AC007255.1 | HSPA8P14 | 0.436 | < 0.001 |
| AC007255.1 | RN7SL28P | 0.436 | < 0.001 |
| AC007255.1 | AHSA2P | 0.436 | < 0.001 |
| AC007255.1 | EDRF1 | 0.436 | < 0.001 |
| AC007255.1 | AC005534.1 | 0.436 | < 0.001 |
| AC007255.1 | ASAH2 | 0.436 | < 0.001 |
| AC007255.1 | FAM149A | 0.436 | < 0.001 |
| AC007255.1 | CATIP | 0.436 | < 0.001 |
| AC007255.1 | CXXC5-AS1 | 0.436 | < 0.001 |
| AC007255.1 | PDRG1 | 0.435 | < 0.001 |
| AC007255.1 | AC104852.1 | 0.435 | < 0.001 |
| AC007255.1 | MPC1 | 0.435 | < 0.001 |
| AC007255.1 | MYZAP | 0.435 | < 0.001 |
| AC007255.1 | AP003733.3 | 0.435 | < 0.001 |
| AC007255.1 | CIDECP1 | 0.435 | < 0.001 |
| AC007255.1 | VTI1A | 0.435 | < 0.001 |
| AC007255.1 | AC036214.3 | 0.435 | < 0.001 |
| AC007255.1 | ACOT11 | 0.435 | < 0.001 |
| AC007255.1 | PRR5-ARHGAP8 | 0.435 | < 0.001 |
| AC007255.1 | PASK | 0.435 | < 0.001 |
| AC007255.1 | TMEM163 | 0.435 | < 0.001 |
| AC007255.1 | FOXD4L1 | 0.435 | < 0.001 |
| AC007255.1 | AC078962.4 | 0.435 | < 0.001 |
| AC007255.1 | AC022400.6 | 0.435 | < 0.001 |
| AC007255.1 | ACTL8 | 0.435 | < 0.001 |
| AC007255.1 | AC104836.1 | 0.435 | < 0.001 |
| AC007255.1 | AL035045.1 | 0.435 | < 0.001 |
| AC007255.1 | QRSL1 | 0.435 | < 0.001 |
| AC007255.1 | AC015849.5 | 0.435 | < 0.001 |
| AC007255.1 | GAPDHP55 | 0.435 | < 0.001 |
| AC007255.1 | AC024337.1 | 0.435 | < 0.001 |
| AC007255.1 | GPR83 | 0.434 | < 0.001 |
| AC007255.1 | AC135048.4 | 0.434 | < 0.001 |
| AC007255.1 | RPARP-AS1 | 0.434 | < 0.001 |
| AC007255.1 | LDAH | 0.434 | < 0.001 |
| AC007255.1 | AC092375.2 | 0.434 | < 0.001 |
| AC007255.1 | ARMC7 | 0.434 | < 0.001 |
| AC007255.1 | IRAK2 | 0.434 | < 0.001 |
| AC007255.1 | CHUK | 0.434 | < 0.001 |
| AC007255.1 | AC005355.2 | 0.434 | < 0.001 |
| AC007255.1 | AL161669.1 | 0.434 | < 0.001 |
| AC007255.1 | AC091059.2 | 0.434 | < 0.001 |
| AC007255.1 | MIR3944 | 0.434 | < 0.001 |
| AC007255.1 | AC092723.1 | 0.434 | < 0.001 |
| AC007255.1 | OSBP | 0.434 | < 0.001 |
| AC007255.1 | EFCAB13 | 0.434 | < 0.001 |
| AC007255.1 | LINC01909 | 0.434 | < 0.001 |
| AC007255.1 | DDX10P1 | 0.434 | < 0.001 |
| AC007255.1 | PDZK1IP1 | 0.434 | < 0.001 |
| AC007255.1 | AC092802.4 | 0.434 | < 0.001 |
| AC007255.1 | AP002812.5 | 0.433 | < 0.001 |
| AC007255.1 | RNU6-1095P | 0.433 | < 0.001 |
| AC007255.1 | AP003498.2 | 0.433 | < 0.001 |
| AC007255.1 | AP002340.1 | 0.433 | < 0.001 |
| AC007255.1 | FAM78B-AS1 | 0.433 | < 0.001 |
| AC007255.1 | USPL1 | 0.433 | < 0.001 |
| AC007255.1 | HLA-Z | 0.433 | < 0.001 |
| AC007255.1 | JAK1 | 0.433 | < 0.001 |
| AC007255.1 | KRT8P18 | 0.433 | < 0.001 |
| AC007255.1 | TIGD3 | 0.433 | < 0.001 |
| AC007255.1 | GNS | 0.433 | < 0.001 |
| AC007255.1 | PROK1 | 0.433 | < 0.001 |
| AC007255.1 | AC073107.2 | 0.433 | < 0.001 |
| AC007255.1 | SKAP1-AS1 | 0.433 | < 0.001 |
| AC007255.1 | C3orf86 | 0.433 | < 0.001 |
| AC007255.1 | SORBS2 | 0.432 | < 0.001 |
| AC007255.1 | VIPR1 | 0.432 | < 0.001 |
| AC007255.1 | SPHK2 | 0.432 | < 0.001 |
| AC007255.1 | TSTA3 | 0.432 | < 0.001 |
| AC007255.1 | NOS1AP | 0.432 | < 0.001 |
| AC007255.1 | GPR20 | 0.432 | < 0.001 |
| AC007255.1 | FAM50A | 0.432 | < 0.001 |
| AC007255.1 | CLCN2 | 0.432 | < 0.001 |
| AC007255.1 | AC068888.2 | 0.432 | < 0.001 |
| AC007255.1 | PLA2G2E | 0.432 | < 0.001 |
| AC007255.1 | AL356740.1 | 0.432 | < 0.001 |
| AC007255.1 | CCT6P3 | 0.432 | < 0.001 |
| AC007255.1 | RDM1P5 | 0.432 | < 0.001 |
| AC007255.1 | ZNF814 | 0.432 | < 0.001 |
| AC007255.1 | TNFRSF21 | 0.432 | < 0.001 |
| AC007255.1 | PHACTR2 | 0.432 | < 0.001 |
| AC007255.1 | C10orf95 | 0.432 | < 0.001 |
| AC007255.1 | C2CD5 | 0.432 | < 0.001 |
| AC007255.1 | CRIP1 | 0.431 | < 0.001 |
| AC007255.1 | AC022395.1 | 0.431 | < 0.001 |
| AC007255.1 | CEACAM7 | 0.431 | < 0.001 |
| AC007255.1 | EPHB2 | 0.431 | < 0.001 |
| AC007255.1 | AP000866.6 | 0.431 | < 0.001 |
| AC007255.1 | TEX9 | 0.431 | < 0.001 |
| AC007255.1 | ZNF473 | 0.431 | < 0.001 |
| AC007255.1 | ZNF37A | 0.431 | < 0.001 |
| AC007255.1 | TMED2-DT | 0.431 | < 0.001 |
| AC007255.1 | ARHGAP8 | 0.431 | < 0.001 |
| AC007255.1 | RERE-AS1 | 0.431 | < 0.001 |
| AC007255.1 | AP001372.2 | 0.431 | < 0.001 |
| AC007255.1 | VN1R48P | 0.431 | < 0.001 |
| AC007255.1 | PPCS | 0.431 | < 0.001 |
| AC007255.1 | AC093227.1 | 0.431 | < 0.001 |
| AC007255.1 | AL354707.1 | 0.431 | < 0.001 |
| AC007255.1 | TMED6 | 0.430 | < 0.001 |
| AC007255.1 | EZR-AS1 | 0.430 | < 0.001 |
| AC007255.1 | LIPT1 | 0.430 | < 0.001 |
| AC007255.1 | RNFT1 | 0.430 | < 0.001 |
| AC007255.1 | PREPL | 0.430 | < 0.001 |
| AC007255.1 | TMEM253 | 0.430 | < 0.001 |
| AC007255.1 | AC005332.5 | 0.430 | < 0.001 |
| AC007255.1 | AMIGO1 | 0.430 | < 0.001 |
| AC007255.1 | NUFIP2 | 0.430 | < 0.001 |
| AC007255.1 | AP002812.1 | 0.430 | < 0.001 |
| AC007255.1 | AC111170.4 | 0.430 | < 0.001 |
| AC007255.1 | CLN3 | 0.430 | < 0.001 |
| AC007255.1 | MED12 | 0.430 | < 0.001 |
| AC007255.1 | ZNF565 | 0.430 | < 0.001 |
| AC007255.1 | KBTBD3 | 0.430 | < 0.001 |
| AC007255.1 | KIF26B-AS1 | 0.430 | < 0.001 |
| AC007255.1 | AC010306.1 | 0.430 | < 0.001 |
| AC007255.1 | AC091181.1 | 0.429 | < 0.001 |
| AC007255.1 | DTNBP1 | 0.429 | < 0.001 |
| AC007255.1 | NOX1 | 0.429 | < 0.001 |
| AC007255.1 | RP2 | 0.429 | < 0.001 |
| AC007255.1 | SLC50A1 | 0.429 | < 0.001 |
| AC007255.1 | AL161421.1 | 0.429 | < 0.001 |
| AC007255.1 | SPRED2 | 0.429 | < 0.001 |
| AC007255.1 | SAYSD1 | 0.429 | < 0.001 |
| AC007255.1 | CYP2T1P | 0.429 | < 0.001 |
| AC007255.1 | AL391840.1 | 0.429 | < 0.001 |
| AC007255.1 | KRT18P13 | 0.429 | < 0.001 |
| AC007255.1 | BEND3 | 0.429 | < 0.001 |
| AC007255.1 | AC020594.1 | 0.429 | < 0.001 |
| AC007255.1 | RNA5SP53 | 0.429 | < 0.001 |
| AC007255.1 | AL356740.3 | 0.428 | < 0.001 |
| AC007255.1 | TTR | 0.428 | < 0.001 |
| AC007255.1 | C19orf67 | 0.428 | < 0.001 |
| AC007255.1 | AC137630.2 | 0.428 | < 0.001 |
| AC007255.1 | MPC2 | 0.428 | < 0.001 |
| AC007255.1 | AAMP | 0.428 | < 0.001 |
| AC007255.1 | MTCO1P11 | 0.428 | < 0.001 |
| AC007255.1 | PEX11G | 0.428 | < 0.001 |
| AC007255.1 | AL356481.2 | 0.428 | < 0.001 |
| AC007255.1 | SYDE2 | 0.428 | < 0.001 |
| AC007255.1 | HMGB3P2 | 0.428 | < 0.001 |
| AC007255.1 | AGAP6 | 0.428 | < 0.001 |
| AC007255.1 | CASP6 | 0.428 | < 0.001 |
| AC007255.1 | LINC02804 | 0.428 | < 0.001 |
| AC007255.1 | TEX11 | 0.428 | < 0.001 |
| AC007255.1 | SNORD124 | 0.428 | < 0.001 |
| AC007255.1 | C6orf89 | 0.428 | < 0.001 |
| AC007255.1 | CCDC30 | 0.428 | < 0.001 |
| AC007255.1 | SLC7A9 | 0.428 | < 0.001 |
| AC007255.1 | TPM3P6 | 0.428 | < 0.001 |
| AC007255.1 | LINC02649 | 0.428 | < 0.001 |
| AC007255.1 | CYP4F2 | 0.428 | < 0.001 |
| AC007255.1 | ERVW-1 | 0.427 | < 0.001 |
| AC007255.1 | THRB-IT1 | 0.427 | < 0.001 |
| AC007255.1 | CWC25 | 0.427 | < 0.001 |
| AC007255.1 | NFKBIE | 0.427 | < 0.001 |
| AC007255.1 | AP001893.1 | 0.427 | < 0.001 |
| AC007255.1 | FAIM2 | 0.427 | < 0.001 |
| AC007255.1 | CCHCR1 | 0.427 | < 0.001 |
| AC007255.1 | AC008147.4 | 0.427 | < 0.001 |
| AC007255.1 | AL365205.1 | 0.427 | < 0.001 |
| AC007255.1 | SNORA15B-1 | 0.427 | < 0.001 |
| AC007255.1 | IL13RA1 | 0.427 | < 0.001 |
| AC007255.1 | AL590822.2 | 0.427 | < 0.001 |
| AC007255.1 | AC226118.1 | 0.427 | < 0.001 |
| AC007255.1 | ATXN7 | 0.427 | < 0.001 |
| AC007255.1 | SNORD19 | 0.427 | < 0.001 |
| AC007255.1 | AC148477.4 | 0.427 | < 0.001 |
| AC007255.1 | AL158068.2 | 0.427 | < 0.001 |
| AC007255.1 | MCIDAS | 0.427 | < 0.001 |
| AC007255.1 | MTTP | 0.427 | < 0.001 |
| AC007255.1 | AC123023.1 | 0.427 | < 0.001 |
| AC007255.1 | GCOM1 | 0.427 | < 0.001 |
| AC007255.1 | AC026464.5 | 0.427 | < 0.001 |
| AC007255.1 | IFNAR2 | 0.427 | < 0.001 |
| AC007255.1 | AC089985.1 | 0.427 | < 0.001 |
| AC007255.1 | RPL21P7 | 0.427 | < 0.001 |
| AC007255.1 | AC007919.2 | 0.427 | < 0.001 |
| AC007255.1 | GBA3 | 0.427 | < 0.001 |
| AC007255.1 | C2 | 0.427 | < 0.001 |
| AC007255.1 | PRSS16 | 0.427 | < 0.001 |
| AC007255.1 | WFDC13 | 0.427 | < 0.001 |
| AC007255.1 | AC027117.1 | 0.427 | < 0.001 |
| AC007255.1 | F10 | 0.426 | < 0.001 |
| AC007255.1 | OR2I1P | 0.426 | < 0.001 |
| AC007255.1 | CFL1P5 | 0.426 | < 0.001 |
| AC007255.1 | MGLL | 0.426 | < 0.001 |
| AC007255.1 | LSMEM2 | 0.426 | < 0.001 |
| AC007255.1 | LINC01273 | 0.426 | < 0.001 |
| AC007255.1 | AC009495.1 | 0.426 | < 0.001 |
| AC007255.1 | LINC02418 | 0.426 | < 0.001 |
| AC007255.1 | BANK1 | 0.426 | < 0.001 |
| AC007255.1 | TIMP4 | 0.426 | < 0.001 |
| AC007255.1 | MAGI1-IT1 | 0.426 | < 0.001 |
| AC007255.1 | DOP1A | 0.426 | < 0.001 |
| AC007255.1 | ZNF701 | 0.426 | < 0.001 |
| AC007255.1 | AP001767.4 | 0.426 | < 0.001 |
| AC007255.1 | AL355987.4 | 0.426 | < 0.001 |
| AC007255.1 | AL136531.2 | 0.426 | < 0.001 |
| AC007255.1 | MICAL2 | 0.426 | < 0.001 |
| AC007255.1 | ZNF546 | 0.426 | < 0.001 |
| AC007255.1 | SAP30L-AS1 | 0.426 | < 0.001 |
| AC007255.1 | AP001767.3 | 0.426 | < 0.001 |
| AC007255.1 | TRIQK | 0.425 | < 0.001 |
| AC007255.1 | AC097381.3 | 0.425 | < 0.001 |
| AC007255.1 | RALGAPB | 0.425 | < 0.001 |
| AC007255.1 | SCML4 | 0.425 | < 0.001 |
| AC007255.1 | AL137847.1 | 0.425 | < 0.001 |
| AC007255.1 | MUC20P1 | 0.425 | < 0.001 |
| AC007255.1 | AC091117.3 | 0.425 | < 0.001 |
| AC007255.1 | EAF1-AS1 | 0.425 | < 0.001 |
| AC007255.1 | AMPD2 | 0.425 | < 0.001 |
| AC007255.1 | Z95152.1 | 0.425 | < 0.001 |
| AC007255.1 | AC007327.1 | 0.425 | < 0.001 |
| AC007255.1 | IL7 | 0.425 | < 0.001 |
| AC007255.1 | AC087465.1 | 0.425 | < 0.001 |
| AC007255.1 | AC013489.3 | 0.425 | < 0.001 |
| AC007255.1 | AL035448.1 | 0.425 | < 0.001 |
| AC007255.1 | LINC02404 | 0.425 | < 0.001 |
| AC007255.1 | AL356481.3 | 0.425 | < 0.001 |
| AC007255.1 | SRL | 0.425 | < 0.001 |
| AC007255.1 | NAGLU | 0.425 | < 0.001 |
| AC007255.1 | OVGP1 | 0.425 | < 0.001 |
| AC007255.1 | TFR2 | 0.425 | < 0.001 |
| AC007255.1 | ORM2 | 0.425 | < 0.001 |
| AC007255.1 | POU2F1 | 0.425 | < 0.001 |
| AC007255.1 | CD2BP2-DT | 0.425 | < 0.001 |
| AC007255.1 | AC025171.4 | 0.425 | < 0.001 |
| AC007255.1 | MTG1 | 0.425 | < 0.001 |
| AC007255.1 | MTPAP | 0.425 | < 0.001 |
| AC007255.1 | EVI5 | 0.425 | < 0.001 |
| AC007255.1 | AC244090.1 | 0.424 | < 0.001 |
| AC007255.1 | RNU6-1128P | 0.424 | < 0.001 |
| AC007255.1 | FGF20 | 0.424 | < 0.001 |
| AC007255.1 | SPNS2 | 0.424 | < 0.001 |
| AC007255.1 | AC013439.1 | 0.424 | < 0.001 |
| AC007255.1 | AL356299.3 | 0.424 | < 0.001 |
| AC007255.1 | ZNF585B | 0.424 | < 0.001 |
| AC007255.1 | AC239800.2 | 0.424 | < 0.001 |
| AC007255.1 | SCARNA5 | 0.424 | < 0.001 |
| AC007255.1 | FOXP1-IT1 | 0.424 | < 0.001 |
| AC007255.1 | AL590666.2 | 0.424 | < 0.001 |
| AC007255.1 | SNORD13P3 | 0.424 | < 0.001 |
| AC007255.1 | MIR4284 | 0.424 | < 0.001 |
| AC007255.1 | PCNPP1 | 0.424 | < 0.001 |
| AC007255.1 | AC097721.1 | 0.424 | < 0.001 |
| AC007255.1 | MMP24 | 0.424 | < 0.001 |
| AC007255.1 | COLCA1 | 0.423 | < 0.001 |
| AC007255.1 | AL158824.2 | 0.423 | < 0.001 |
| AC007255.1 | NME6 | 0.423 | < 0.001 |
| AC007255.1 | USP45 | 0.423 | < 0.001 |
| AC007255.1 | RAB40B | 0.423 | < 0.001 |
| AC007255.1 | PKD1L1 | 0.423 | < 0.001 |
| AC007255.1 | AC138932.6 | 0.423 | < 0.001 |
| AC007255.1 | SFXN4 | 0.423 | < 0.001 |
| AC007255.1 | BTBD9 | 0.423 | < 0.001 |
| AC007255.1 | LINC01355 | 0.423 | < 0.001 |
| AC007255.1 | AC139887.2 | 0.423 | < 0.001 |
| AC007255.1 | LIF-AS1 | 0.423 | < 0.001 |
| AC007255.1 | SATB2 | 0.423 | < 0.001 |
| AC007255.1 | HOXB-AS4 | 0.423 | < 0.001 |
| AC007255.1 | DENND4C | 0.423 | < 0.001 |
| AC007255.1 | AL451042.1 | 0.423 | < 0.001 |
| AC007255.1 | PPP1R26 | 0.423 | < 0.001 |
| AC007255.1 | AC010976.1 | 0.423 | < 0.001 |
| AC007255.1 | SEPTIN7P2 | 0.423 | < 0.001 |
| AC007255.1 | UACA | 0.423 | < 0.001 |
| AC007255.1 | AC017074.1 | 0.423 | < 0.001 |
| AC007255.1 | AC024267.6 | 0.423 | < 0.001 |
| AC007255.1 | LINC01357 | 0.423 | < 0.001 |
| AC007255.1 | MTND2P40 | 0.423 | < 0.001 |
| AC007255.1 | AL158801.1 | 0.423 | < 0.001 |
| AC007255.1 | B3GNTL1 | 0.423 | < 0.001 |
| AC007255.1 | RN7SL684P | 0.422 | < 0.001 |
| AC007255.1 | NRADDP | 0.422 | < 0.001 |
| AC007255.1 | AC069281.2 | 0.422 | < 0.001 |
| AC007255.1 | GDPD4 | 0.422 | < 0.001 |
| AC007255.1 | TXK | 0.422 | < 0.001 |
| AC007255.1 | AKAP13 | 0.422 | < 0.001 |
| AC007255.1 | RBPMS-AS1 | 0.422 | < 0.001 |
| AC007255.1 | AC092569.1 | 0.422 | < 0.001 |
| AC007255.1 | SLC35E2A | 0.422 | < 0.001 |
| AC007255.1 | DCAF1 | 0.422 | < 0.001 |
| AC007255.1 | TECPR1 | 0.422 | < 0.001 |
| AC007255.1 | CUTALP | 0.422 | < 0.001 |
| AC007255.1 | GTF3A | 0.422 | < 0.001 |
| AC007255.1 | PPT2-EGFL8 | 0.422 | < 0.001 |
| AC007255.1 | SAMD10 | 0.422 | < 0.001 |
| AC007255.1 | NCOR1 | 0.422 | < 0.001 |
| AC007255.1 | RNPEPL1 | 0.422 | < 0.001 |
| AC007255.1 | SLC18A2 | 0.421 | < 0.001 |
| AC007255.1 | UBE2D4 | 0.421 | < 0.001 |
| AC007255.1 | RNA5SP139 | 0.421 | < 0.001 |
| AC007255.1 | CLIC6 | 0.421 | < 0.001 |
| AC007255.1 | FAM122C | 0.421 | < 0.001 |
| AC007255.1 | RN7SL182P | 0.421 | < 0.001 |
| AC007255.1 | TMEM168 | 0.421 | < 0.001 |
| AC007255.1 | ECEL1P2 | 0.421 | < 0.001 |
| AC007255.1 | CD302 | 0.421 | < 0.001 |
| AC007255.1 | KCNJ13 | 0.421 | < 0.001 |
| AC007255.1 | PDCD6IP | 0.421 | < 0.001 |
| AC007255.1 | AC010997.3 | 0.421 | < 0.001 |
| AC007255.1 | STPG3-AS1 | 0.421 | < 0.001 |
| AC007255.1 | PROSER1 | 0.421 | < 0.001 |
| AC007255.1 | NNT-AS1 | 0.421 | < 0.001 |
| AC007255.1 | AC009831.3 | 0.421 | < 0.001 |
| AC007255.1 | ONECUT3 | 0.421 | < 0.001 |
| AC007255.1 | CXorf65 | 0.421 | < 0.001 |
| AC007255.1 | AL359263.1 | 0.421 | < 0.001 |
| AC007255.1 | DISP2 | 0.421 | < 0.001 |
| AC007255.1 | IL10RB | 0.421 | < 0.001 |
| AC007255.1 | MIR556 | 0.421 | < 0.001 |
| AC007255.1 | LINC01079 | 0.421 | < 0.001 |
| AC007255.1 | AL365181.3 | 0.421 | < 0.001 |
| AC007255.1 | AP000925.1 | 0.421 | < 0.001 |
| AC007255.1 | RN7SKP78 | 0.421 | < 0.001 |
| AC007255.1 | SEC24A | 0.421 | < 0.001 |
| AC007255.1 | AC009163.6 | 0.421 | < 0.001 |
| AC007255.1 | ACTR5 | 0.421 | < 0.001 |
| AC007255.1 | AP001085.1 | 0.421 | < 0.001 |
| AC007255.1 | KRT8P9 | 0.421 | < 0.001 |
| AC007255.1 | ST6GALNAC3 | 0.421 | < 0.001 |
| AC007255.1 | AC025188.1 | 0.421 | < 0.001 |
| AC007255.1 | MIR4767 | 0.421 | < 0.001 |
| AC007255.1 | ID2 | 0.421 | < 0.001 |
| AC007255.1 | AP003059.2 | 0.421 | < 0.001 |
| AC007255.1 | PDIA3 | 0.420 | < 0.001 |
| AC007255.1 | LINC01285 | 0.420 | < 0.001 |
| AC007255.1 | RNA5SP111 | 0.420 | < 0.001 |
| AC007255.1 | PRRG1 | 0.420 | < 0.001 |
| AC007255.1 | PRR29 | 0.420 | < 0.001 |
| AC007255.1 | CREBZF | 0.420 | < 0.001 |
| AC007255.1 | RNU6-1061P | 0.420 | < 0.001 |
| AC007255.1 | AC106782.1 | 0.420 | < 0.001 |
| AC007255.1 | HSPA8P3 | 0.420 | < 0.001 |
| AC007255.1 | SCGB2A1 | 0.420 | < 0.001 |
| AC007255.1 | TBX3 | 0.420 | < 0.001 |
| AC007255.1 | AC090627.1 | 0.420 | < 0.001 |
| AC007255.1 | ZNF487 | 0.420 | < 0.001 |
| AC007255.1 | LCA5L | 0.420 | < 0.001 |
| AC007255.1 | ERGIC3 | 0.420 | < 0.001 |
| AC007255.1 | AL136982.3 | 0.420 | < 0.001 |
| AC007255.1 | AC007405.2 | 0.420 | < 0.001 |
| AC007255.1 | AC005785.2 | 0.420 | < 0.001 |
| AC007255.1 | NXPE1 | 0.420 | < 0.001 |
| AC007255.1 | MPHOSPH8 | 0.420 | < 0.001 |
| AC007255.1 | SP140L | 0.420 | < 0.001 |
| AC007255.1 | VPS37B | 0.420 | < 0.001 |
| AC007255.1 | Z99127.3 | 0.420 | < 0.001 |
| AC007255.1 | AC002128.1 | 0.419 | < 0.001 |
| AC007255.1 | CCDC84 | 0.419 | < 0.001 |
| AC007255.1 | ZNF630 | 0.419 | < 0.001 |
| AC007255.1 | UBP1 | 0.419 | < 0.001 |
| AC007255.1 | APOA5 | 0.419 | < 0.001 |
| AC007255.1 | GRB14 | 0.419 | < 0.001 |
| AC007255.1 | AL390208.1 | 0.419 | < 0.001 |
| AC007255.1 | AP000346.3 | 0.419 | < 0.001 |
| AC007255.1 | AC106827.1 | 0.419 | < 0.001 |
| AC007255.1 | MTND4LP22 | 0.419 | < 0.001 |
| AC007255.1 | AC005091.1 | 0.419 | < 0.001 |
| AC007255.1 | ARAF | 0.419 | < 0.001 |
| AC007255.1 | FAM199X | 0.419 | < 0.001 |
| AC007255.1 | RN7SL735P | 0.419 | < 0.001 |
| AC007255.1 | MIR6812 | 0.419 | < 0.001 |
| AC007255.1 | AC107072.1 | 0.419 | < 0.001 |
| AC007255.1 | AC092171.4 | 0.419 | < 0.001 |
| AC007255.1 | PAN3 | 0.419 | < 0.001 |
| AC007255.1 | PRSS48 | 0.418 | < 0.001 |
| AC007255.1 | AC010967.1 | 0.418 | < 0.001 |
| AC007255.1 | LDHD | 0.418 | < 0.001 |
| AC007255.1 | AL353572.1 | 0.418 | < 0.001 |
| AC007255.1 | ARL6IP5 | 0.418 | < 0.001 |
| AC007255.1 | EIF4E2P1 | 0.418 | < 0.001 |
| AC007255.1 | ESPNP | 0.418 | < 0.001 |
| AC007255.1 | PLEKHA8 | 0.418 | < 0.001 |
| AC007255.1 | AL360219.2 | 0.418 | < 0.001 |
| AC007255.1 | CEP295NL | 0.418 | < 0.001 |
| AC007255.1 | ZNRD1ASP | 0.418 | < 0.001 |
| AC007255.1 | RNU6-1327P | 0.418 | < 0.001 |
| AC007255.1 | HSD3B7 | 0.418 | < 0.001 |
| AC007255.1 | PREP | 0.418 | < 0.001 |
| AC007255.1 | ZFHX2 | 0.417 | < 0.001 |
| AC007255.1 | PPCDC | 0.417 | < 0.001 |
| AC007255.1 | OR6L2P | 0.417 | < 0.001 |
| AC007255.1 | SDCBP | 0.417 | < 0.001 |
| AC007255.1 | HRC | 0.417 | < 0.001 |
| AC007255.1 | RGL4 | 0.417 | < 0.001 |
| AC007255.1 | MIR3197 | 0.417 | < 0.001 |
| AC007255.1 | VWA3A | 0.417 | < 0.001 |
| AC007255.1 | EDRF1-AS1 | 0.417 | < 0.001 |
| AC007255.1 | Z99572.1 | 0.417 | < 0.001 |
| AC007255.1 | AC116407.4 | 0.417 | < 0.001 |
| AC007255.1 | PLAC9 | 0.417 | < 0.001 |
| AC007255.1 | VN1R83P | 0.417 | < 0.001 |
| AC007255.1 | AC008735.1 | 0.417 | < 0.001 |
| AC007255.1 | HABP4 | 0.417 | < 0.001 |
| AC007255.1 | HDAC6 | 0.417 | < 0.001 |
| AC007255.1 | RBM5 | 0.417 | < 0.001 |
| AC007255.1 | FZD4-DT | 0.417 | < 0.001 |
| AC007255.1 | PGGHG | 0.417 | < 0.001 |
| AC007255.1 | CCDC112 | 0.417 | < 0.001 |
| AC007255.1 | AP000345.2 | 0.417 | < 0.001 |
| AC007255.1 | PSMD10P2 | 0.417 | < 0.001 |
| AC007255.1 | BCAN | 0.417 | < 0.001 |
| AC007255.1 | MAD2L1BP | 0.417 | < 0.001 |
| AC007255.1 | KNG1 | 0.417 | < 0.001 |
| AC007255.1 | AL353622.2 | 0.416 | < 0.001 |
| AC007255.1 | MTNR1A | 0.416 | < 0.001 |
| AC007255.1 | ATG4B | 0.416 | < 0.001 |
| AC007255.1 | AC137770.1 | 0.416 | < 0.001 |
| AC007255.1 | AC116366.2 | 0.416 | < 0.001 |
| AC007255.1 | AC114763.2 | 0.416 | < 0.001 |
| AC007255.1 | LINC01612 | 0.416 | < 0.001 |
| AC007255.1 | ACTR8 | 0.416 | < 0.001 |
| AC007255.1 | WWP2 | 0.416 | < 0.001 |
| AC007255.1 | FNIP2 | 0.416 | < 0.001 |
| AC007255.1 | ZNF836 | 0.416 | < 0.001 |
| AC007255.1 | WDR48 | 0.416 | < 0.001 |
| AC007255.1 | AC092801.1 | 0.416 | < 0.001 |
| AC007255.1 | GRIPAP1 | 0.416 | < 0.001 |
| AC007255.1 | ZNF101 | 0.416 | < 0.001 |
| AC007255.1 | C2orf92 | 0.416 | < 0.001 |
| AC007255.1 | PLEKHG6 | 0.416 | < 0.001 |
| AC007255.1 | MID1IP1 | 0.416 | < 0.001 |
| AC007255.1 | AC087741.1 | 0.416 | < 0.001 |
| AC007255.1 | RN7SKP173 | 0.416 | < 0.001 |
| AC007255.1 | PTGES2 | 0.416 | < 0.001 |
| AC007255.1 | PHYKPL | 0.415 | < 0.001 |
| AC007255.1 | TRMT2B-AS1 | 0.415 | < 0.001 |
| AC007255.1 | MSX2 | 0.415 | < 0.001 |
| AC007255.1 | ENTPD4 | 0.415 | < 0.001 |
| AC007255.1 | LINC01942 | 0.415 | < 0.001 |
| AC007255.1 | ICAM2 | 0.415 | < 0.001 |
| AC007255.1 | HERPUD1 | 0.415 | < 0.001 |
| AC007255.1 | RPRD1B | 0.415 | < 0.001 |
| AC007255.1 | AC007684.1 | 0.415 | < 0.001 |
| AC007255.1 | AL359878.1 | 0.415 | < 0.001 |
| AC007255.1 | SRSF6 | 0.415 | < 0.001 |
| AC007255.1 | LSMEM1 | 0.415 | < 0.001 |
| AC007255.1 | PLVAP | 0.415 | < 0.001 |
| AC007255.1 | AC091057.1 | 0.415 | < 0.001 |
| AC007255.1 | AMD1P3 | 0.415 | < 0.001 |
| AC007255.1 | PARGP1 | 0.415 | < 0.001 |
| AC007255.1 | SLC6A18 | 0.415 | < 0.001 |
| AC007255.1 | GLCCI1-DT | 0.415 | < 0.001 |
| AC007255.1 | SNORA11F | 0.415 | < 0.001 |
| AC007255.1 | PDCL3P4 | 0.415 | < 0.001 |
| AC007255.1 | P2RX3 | 0.415 | < 0.001 |
| AC007255.1 | ASAH1 | 0.415 | < 0.001 |
| AC007255.1 | AL109614.1 | 0.415 | < 0.001 |
| AC007255.1 | AL445231.1 | 0.415 | < 0.001 |
| AC007255.1 | DAP3P1 | 0.414 | < 0.001 |
| AC007255.1 | VNN2 | 0.414 | < 0.001 |
| AC007255.1 | ZFYVE28 | 0.414 | < 0.001 |
| AC007255.1 | AC009163.4 | 0.414 | < 0.001 |
| AC007255.1 | MT-CYB | 0.414 | < 0.001 |
| AC007255.1 | RNF207 | 0.414 | < 0.001 |
| AC007255.1 | AL136418.1 | 0.414 | < 0.001 |
| AC007255.1 | NPHS1 | 0.414 | < 0.001 |
| AC007255.1 | TMEM98 | 0.414 | < 0.001 |
| AC007255.1 | EDNRB | 0.414 | < 0.001 |
| AC007255.1 | AL137077.1 | 0.414 | < 0.001 |
| AC007255.1 | MIR4525 | 0.414 | < 0.001 |
| AC007255.1 | RDH13 | 0.414 | < 0.001 |
| AC007255.1 | MT-ND2 | 0.414 | < 0.001 |
| AC007255.1 | MIR3161 | 0.414 | < 0.001 |
| AC007255.1 | AC104958.1 | 0.414 | < 0.001 |
| AC007255.1 | AC006059.1 | 0.414 | < 0.001 |
| AC007255.1 | AC021744.1 | 0.414 | < 0.001 |
| AC007255.1 | AC090948.3 | 0.414 | < 0.001 |
| AC007255.1 | HMGA1P4 | 0.414 | < 0.001 |
| AC007255.1 | DNPEP | 0.414 | < 0.001 |
| AC007255.1 | LINC01767 | 0.414 | < 0.001 |
| AC007255.1 | AL158801.3 | 0.414 | < 0.001 |
| AC007255.1 | WFS1 | 0.414 | < 0.001 |
| AC007255.1 | AL031716.1 | 0.414 | < 0.001 |
| AC007255.1 | ZSCAN9 | 0.414 | < 0.001 |
| AC007255.1 | CLCA1 | 0.414 | < 0.001 |
| AC007255.1 | AL356752.1 | 0.414 | < 0.001 |
| AC007255.1 | ABHD15 | 0.414 | < 0.001 |
| AC007255.1 | AL365356.1 | 0.413 | < 0.001 |
| AC007255.1 | ASAH2B | 0.413 | < 0.001 |
| AC007255.1 | AC016727.1 | 0.413 | < 0.001 |
| AC007255.1 | RNU2-33P | 0.413 | < 0.001 |
| AC007255.1 | SNHG20 | 0.413 | < 0.001 |
| AC007255.1 | MIR29B2CHG | 0.413 | < 0.001 |
| AC007255.1 | KLF2 | 0.413 | < 0.001 |
| AC007255.1 | SMIM35 | 0.413 | < 0.001 |
| AC007255.1 | TOR1B | 0.413 | < 0.001 |
| AC007255.1 | MT-CO2 | 0.413 | < 0.001 |
| AC007255.1 | AC133041.1 | 0.413 | < 0.001 |
| AC007255.1 | ZNF702P | 0.413 | < 0.001 |
| AC007255.1 | SCAND2P | 0.413 | < 0.001 |
| AC007255.1 | C9orf24 | 0.413 | < 0.001 |
| AC007255.1 | AL121723.1 | 0.413 | < 0.001 |
| AC007255.1 | AC016999.1 | 0.413 | < 0.001 |
| AC007255.1 | STAU1 | 0.413 | < 0.001 |
| AC007255.1 | ZNF501 | 0.413 | < 0.001 |
| AC007255.1 | ACSS1 | 0.413 | < 0.001 |
| AC007255.1 | SUZ12 | 0.413 | < 0.001 |
| AC007255.1 | AP001628.1 | 0.413 | < 0.001 |
| AC007255.1 | MIGA2 | 0.412 | < 0.001 |
| AC007255.1 | AC067940.1 | 0.412 | < 0.001 |
| AC007255.1 | AL451047.1 | 0.412 | < 0.001 |
| AC007255.1 | RN7SL220P | 0.412 | < 0.001 |
| AC007255.1 | RPS2P36 | 0.412 | < 0.001 |
| AC007255.1 | STT3A | 0.412 | < 0.001 |
| AC007255.1 | MAK | 0.412 | < 0.001 |
| AC007255.1 | ZNF491 | 0.412 | < 0.001 |
| AC007255.1 | FAM222B | 0.412 | < 0.001 |
| AC007255.1 | TMEM126B | 0.412 | < 0.001 |
| AC007255.1 | LINC00271 | 0.412 | < 0.001 |
| AC007255.1 | AC145207.9 | 0.412 | < 0.001 |
| AC007255.1 | VWA5A | 0.412 | < 0.001 |
| AC007255.1 | BNIP3P4 | 0.412 | < 0.001 |
| AC007255.1 | AC022558.1 | 0.412 | < 0.001 |
| AC007255.1 | PIGG | 0.412 | < 0.001 |
| AC007255.1 | AC244093.5 | 0.412 | < 0.001 |
| AC007255.1 | TPP2 | 0.412 | < 0.001 |
| AC007255.1 | LINC01474 | 0.412 | < 0.001 |
| AC007255.1 | AC138393.3 | 0.412 | < 0.001 |
| AC007255.1 | DENND3 | 0.412 | < 0.001 |
| AC007255.1 | MTND4P14 | 0.412 | < 0.001 |
| AC007255.1 | ZNF214 | 0.412 | < 0.001 |
| AC007255.1 | TCF7 | 0.412 | < 0.001 |
| AC007255.1 | DNASE1 | 0.412 | < 0.001 |
| AC007255.1 | CHMP1B2P | 0.411 | < 0.001 |
| AC007255.1 | AP003170.5 | 0.411 | < 0.001 |
| AC007255.1 | SLC37A3 | 0.411 | < 0.001 |
| AC007255.1 | POLG2 | 0.411 | < 0.001 |
| AC007255.1 | NBEAL1 | 0.411 | < 0.001 |
| AC007255.1 | C17orf113 | 0.411 | < 0.001 |
| AC007255.1 | RN7SL549P | 0.411 | < 0.001 |
| AC007255.1 | USP8 | 0.411 | < 0.001 |
| AC007255.1 | AC138932.4 | 0.411 | < 0.001 |
| AC007255.1 | MIR616 | 0.411 | < 0.001 |
| AC007255.1 | QPRT | 0.411 | < 0.001 |
| AC007255.1 | AC007982.1 | 0.411 | < 0.001 |
| AC007255.1 | WDR73 | 0.411 | < 0.001 |
| AC007255.1 | AL132657.2 | 0.411 | < 0.001 |
| AC007255.1 | AL160314.3 | 0.411 | < 0.001 |
| AC007255.1 | STRC | 0.411 | < 0.001 |
| AC007255.1 | PLCD3 | 0.411 | < 0.001 |
| AC007255.1 | AC025265.1 | 0.411 | < 0.001 |
| AC007255.1 | AL354809.1 | 0.411 | < 0.001 |
| AC007255.1 | AC023389.1 | 0.411 | < 0.001 |
| AC007255.1 | IQCK | 0.411 | < 0.001 |
| AC007255.1 | RN7SKP271 | 0.411 | < 0.001 |
| AC007255.1 | AC064807.2 | 0.411 | < 0.001 |
| AC007255.1 | AC006330.1 | 0.410 | < 0.001 |
| AC007255.1 | ZNF700 | 0.410 | < 0.001 |
| AC007255.1 | SIRT1 | 0.410 | < 0.001 |
| AC007255.1 | AC005013.1 | 0.410 | < 0.001 |
| AC007255.1 | AC090948.1 | 0.410 | < 0.001 |
| AC007255.1 | ZXDB | 0.410 | < 0.001 |
| AC007255.1 | RN7SKP281 | 0.410 | < 0.001 |
| AC007255.1 | AC011476.3 | 0.410 | < 0.001 |
| AC007255.1 | KCNA10 | 0.410 | < 0.001 |
| AC007255.1 | TYRO3P | 0.410 | < 0.001 |
| AC007255.1 | CEACAM3 | 0.410 | < 0.001 |
| AC007255.1 | AL121753.2 | 0.410 | < 0.001 |
| AC007255.1 | PHF8 | 0.410 | < 0.001 |
| AC007255.1 | PRPF38B | 0.410 | < 0.001 |
| AC007255.1 | AC011465.1 | 0.410 | < 0.001 |
| AC007255.1 | AC022182.1 | 0.410 | < 0.001 |
| AC007255.1 | STEAP2-AS1 | 0.410 | < 0.001 |
| AC007255.1 | ADAM9 | 0.410 | < 0.001 |
| AC007255.1 | AC104819.1 | 0.410 | < 0.001 |
| AC007255.1 | XIAP-AS1 | 0.410 | < 0.001 |
| AC007255.1 | FOXP1-AS1 | 0.410 | < 0.001 |
| AC007255.1 | SAP25 | 0.410 | < 0.001 |
| AC007255.1 | AC016831.4 | 0.410 | < 0.001 |
| AC007255.1 | AC129507.4 | 0.409 | < 0.001 |
| AC007255.1 | AC113398.2 | 0.409 | < 0.001 |
| AC007255.1 | UVRAG-DT | 0.409 | < 0.001 |
| AC007255.1 | F2RL3 | 0.409 | < 0.001 |
| AC007255.1 | SOCS6 | 0.409 | < 0.001 |
| AC007255.1 | ABCA11P | 0.409 | < 0.001 |
| AC007255.1 | CFAP43 | 0.409 | < 0.001 |
| AC007255.1 | AC118754.1 | 0.409 | < 0.001 |
| AC007255.1 | AC026124.2 | 0.409 | < 0.001 |
| AC007255.1 | LDHAL6B | 0.409 | < 0.001 |
| AC007255.1 | CRTC3-AS1 | 0.409 | < 0.001 |
| AC007255.1 | KIF3AP1 | 0.409 | < 0.001 |
| AC007255.1 | AC022211.4 | 0.409 | < 0.001 |
| AC007255.1 | RN7SL502P | 0.409 | < 0.001 |
| AC007255.1 | SIPA1L3 | 0.409 | < 0.001 |
| AC007255.1 | PPIL6 | 0.409 | < 0.001 |
| AC007255.1 | AL513366.1 | 0.409 | < 0.001 |
| AC007255.1 | AL008726.1 | 0.409 | < 0.001 |
| AC007255.1 | PPP4R3B | 0.409 | < 0.001 |
| AC007255.1 | AC092919.3 | 0.409 | < 0.001 |
| AC007255.1 | ARMCX3 | 0.409 | < 0.001 |
| AC007255.1 | SNRPCP19 | 0.409 | < 0.001 |
| AC007255.1 | RNU6-652P | 0.409 | < 0.001 |
| AC007255.1 | RNA5SP37 | 0.408 | < 0.001 |
| AC007255.1 | CPT2 | 0.408 | < 0.001 |
| AC007255.1 | GORASP2 | 0.408 | < 0.001 |
| AC007255.1 | BICDL2 | 0.408 | < 0.001 |
| AC007255.1 | KCNIP2 | 0.408 | < 0.001 |
| AC007255.1 | CABP1 | 0.408 | < 0.001 |
| AC007255.1 | C1QTNF1-AS1 | 0.408 | < 0.001 |
| AC007255.1 | AC053513.2 | 0.408 | < 0.001 |
| AC007255.1 | RSPH9 | 0.408 | < 0.001 |
| AC007255.1 | CASP9 | 0.408 | < 0.001 |
| AC007255.1 | SPTLC2 | 0.408 | < 0.001 |
| AC007255.1 | ARGFXP2 | 0.408 | < 0.001 |
| AC007255.1 | ANXA10 | 0.408 | < 0.001 |
| AC007255.1 | AC073210.1 | 0.408 | < 0.001 |
| AC007255.1 | STAT5A | 0.408 | < 0.001 |
| AC007255.1 | SORD2P | 0.408 | < 0.001 |
| AC007255.1 | YAE1 | 0.408 | < 0.001 |
| AC007255.1 | ENPP7P5 | 0.408 | < 0.001 |
| AC007255.1 | FRMD1 | 0.408 | < 0.001 |
| AC007255.1 | CCL25 | 0.408 | < 0.001 |
| AC007255.1 | RNF113B | 0.408 | < 0.001 |
| AC007255.1 | RTEL1-TNFRSF6B | 0.407 | < 0.001 |
| AC007255.1 | HECTD3 | 0.407 | < 0.001 |
| AC007255.1 | PRR7-AS1 | 0.407 | < 0.001 |
| AC007255.1 | AC127071.1 | 0.407 | < 0.001 |
| AC007255.1 | AP000944.1 | 0.407 | < 0.001 |
| AC007255.1 | PAIP1P1 | 0.407 | < 0.001 |
| AC007255.1 | ATG7 | 0.407 | < 0.001 |
| AC007255.1 | PDE4DIP | 0.407 | < 0.001 |
| AC007255.1 | BTC | 0.407 | < 0.001 |
| AC007255.1 | RNF213 | 0.407 | < 0.001 |
| AC007255.1 | LINC02691 | 0.407 | < 0.001 |
| AC007255.1 | VTN | 0.407 | < 0.001 |
| AC007255.1 | SLC2A3P2 | 0.407 | < 0.001 |
| AC007255.1 | AC090695.2 | 0.407 | < 0.001 |
| AC007255.1 | DDTP1 | 0.407 | < 0.001 |
| AC007255.1 | RN7SKP37 | 0.407 | < 0.001 |
| AC007255.1 | MANSC1 | 0.407 | < 0.001 |
| AC007255.1 | AC005829.1 | 0.407 | < 0.001 |
| AC007255.1 | YTHDF1 | 0.407 | < 0.001 |
| AC007255.1 | LIME1 | 0.407 | < 0.001 |
| AC007255.1 | NEURL2 | 0.407 | < 0.001 |
| AC007255.1 | DLGAP4-AS1 | 0.407 | < 0.001 |
| AC007255.1 | GHRHR | 0.407 | < 0.001 |
| AC007255.1 | NEU3 | 0.407 | < 0.001 |
| AC007255.1 | SPIN3 | 0.407 | < 0.001 |
| AC007255.1 | AL451050.2 | 0.407 | < 0.001 |
| AC007255.1 | TRIM8 | 0.407 | < 0.001 |
| AC007255.1 | AP000866.3 | 0.407 | < 0.001 |
| AC007255.1 | NRGN | 0.407 | < 0.001 |
| AC007255.1 | AC016722.2 | 0.406 | < 0.001 |
| AC007255.1 | AC106772.2 | 0.406 | < 0.001 |
| AC007255.1 | AC023830.1 | 0.406 | < 0.001 |
| AC007255.1 | AC004134.1 | 0.406 | < 0.001 |
| AC007255.1 | MAP7 | 0.406 | < 0.001 |
| AC007255.1 | TCEA3 | 0.406 | < 0.001 |
| AC007255.1 | AC020987.1 | 0.406 | < 0.001 |
| AC007255.1 | F7 | 0.406 | < 0.001 |
| AC007255.1 | MAN1A1 | 0.406 | < 0.001 |
| AC007255.1 | AL512413.1 | 0.406 | < 0.001 |
| AC007255.1 | AC100803.3 | 0.406 | < 0.001 |
| AC007255.1 | TGM2 | 0.406 | < 0.001 |
| AC007255.1 | PRORSD1P | 0.406 | < 0.001 |
| AC007255.1 | ANKRD24 | 0.406 | < 0.001 |
| AC007255.1 | ZKSCAN1 | 0.406 | < 0.001 |
| AC007255.1 | CFAP69 | 0.406 | < 0.001 |
| AC007255.1 | FCF1P2 | 0.406 | < 0.001 |
| AC007255.1 | AL356124.1 | 0.406 | < 0.001 |
| AC007255.1 | CTAGE7P | 0.406 | < 0.001 |
| AC007255.1 | AGAP9 | 0.406 | < 0.001 |
| AC007255.1 | AIMP1P1 | 0.406 | < 0.001 |
| AC007255.1 | MIR4681 | 0.406 | < 0.001 |
| AC007255.1 | AC025034.1 | 0.406 | < 0.001 |
| AC007255.1 | SNORD7 | 0.406 | < 0.001 |
| AC007255.1 | AC024941.2 | 0.406 | < 0.001 |
| AC007255.1 | AC145207.1 | 0.406 | < 0.001 |
| AC007255.1 | MCCD1P1 | 0.405 | < 0.001 |
| AC007255.1 | MTURN | 0.405 | < 0.001 |
| AC007255.1 | MFSD13A | 0.405 | < 0.001 |
| AC007255.1 | AC091588.2 | 0.405 | < 0.001 |
| AC007255.1 | RTEL1 | 0.405 | < 0.001 |
| AC007255.1 | PSEN1 | 0.405 | < 0.001 |
| AC007255.1 | TSSK3 | 0.405 | < 0.001 |
| AC007255.1 | SNORA71A | 0.405 | < 0.001 |
| AC007255.1 | AL662795.2 | 0.405 | < 0.001 |
| AC007255.1 | LMBRD2 | 0.405 | < 0.001 |
| AC007255.1 | SLC9A3R2 | 0.405 | < 0.001 |
| AC007255.1 | LPIN1 | 0.405 | < 0.001 |
| AC007255.1 | AC139887.4 | 0.405 | < 0.001 |
| AC007255.1 | AC011455.1 | 0.405 | < 0.001 |
| AC007255.1 | RPL31P50 | 0.405 | < 0.001 |
| AC007255.1 | CASP5 | 0.405 | < 0.001 |
| AC007255.1 | TSPEAR-AS1 | 0.405 | < 0.001 |
| AC007255.1 | FOXH1 | 0.405 | < 0.001 |
| AC007255.1 | TRA2A | 0.405 | < 0.001 |
| AC007255.1 | PUS7L | 0.405 | < 0.001 |
| AC007255.1 | AP003084.1 | 0.405 | < 0.001 |
| AC007255.1 | LINC01232 | 0.405 | < 0.001 |
| AC007255.1 | SSBP3-AS1 | 0.405 | < 0.001 |
| AC007255.1 | CNPY2 | 0.405 | < 0.001 |
| AC007255.1 | AC008268.1 | 0.405 | < 0.001 |
| AC007255.1 | RSKR | 0.405 | < 0.001 |
| AC007255.1 | MIR569 | 0.404 | < 0.001 |
| AC007255.1 | AC016831.1 | 0.404 | < 0.001 |
| AC007255.1 | AC092681.1 | 0.404 | < 0.001 |
| AC007255.1 | LINC01836 | 0.404 | < 0.001 |
| AC007255.1 | PRICKLE4 | 0.404 | < 0.001 |
| AC007255.1 | FKBP11 | 0.404 | < 0.001 |
| AC007255.1 | AC084782.2 | 0.404 | < 0.001 |
| AC007255.1 | ZNF165 | 0.404 | < 0.001 |
| AC007255.1 | MIR1972-2 | 0.404 | < 0.001 |
| AC007255.1 | NAA16 | 0.404 | < 0.001 |
| AC007255.1 | AC073655.2 | 0.404 | < 0.001 |
| AC007255.1 | AL136296.1 | 0.404 | < 0.001 |
| AC007255.1 | AC025430.1 | 0.404 | < 0.001 |
| AC007255.1 | CCDC66 | 0.404 | < 0.001 |
| AC007255.1 | PLEKHH2 | 0.404 | < 0.001 |
| AC007255.1 | UCN3 | 0.404 | < 0.001 |
| AC007255.1 | AL117350.2 | 0.404 | < 0.001 |
| AC007255.1 | CUZD1 | 0.404 | < 0.001 |
| AC007255.1 | AC008417.1 | 0.404 | < 0.001 |
| AC007255.1 | CCL14 | 0.404 | < 0.001 |
| AC007255.1 | AC020911.1 | 0.404 | < 0.001 |
| AC007255.1 | EFCAB12 | 0.404 | < 0.001 |
| AC007255.1 | AC015845.1 | 0.404 | < 0.001 |
| AC007255.1 | AC087857.1 | 0.403 | < 0.001 |
| AC007255.1 | HNRNPH1P1 | 0.403 | < 0.001 |
| AC007255.1 | INTS4P2 | 0.403 | < 0.001 |
| AC007255.1 | NME9 | 0.403 | < 0.001 |
| AC007255.1 | IL17RE | 0.403 | < 0.001 |
| AC007255.1 | MIR3177 | 0.403 | < 0.001 |
| AC007255.1 | AC090971.4 | 0.403 | < 0.001 |
| AC007255.1 | SUGT1 | 0.403 | < 0.001 |
| AC007255.1 | AL390961.2 | 0.403 | < 0.001 |
| AC007255.1 | AIFM2 | 0.403 | < 0.001 |
| AC007255.1 | PSMD6-AS2 | 0.403 | < 0.001 |
| AC007255.1 | CYB5B | 0.403 | < 0.001 |
| AC007255.1 | AL590729.1 | 0.403 | < 0.001 |
| AC007255.1 | ATG4A | 0.403 | < 0.001 |
| AC007255.1 | AC091057.2 | 0.403 | < 0.001 |
| AC007255.1 | AL137230.2 | 0.403 | < 0.001 |
| AC007255.1 | AL353801.1 | 0.403 | < 0.001 |
| AC007255.1 | LINC01534 | 0.403 | < 0.001 |
| AC007255.1 | PRC1-AS1 | 0.403 | < 0.001 |
| AC007255.1 | AL355112.1 | 0.403 | < 0.001 |
| AC007255.1 | RMC1 | 0.403 | < 0.001 |
| AC007255.1 | KRTCAP3 | 0.403 | < 0.001 |
| AC007255.1 | RSAD1 | 0.403 | < 0.001 |
| AC007255.1 | AC002553.1 | 0.403 | < 0.001 |
| AC007255.1 | BMF | 0.403 | < 0.001 |
| AC007255.1 | GMCL1 | 0.403 | < 0.001 |
| AC007255.1 | RN7SL803P | 0.403 | < 0.001 |
| AC007255.1 | ST6GALNAC4P1 | 0.402 | < 0.001 |
| AC007255.1 | PPARA | 0.402 | < 0.001 |
| AC007255.1 | AP003351.1 | 0.402 | < 0.001 |
| AC007255.1 | AP001273.1 | 0.402 | < 0.001 |
| AC007255.1 | MTATP6P27 | 0.402 | < 0.001 |
| AC007255.1 | ANKRD18EP | 0.402 | < 0.001 |
| AC007255.1 | AC021739.1 | 0.402 | < 0.001 |
| AC007255.1 | RPL36AP41 | 0.402 | < 0.001 |
| AC007255.1 | CEBPA-DT | 0.402 | < 0.001 |
| AC007255.1 | AC009269.5 | 0.402 | < 0.001 |
| AC007255.1 | ARL6IP1 | 0.402 | < 0.001 |
| AC007255.1 | FLNB-AS1 | 0.402 | < 0.001 |
| AC007255.1 | LINC00244 | 0.402 | < 0.001 |
| AC007255.1 | EIF1AXP2 | 0.402 | < 0.001 |
| AC007255.1 | AMH | 0.402 | < 0.001 |
| AC007255.1 | AC079336.7 | 0.402 | < 0.001 |
| AC007255.1 | PHF12 | 0.402 | < 0.001 |
| AC007255.1 | PTPN3 | 0.402 | < 0.001 |
| AC007255.1 | ACOT4 | 0.402 | < 0.001 |
| AC007255.1 | POMGNT2 | 0.402 | < 0.001 |
| AC007255.1 | ITPRID1 | 0.402 | < 0.001 |
| AC007255.1 | NFYA | 0.402 | < 0.001 |
| AC007255.1 | AP000873.2 | 0.402 | < 0.001 |
| AC007255.1 | HIPK1 | 0.401 | < 0.001 |
| AC007255.1 | TMEM86B | 0.401 | < 0.001 |
| AC007255.1 | GNA11 | 0.401 | < 0.001 |
| AC007255.1 | AC118344.1 | 0.401 | < 0.001 |
| AC007255.1 | N6AMT1 | 0.401 | < 0.001 |
| AC007255.1 | AC093010.3 | 0.401 | < 0.001 |
| AC007255.1 | ARSH | 0.401 | < 0.001 |
| AC007255.1 | AL158212.2 | 0.401 | < 0.001 |
| AC007255.1 | PHBP8 | 0.401 | < 0.001 |
| AC007255.1 | LINC01411 | 0.401 | < 0.001 |
| AC007255.1 | AC124045.1 | 0.401 | < 0.001 |
| AC007255.1 | KIAA1549 | 0.401 | < 0.001 |
| AC007255.1 | TFB1M | 0.401 | < 0.001 |
| AC007255.1 | GALNT10 | 0.401 | < 0.001 |
| AC007255.1 | AC092287.1 | 0.401 | < 0.001 |
| AC007255.1 | LENG8-AS1 | 0.401 | < 0.001 |
| AC007255.1 | RTKN2 | 0.401 | < 0.001 |
| AC007255.1 | AL049873.2 | 0.401 | < 0.001 |
| AC007255.1 | CHCHD7 | 0.401 | < 0.001 |
| AC007255.1 | AC007038.1 | 0.401 | < 0.001 |
| AC007255.1 | LIPC | 0.401 | < 0.001 |
| AC007255.1 | AC008610.1 | 0.401 | < 0.001 |
| AC007255.1 | CDK11B | 0.401 | < 0.001 |
| AC007255.1 | LMTK2 | 0.401 | < 0.001 |
| AC007255.1 | TRIM66 | 0.401 | < 0.001 |
