# Supplementary material for: LncRNA AC007255.1, an immune-related prognostic enhancer RNA in esophageal cancer
Source: PeerJ. 2021 Jul 14;9:e11698. doi: 10.7717/peerj.11698 (PMC8286057; doi:10.7717/peerj.11698)
Supplement: Supplemental Information 3 [file peerj-09-11698-s003.docx]

| Table S3. Clinicopathological characteristic information of ESCA patients from TCGA database. | | |
| --- | --- | --- |
| Covariates | Type | tableStat |
| Age | <60 | 74(45.96%) |
|  | >=60 | 87(54.04%) |
| Stage | Stage I | 3(1.85%) |
|  | Stage II | 21(12.96%) |
|  | Stage III | 19(11.73%) |
|  | Stage IV | 10(6.17%) |
|  | unknow | 109(67.28%) |
| Tumor_cental_location | Distal | 113(69.75%) |
|  | Mid | 42(25.93%) |
|  | Proximal | 6(3.7%) |
|  | unknow | 1(0.62%) |
| Grade | G1 | 16(9.88%) |
|  | G2 | 66(40.74%) |
|  | G3 | 44(27.16%) |
|  | unknow | 36(22.22%) |
| M | M0 | 121(74.69%) |
|  | M1 | 8(4.94%) |
|  | unknow | 33(20.37%) |
| N | N0 | 66(40.74%) |
|  | N1 | 63(38.89%) |
|  | N2 | 9(5.56%) |
|  | N3 | 6(3.7%) |
|  | unknow | 18(11.11%) |
| T | T1 | 27(16.67%) |
|  | T2 | 37(22.84%) |
|  | T3 | 77(47.53%) |
|  | T4 | 4(2.47%) |
|  | unknow | 17(10.49%) |
| Cancer_status | TUMOR FREE | 61(37.65%) |
|  | WITH TUMOR | 35(21.6%) |
|  | unknow | 66(40.74%) |
| Reflux_history | NO | 84(51.85%) |
|  | YES | 52(32.1%) |
|  | unknow | 26(16.05%) |
| Gender | female | 23(14.2%) |
|  | male | 139(85.8%) |
| Race | asian | 38(23.46%) |
|  | black or african american | 6(3.7%) |
|  | white | 100(61.73%) |
|  | unknow | 18(11.11%) |
