# Supplementary material for: LncRNA AC007255.1, an immune-related prognostic enhancer RNA in esophageal cancer
Source: PeerJ. 2021 Jul 14;9:e11698. doi: 10.7717/peerj.11698 (PMC8286057; doi:10.7717/peerj.11698)
Supplement: Supplemental Information 4 [file peerj-09-11698-s004.docx]

| Table S4. 78 genes associated AC007255.1 that are enriched in neutrophil activation involved in immune response (Spearman’s rank correlation coefficient r>0.4 and adjusted p<0.05). | | | | | |
| --- | --- | --- | --- | --- | --- |
| Gene Symbol | Spearman Correlation Coefficient r | Gene Symbol | Spearman Correlation Coefficient r | Gene Symbol | Spearman Correlation Coefficient r |
| CTSA | 0.508 | PSEN1 | 0.405 | DGAT1 | 0.496 |
| PTPRB | 0.568 | GUSB | 0.596 | MVP | 0.496 |
| LYZ | 0.584 | SERPINA1 | 0.554 | LGALS3 | 0.581 |
| ANXA3 | 0.46 | PTPRJ | 0.655 | ORM2 | 0.425 |
| COTL1 | 0.479 | CTSS | 0.564 | STBD1 | 0.464 |
| RHOF | 0.476 | PIGR | 0.633 | APEH | 0.457 |
| SDCBP | 0.417 | CEACAM6 | 0.533 | HEXB | 0.455 |
| ATP8A1 | 0.625 | CEACAM3 | 0.41 | IQGAP2 | 0.589 |
| DDOST | 0.467 | CTSH | 0.514 | OLFM4 | 0.548 |
| CEACAM8 | 0.47 | CD55 | 0.53 | CEACAM1 | 0.451 |
| TTR | 0.428 | PRKCD | 0.495 | ADGRE5 | 0.633 |
| MLEC | 0.628 | GCA | 0.616 | CYSTM1 | 0.692 |
| TMEM63A | 0.758 | SNAP23 | 0.595 | DNAJC3 | 0.518 |
| CYBA | 0.534 | VNN1 | 0.555 | ACAA1 | 0.5 |
| PTPRN2 | 0.552 | FRK | 0.544 | LCN2 | 0.522 |
| GHDC | 0.609 | ENPP4 | 0.676 | NHLRC3 | 0.605 |
| PRSS3 | 0.612 | ATP11A | 0.575 | IDH1 | 0.463 |
| ORMDL3 | 0.649 | GNS | 0.433 | ASAH1 | 0.415 |
| TNFRSF1B | 0.49 | FUCA2 | 0.595 | AGPAT2 | 0.489 |
| APAF1 | 0.637 | SPTAN1 | 0.559 | SVIP | 0.643 |
| FUCA1 | 0.7 | SLC27A2 | 0.539 | CAT | 0.481 |
| PRSS2 | 0.485 | ALDH3B1 | 0.652 | RNASET2 | 0.642 |
| ATG7 | 0.407 | CANT1 | 0.586 | PTGES2 | 0.416 |
| RAB37 | 0.536 | PLAC8 | 0.573 | GLB1 | 0.656 |
| MGAM | 0.586 | CD63 | 0.516 | SERPINB6 | 0.627 |
| S100P | 0.631 | AOC1 | 0.621 | DNASE1 | 0.412 |
